# Discovery of a Neuroprotective Diosgenin Derivative as a Novel Antidepressant Candidate Targeting LPS-TLR4 Signaling

**DOI:** 10.1021/acs.jmedchem.5c02981

**Published:** 2026-02-02

**Authors:** Younghun Yoo, Soo Yeon Baek, Hyelim Lee, Jeehee Lee, Hyowon Lee, Haeun Lee, Hyeonji Ma, Yujin Kim, Hoon-Seong Choi, Jeong Tae Lee, Jae Yeol Lee, Min-Ho Nam, Sanghee Lee, Byungsun Jeon

**Affiliations:** a Medicinal Materials Research Center, Biomedical Research Division, 58975 Korea Institute of Science and Technology, Seoul 02792, Republic of Korea; b Division of Bio-Medical Science and Technology, KIST School, University of Science and Technology, Seoul 02792, Republic of Korea; c KHU-KIST Department of Converging Science and Technology, 26723 Kyunghee University, Seoul 02447, Republic of Korea; d Center for Brain Disorders, Brain Science Institute, 58975 Korea Institute of Science and Technology, Seoul 02792, Republic of Korea; e Research Animal Resources Center, Research Resources Division, 58975 Korea Institute of Science and Technology, Seoul 02792, Republic of Korea; f Department of Chemistry and Institute of Applied Chemistry, 26727 Hallym University, Chuncheon 24252, Republic of Korea; g Department of HY-KIST Bio-convergence, Hanyang University, Seoul 04763, Republic of Korea; h Research Institute for Basic Sciences and Department of Chemistry, College of Sciences, Kyung Hee University, Seoul 02447, Republic of Korea

## Abstract

Depression is a widespread and increasing mental disorder, yet current antidepressants, including tricyclic antidepressants (TCAs) and selective serotonin reuptake inhibitors (SSRIs), often cause notable side effects and limited efficacy. Hence, safer therapeutic options are needed. Diosgenin, a phytosteroid sapogenin from the Dioscoreaceae plants, has demonstrated therapeutic potential for neurological disorders but is hindered by unclear target mechanism, poor solubility, and limited bioavailability. Here, we synthesized diosgenin derivatives and evaluated their biological activities. Among them, compound **8** exhibited the highest therapeutic index (TI = 19.8), strongly inhibiting LPS-induced NO production with minimal cytotoxicity. Compound **8** suppressed proinflammatory gene expression, showed neuroprotective effects *in vitro*, ameliorated LPS-induced reactive astrogliosis and microgliosis *in vivo*, and alleviated LPS-induced depressive-like behaviors in mice. Computational docking and centrifugal ultrafiltration assays identified LY96 as a potential target, suggesting modulation of LPS-TLR4 signaling. Collectively, these findings indicate that compound **8** holds promise as a safer antidepressant candidate.

## Graphical abstract

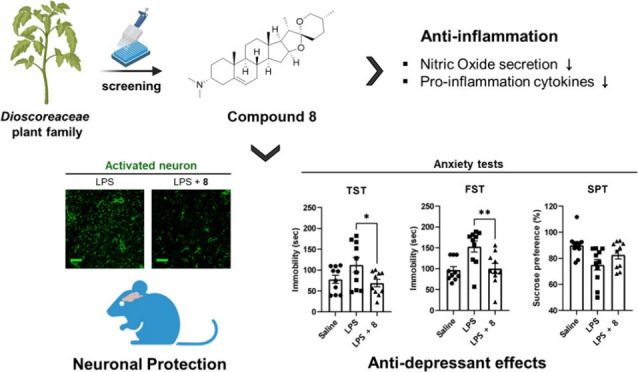

## Introduction

Depression is a common mental illness affecting approximately 300 million people worldwide, with its prevalence steadily rising.^1^ Typical symptoms include persistent sadness and a loss of interest or pleasure in activities.^1,2^ While the exact etiology remains unclear, depression is thought to arise from a complex interplay of biochemical, genetic, and environmental factors, similar to other mental disorders.^3^ Tricyclic antidepressants (TCAs) and selective serotonin reuptake inhibitors (SSRIs) are the cornerstone treatments in clinical practice.^4,5^ However, their utility is often compromised by notable side effects, which limit their efficacy, prevent a full resolution of depression, and fail to address the diverse needs of individual patients.^4,5^ This underscores an urgent need to develop safer, more effective antidepressants while further elucidating the pathophysiology of depression.

Diosgenin (DG, **2**), a steroidal saponin, is a hydrolyzed product of dioscin (**1**) produced by the plant family of *Dioscoreaceae*.^6,7^ Like dioscin, diosgenin, a phytosteroid sapogenin and the aglycone of dioscin, exhibits multiple pharmacological activities such as antitumor, antimicrobial, anti-inflammatory, antioxidative, and tissue-protective properties.^7–14^ Recent studies have highlighted its potential in preventing and treating neurological disorders such as Alzheimer’s disease (AD), Parkinson’s disease (PD), and neuroinflammation.^11,15–17^ Neuroinflammation, a complex process involving immune activation in the central nervous system (CNS), is increasingly recognized as a crucial factor to the development of depression.^18–20^ Targeting inflammation thus offers a promising avenue for developing novel antidepressants.^21^ Previous studies have demonstrated that diosgenin possesses antidepressant-like properties, including improvements in both the forced swimming test (FST) and tail suspension test (TST) in behavioral models, as well as reduction in proinflammatory cytokine levels and restoration of impaired neurogenesis under chronic restraint stress.^22,23^ The therapeutic mechanisms of diosgenin in neurological diseases and neuroinflammation have been considered as the mediation of various signaling pathways including TLR, NF-κB, JNK, and MAPK.^13,24,25^

Despite its promising pharmacological profiles, however, some drawbacks including the poor solubility in aqueous media and low bioavailability obstruct its clinical application.^26^ Notably, dioscin is known to undergo metabolic hydrolysis *in vivo* to generate diosgenin as the pharmacologically relevant aglycone, supporting diosgenin as an appropriated scaffold for further optimization. Structural modification of the diosgenin scaffold therefore represents a rational strategy to improve its drug-like properties while preserving biological activity. In this context, modification at the sugar-linking oxygen position offers a synthetically accessible handle for introducing heteroatom-containing substituents that can modulate polarity, hydrogen-bonding capacity, and overall physicochemical properties without disrupting the steroidal framework. Based on these considerations, here, we designed and synthesized a series of diosgenin derivatives and to evaluate their neuroinflammatory modulation, cytotoxicity, and antidepressant-related potential using complementary *in vitro*, *in vivo*, and computational approaches.

## Results and Discussion

### Characterization and Anti-inflammatory Effect of Dioscin

To evaluate the anti-inflammatory properties of dioscin,^27^ we assessed its impact on nuclear factor kappa B (NF-κB) signaling using THP-1 human monocyte cells engineered with a secreted embryonic alkaline phosphatase (SEAP) reporter for NF-κB pathway. Exposure to lipopolysaccharide (LPS) activated the NF-κB signaling pathway. Subsequent treatment with dioscin, at concentrations ranging from 6.25 to 100 μM in the presence of LPS (100 ng/mL), inhibited this pathway in a dose-dependent manner (Figure 1a). We also examined the effects of dioscin on reactive nitrogen species, key markers of neuroinflammation, in BV-2 mouse microglia cells. Dioscin reduced LPS-induced nitric oxide (NO) production in a dose-dependent manner (Figure 1b). These findings confirm its strong suppression of key inflammatory mediators, supporting its potential utility in neuroinflammation-associated conditions such as depression. However, the therapeutic window of dioscin was severely restricted by pronounced cytotoxicity. Substantial reductions in cell viability were observed across the tested concentration range in both THP-1 and BV-2 cells (Figures 1a and 1b). This narrow safety margin limits the clinical translational potential of dioscin despite its promising activity. Given the pronounced cytotoxicity observed for dioscin, subsequent studies focused on diosgenin, the aglycone of dioscin (Figure 1c). Diosgenin retains many pharmacological properties of dioscin, including anti-inflammatory and neuroprotective effects, but is constrained by poor aqueous solubility and low oral bioavailability.

### Synthesis

Given the antineuroinflammatory properties of dioscin, we synthesized a series of diosgenin derivatives starting from diosgenin through activation and modification pathways described in Scheme 1. To examine the influence of stereochemistry at the (*S*)-secondary alcohol of diosgenin, we activated the hydroxyl group using TsCl and pyridine in the presence of a catalytic amount of DMAP. Subsequent nucleophilic substitution with NaOH afforded the (*R*)-diastereomer of diosgenin in 62% yield over two steps. To introduce an amino group, we performed a substitution reaction with NaN_3_ on tosylated diosgenin, followed by a Staudinger reaction with PPh_3_, achieving 59% yield over three steps. Methylation of the oxygen or nitrogen atoms was carried out using iodomethane or the Eschweiler-Clarke protocol (employing formaldehyde and formic acid under reductive conditions) or by carbamate formation with Boc anhydride followed by hydride reduction,^28^ producing *O*- or *N*-methylated derivatives in 82–97% yields. Thiodiosgenin (**14**) was synthesized via an Appel reaction with CBr_4_ and PPh_3_ in 90% yield. The resulting bromo intermediate was then substituted with thioacetate, followed by base-catalyzed methanolysis, yielding thiodiosgenin with retained stereochemistry in 54% yield over two steps. In addition, to further modulate the physicochemical properties of the diosgenin scaffold, a glucose conjugated derivative was synthesized starting from diosgenin (**2**). Glycosylation of the secondary alcohol was achieved using acetobromo-α-d-glucose in the presence of silver carbonate, affording peracetylated glucoside in 60% yield. Subsequent deprotection under mild basic conditions using NaOMe provided the corresponding free glucose-conjugated diosgenin derivative in 66% yield. All compounds were fully characterized, enabling systematic evaluation of structure–activity relationships.

**Figure 1:**
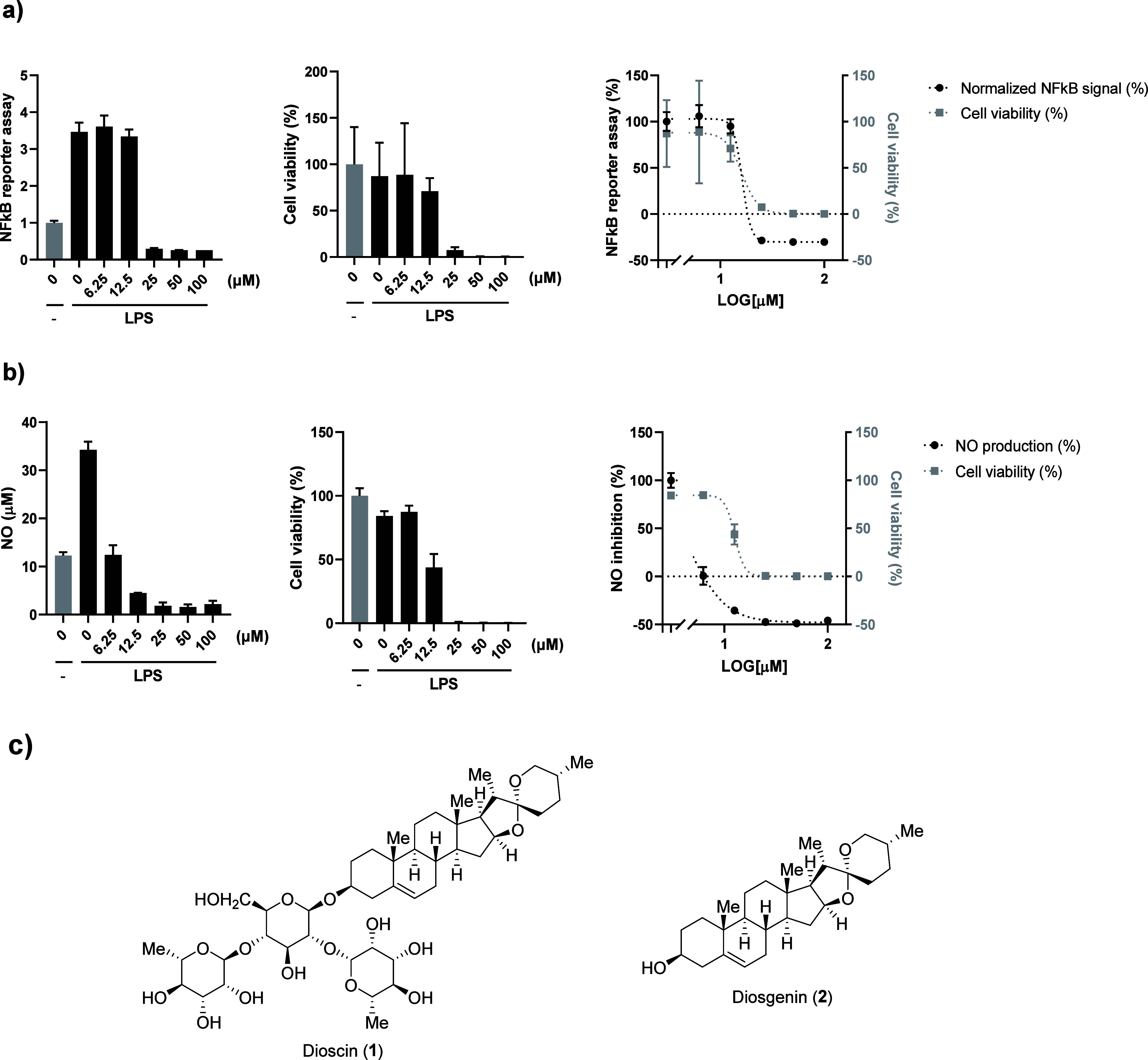
Anti-inflammatory effect and safety window of dioscin. a) NF-κB reporter signal (left), cell viability (center) and merged graph to compare safety window (right) of dioscin in THP-1 cells. Cells were pretreated with dioscin or DMSO for 1 h, and then treated with LPS (100 ng/mL) for 24 h. b) Detection of NO releasing (left), cell viability (center) and merged graph to compare safety window (right) of dioscin in BV-2 cells. Cells were pretreated with dioscin or DMSO for 1 h, and then treated with LPS (100 ng/mL) for 24 h. Graphs represent mean and SD. c) Structures of dioscin (**1**) and diosgenin (**2**).

**Scheme 1:**
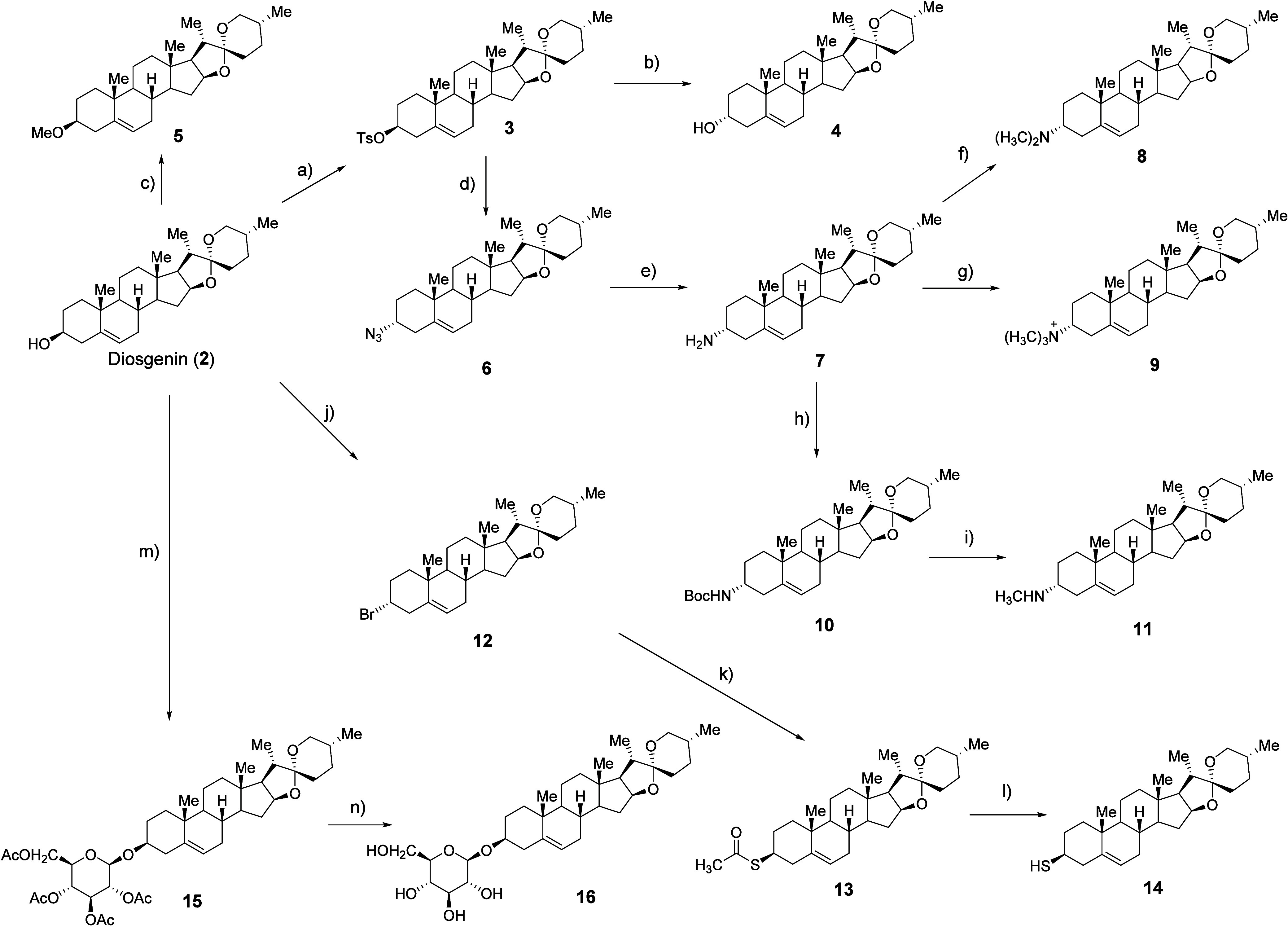
Synthesis of Diosgenin Derivatives^a^

a a) TsCl, DMAP, pyridine, dichloromethane, 0 °C to rt, 24 h. b) NaOH, DMF, 130 °C, 12 h, 62% over two steps. c) NaH, MeI, THF, 60 °C, 18 h, 94%. d) NaN_3_, DMF, 80 °C, 22 h, 69%. e) PPh_3_, THF/H_2_O, 60 °C, 6 h, 85%. f) 1) CH_2_O, HCOOH, MeOH, 30 min 2) NaBH_3_CN, MeOH, rt, 4 h, 88%. g) MeI, K_2_CO_3_, CH_3_CN/dichloromethane, 60 °C, 12 h, 97%. h) Boc_2_O, triethylamine, dichloromethane, rt, 12 h, quant. (i) LAH, THF, 0 °C to reflux, 4 h, 84%. j) CBr_4_, PPh_3_, dichloromethane, 0 °C to rt, 4 h, 90%. k) KSAc, 18-C-6, DMF, 100 °C, 24 h, 67%. l) NaOMe (1 M in MeOH), MeOH, rt, 12 h, 80%. m) Acetobromo-α-d-glucose, Ag_2_CO_3_, dichloromethane, rt, 48 h, 60%. n) NaOMe (0.5 M in MeOH), THF, rt, 12 h, 66%.

### Evaluation of Therapeutic Window by NO Suppression and Cell Viability

To assess the therapeutic safety window, we evaluated the effects of diosgenin derivatives on NO production and cell viability in BV-2 cells exposed to LPS, with results summarized in Table 1 and the Supporting Information. Although diosgenin (**2**, entry 1) and its diastereomer (**4**, entry 3) exhibited substantially weaker NO inhibitory activity and lower cytotoxicity than dioscin (**1**), as indicated by higher IC_50_ and LD_50_ values, their overall therapeutic index (TI) values were similar (TI = 3.0 and 3.3, respectively), reflecting a balance between reduced efficacy and reduced toxicity. These data indicate that inversion of stereochemistry at the secondary alcohol has minimal impact on the overall therapeutic window, suggesting that hydroxyl configuration is not a critical determinant of activity within this scaffold. In contrast, modification of the hydroxyl group led to pronounced changes in both efficacy and cytotoxicity. Diosgenin methyl ether (**5**) afforded a TI value of 1, reflecting increased cell viability but a complete loss of NO inhibitory activity. Modifying the functional group from a hydroxyl to an amino group (**7**) or a thiol group (**14**) dramatically altered the TI values to 0.3 and 7.7, respectively. Unlike the methyl ether derivative, methylation of the amino group enhanced cell viability while preserving the NO inhibitory effect. Notably, dimethylamino diosgenin (**8**) achieved a TI value of 19.8, showing the best inhibitory effect on LPS-induced NO production without cytotoxicity, thus highlighting its superior therapeutic potential among the derivatives tested. Collectively, these structure–activity relationship (SAR) findings demonstrate that strategic functional group modification of the diosgenin scaffold can decouple anti-inflammatory activity from cytotoxicity, establishing compound **8** as the lead candidate. To further validate the physicochemical improvements driving bioavailability, kinetic solubility measurements in pH 7.4 phosphate buffer (1% DMSO) showed that compound **8** exhibited ∼ 25-fold higher apparent solubility than diosgenin (**2**) (SI Figure S37)

**Table 1:** Evaluation of Anti-inflammatory Effect and Cell Viability

entry	compound	IC_50_ (NO inhibition)^a^ (μM)	**LD**_**50**_ **(cell viability)** ^b^ **(μM)**	**therapeutic index** ^c^ **(TI)**
**1**	**DG–OH (** *S* **): diosgenin (2)**	63.8 ± 8.0	191 ± 27.9	3.0
**2**	**dioscin (1)**	4.7 ± 3.9	12.6 ± 0.3	3.3
**3**	**DG–OH (** *R* **, 4)**	85.1 ± 5.2	277.6 ± 39.3	3.3
**4**	**DG-O–CH**_**3**_ **(5)**	866.1 ± 513.4	823.2 ± 298.4	1.0
**5**	**DG-NH**_**2**_ **(7)**	33.4 ± 1.4	10.9 ± 10.2	0.3
**6**	**DG-NH–CH**_**3**_ **(11)**	33.7 ± 0.9	21.0 ± 2.6	0.6
**7**	**DG-N(CH**_**3**_ **)**_**2**_ **(8)**	33.7 ± 16.6	667.5 ± 19.7	**19.8**
**8**	**DG-N** ^**+**^ **(CH**_**3**_ **)**_**3**_ **(9)**	N.D.^d^	N.D.	N.D.
**9**	**DG-Br (12)**	N.D.	N.D.	N.D.
**10**	**DG-SH (14)**	97.3 ± 9.7	747.6 ± 145.9	7.7
**11**	**DG-N**_**3**_ **(6)**	177.7 ± 8.4	605.7 ± 331	3.4
**12**	**DG-OGlc (16)**	<25	62.2 ± 4.5	N.D.

a Griess assay for LPS-induced NO production after individual compound treatment for 24 h in BV-2 cells.

b Cell viability test of each compound in BV-2 cells for 24 h.

c TI value^29^ = LD_50_ (cell viability, μM)/IC_50_ (NO inhibition, μM).

d N.D.: not determinable within the tested concentration range.

### Validation of Antineuroinflammatory Effect

We further investigated the effect of compound **8** against neuroinflammation by monitoring the expression levels of pro-inflammatory genes such as *Il1b*, *Il6*, *Tnfa*, and *Nos2* in BV-2 microglial cells. LPS stimulation considerably upregulated the expression of these genes, but this effect was remarkably attenuated by compound **8** (Figure 2a). Interestingly, compound **8** also enhanced the expression of *Hmox1*, an anti-inflammatory gene, under LPS-stimulated conditions. To validate whether compound **8** inhibited pro-inflammatory cytokine secretion in addition to suppressing gene transcription, we also quantified the extracellular tumor necrosis factor-alpha (TNFα) levels in BV-2 cells using an enzyme-linked immunosorbent assay (ELISA) (Figure 2b). As anticipated, LPS stimulation increased TNFα production relative to untreated controls. However, treatment with compound **8** reduced TNFα levels in a dose-dependent manner. Notably, at a concentration of 200 μM, compound **8** lowered TNFα production to levels comparable to those in LPS untreated cells, with no detectable cytotoxicity as assessed by the MTS assay (SI Figure S32). Together, these results demonstrate that compound **8** effectively mitigates LPS-induced neuroinflammation in microglia cells. These findings suggest a dual mechanism, inhibiting pro-inflammatory pathways while promoting anti-inflammatory responses, consistent with the known involvement of NF-κB and MAPK pathways in inflammation.^24^

**Figure 2:**
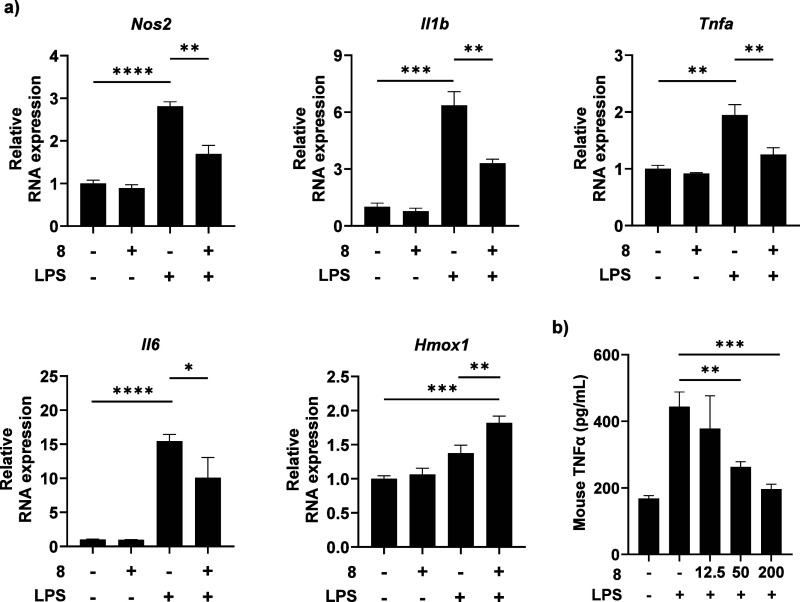
Effect on inflammatory markers by compound **8** treatment. a) Pro-inflammatory and anti-inflammatory gene expression in BV-2 cells after LPS or compound **8** treatment. Cells were pretreated with compound **8** (50 μM) for 1 h, and then treated with LPS (200 ng/mL) for 8 h. Relative expression was normalized by GAPDH as an internal standard. Graph shows mean and RQmax. b) ELISA analysis for pro-inflammatory cytokine, TNFα in BV-2 cells. Cells were pretreated with indicated concentration of compound **8** for 1 h, and then treated with LPS (100 ng/mL) for 24 h. Graph shows mean and SD. Unpaired *t* test for statistical analysis.

### Identification and Validation of LY96 as a Molecular Target for Compound **8** in Modulating LPS-Induced Neuroinflammation

To identify the molecular target of compound **8**, we performed reverse docking by screening it against a panel of protein targets, identifying the top 100 candidates based on their Z-Score and AK-score2 energy values, which reflect the strength of ligand-protein interactions.^33^ From this list, we selected a highly ranked protein, lymphocyte antigen 96 (LY96), also known as myeloid differentiation factor 2 (MD-2), due to its established role in the inflammatory signaling pathway. LY96, a protein known to mediate LPS binding to Toll-like receptor 4 (TLR4), demonstrated a relatively high binding affinity for compound **8** in reverse docking simulations, suggesting its potential involvement in modulating LPS-TLR4 interactions.

To validate this interaction experimentally, we performed a centrifugal untrafiltration assay, where compound **8** consistently associated with LY96, indicating stable complex formation (Figure 3c,d). Mechanistically, this interaction is consistent with attenuation of downstream inflammatory signaling. To assess downstream effects, we examined the NF-κB pathway in BV-2 microglial cells, where Western blot analysis revealed a significant reduction in p65 phosphorylation relative to p65 levels in the presence of compound **8** (Figure 3a). Similarly, Western blot analysis showed a marked decrease in inducible nitric oxide synthase (iNOS) expression in LPS-stimulated BV-2 cells treated with compound **8** (Figure 3b).

**Figure 3:**
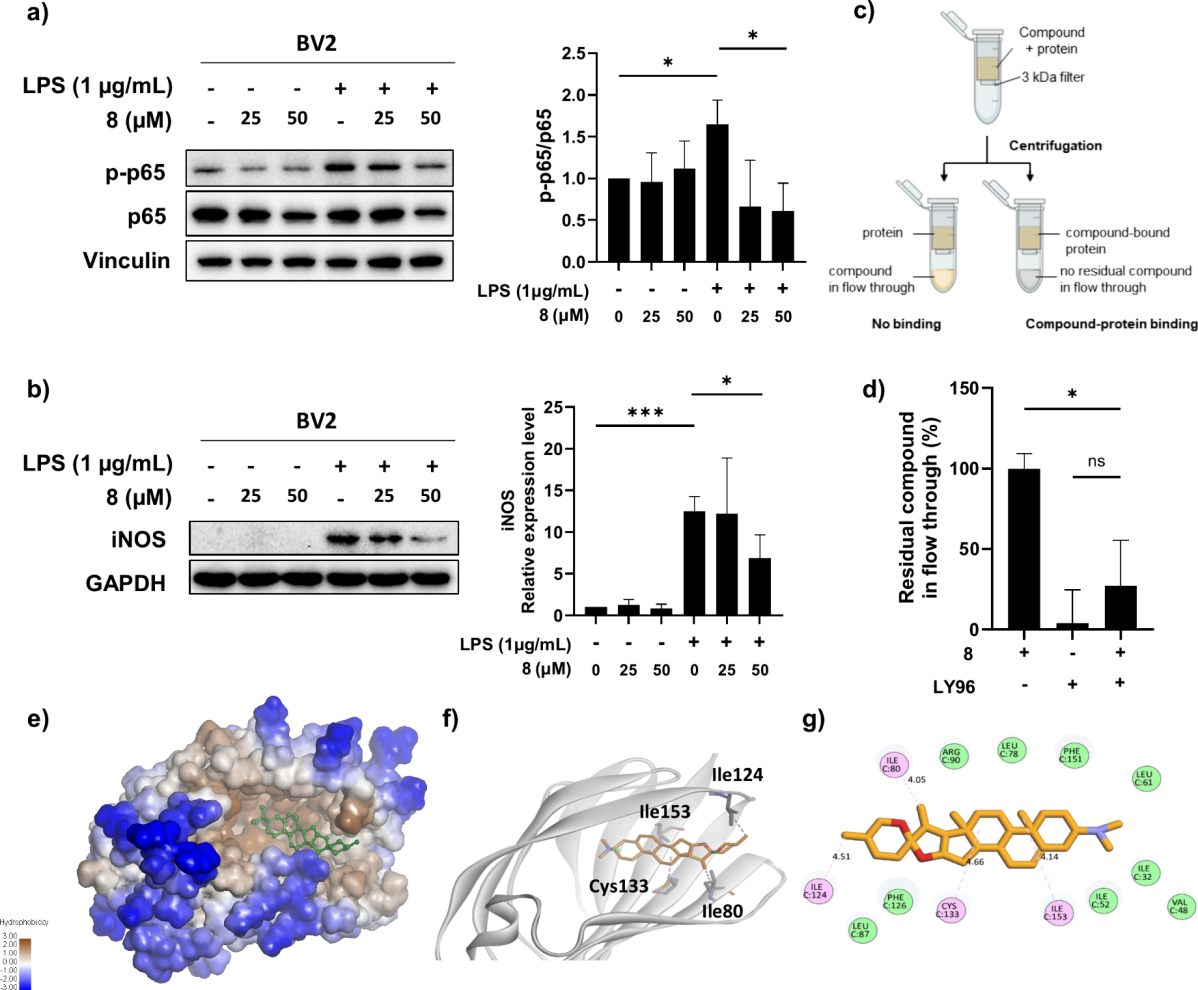
Compound **8** binds to LY96 and exhibits an antineuroinflammatory effect on LPS-induced NF-κB activation. a) Western blot analysis of NF-κB pathway. BV-2 cells were pretreated with compound **8** or DMSO for 2 h, and then treated with LPS (1 μg/mL) for 3 h. The normalized intensities of the phosphorylated p65 relative to their total forms are presented (right). b) Western blot analysis of inducible NOS (iNOS). BV-2 cells were pretreated with compound **8** or DMSO for 2 h, and then treated with LPS (1 μg/mL) for 8 h. The relative expression level of iNOS is presented (right). Each experiment was performed in triplicate. Unpaired *t* test for statistical analysis. c) Scheme of the rapid centrifugal ultrafiltration assay. d) Binding study of compound **8** with human LY96 protein using centrifugal ultrafiltration assay. 10 μM of protein and compound **8** was incubated at room temperature for 10 min. The unbound compound from a 1 min spin was monitored by its UV absorbance at 230 nm. e) Docking model of LY96 with compound **8** (ball and stick), illustrating the binding of compound **8** within the hydrophobic pocket of LY96. Hydrophobicity is represented by a color gradient, with blue indicating highly hydrophilic regions (−3.00) and brown indicating highly hydrophobic regions (+3.00). f) Binding interactions of compound **8** with amino acid residues such as Ile124, Ile153, Ile80, and Cys133 shown in gray at the binding site of LY96 (PDB: 3FXI). g) Two-dimensional diagram of the binding interactions of compound **8** within the binding pocket of LY96. Surrounding amino acid residues are labeled with their respective sequence number and key interacting residues are represented as pink spheres. Other van der Waals interactions are depicted as green spheres. Dashed lines indicate van der Waals interactions with distances labeled in angstroms, highlighting the spatial arrangement and binding affinity within the complex. The compound **8** is shown in stick representation with carbon atoms in orange, oxygen in red, and nitrogen in blue.

To understand the binding mode, we conducted molecular docking using the CDOCKER algorithm within the Discovery Studio 2024 (BIOVIA (Dassault Systèmes)). The structure of LY96 (or MD-2) was extracted from the human TLR4-MD-2 complex (PDB: 3FXI),^34^ and the binding site was defined based on the LPS-binding region of 3FXI. Analysis of the top 10 docking poses revealed consistent conformations, with the 3D docking pose showing compound **8** positioned within the hydrophobic pocket of LY96, surrounded by residues such as Ile80, Ile124, Cys133, and Ile153 (Figure 3e,f). The 2D interaction diagram further detailed van der Waals interactions with these residues, with distances ranging from 4.05 to 4.66 Å, while additional hydrophobic contacts with Ile52, Leu78, Phe126, and Phe151 contributed to stabilizing the ligand-protein complex (Figure 3g). For comparison, diosgenin (**2**) could also be accommodated within the LY96 binding pocket, however, it exhibited less favorable interaction than compound **8** (SI Figure S35), indicating weaker predicted binding affinity. To further strengthen the stability assessment of the compound **8**-LY96 complex, we complemented molecular dynamics (MD) simulations. The top-ranked pose of compound **8** bound to LY96 from the previous docking poses was subjected to MD simulation and compared with the apo LY96 protein. Analysis of the root-mean-square deviation (RMSD) trajectories revealed that LY96 in complex with compound **8** reached a stable conformational equilibrium with reduced fluctuation compared to the apo protein, indicating that ligand binding contributes to overall structural stabilization (SI Figure S36). In addition, residue-level root-mean-square fluctuation (RMSF) analysis showed attenuated flexibility in key regions surrounding the binding pocket upon ligand binding, particularly near residues Ile80, Ile124, Cys133, and Ile153, which were identified as major contributors in the docking model. These findings collectively support LY96 as a functional target of compound **8**, contributing to the attenuation of LPS-induced inflammatory responses through both direct binding and downstream signaling modulation. This mechanism aligns with the observed suppression of NF-κB and reinforces the role of neuroinflammation as a key contributor to depressive disorders.^21^

### Validation of Neuroprotective Effect

Given the promising antineuroinflammatory properties of compound **8**, we examined its ability to suppress LPS-induced microglial activation in BV-2 cells. By monitoring Iba1, a widely used marker of microglial activation, we confirmed that compound **8** clearly inhibited LPS-induced microglial activation in BV-2 cells (Figure 4a). These results prompted us to evaluate its neuroprotective effects on neurons using a coculture system of BV-2 microglial cells and dye-labeled HT22 neuronal cells, analyzed by flow cytometry to assess neuronal viability. BV-2 cells were stimulated with LPS, with or without compound **8**, for 4 h; dye-labeled HT22 cells were then added, and viable neuronal cells were analyzed after 20 h. Compound **8** showed minimal effects on cell viability in HT22 cells compared to the DMSO control, however, LPS stimulation significantly induced neuronal cell death (Figure 4b). Notably, the detrimental effect of LPS on HT22 cells was ameliorated in the presence of compound **8**, demonstrating its protective effect against LPS-induced neurotoxicity. Together, these results indicate that compound **8** protects neurons from inflammation-induced damage, likely through suppression of microglial activation rather than direct neuronal effects, and further support its therapeutic potential in neuroinflammation-driven neurological disorder.

**Figure 4:**
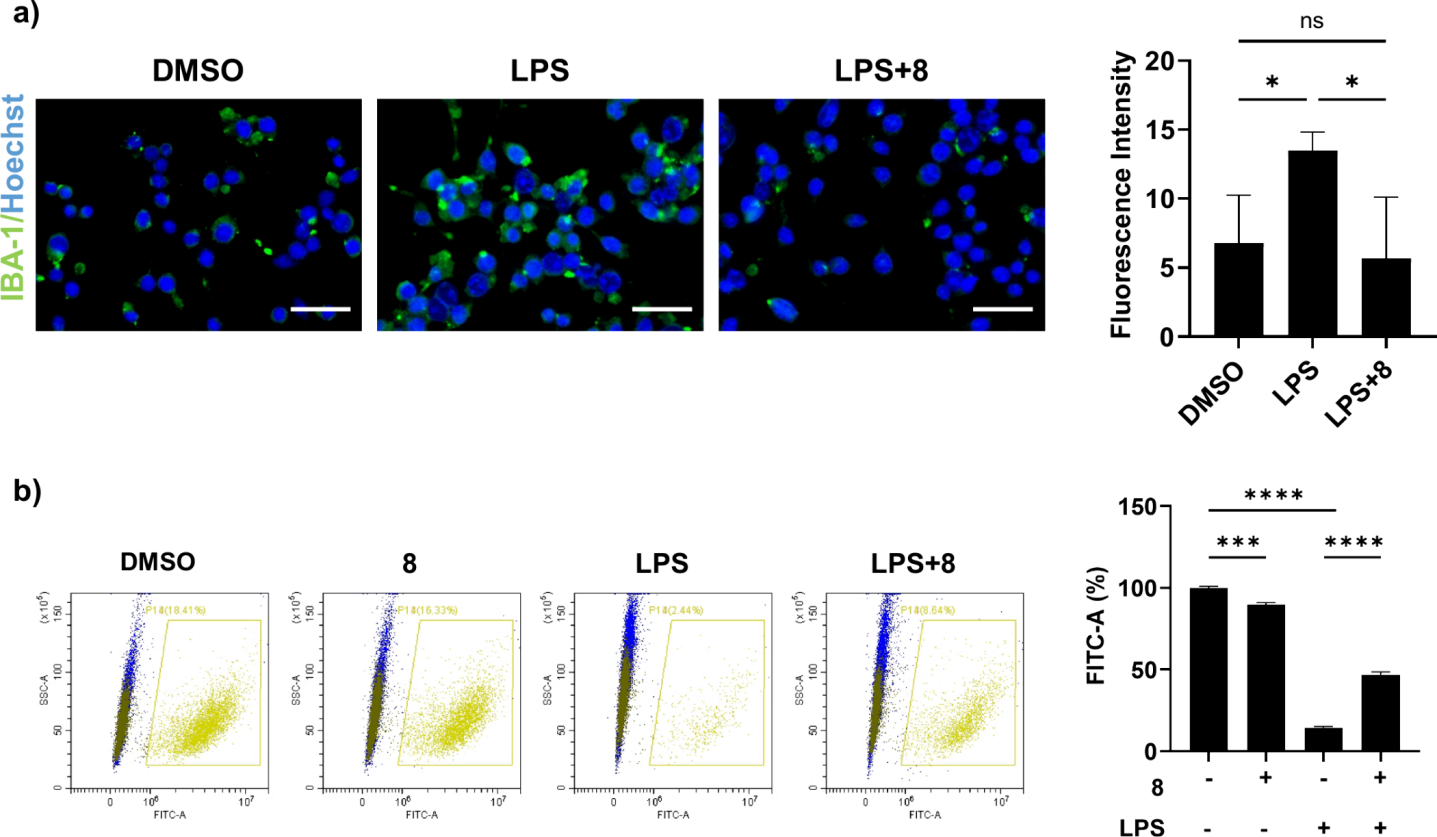
Coculture assay for evaluating neuroprotection. a) Immunofluorescent images (left) and quantification graph (right) of BV-2 cells. Cells were pretreated with compound **8** or DMSO for 1 h, and then treated with LPS (200 ng/mL) for 24 h. Green: anti-IBA 1, Blue: Hoechst. Scale bar is 50 μm. Quantification graph for green fluorescent intensity about approximately 100 cells from three experiments. b) FACS analysis of neuron-glia coculture assay (left) and quantified graph for dye-labeled HT22 cells (right). BV-2 cells were treated by LPS or compound **8,** then prelabeled HT22 cells using by cell tracker green dye were directly cocultured for 20 h. Viable HT22 cells were monitored by FACS. Unpaired *t* test for statistical analysis.

### Alleviation of LPS-Induced Reactive Astrocyte and Microglial Activation in the Hypothalamus

To determine whether systemic administration of compound **8** (50 mg/kg, i.p.) mitigates LPS (1 mg/kg, i.p.)-induced neuroinflammation, we pursued immunohistochemistry on hypothalamic tissue samples from treated mice. The hypothalamus was selected due to its central role in the hypothalamic-pituitary-adrenal (HPA) axis and its well-established involvement in stress regulation and depressive behaviors, as well as prior reports demonstrating robust LPS-induced neuroinflammatory response in this region.^21,30,31^ In LPS-treated mice, the intensity of glial fibrillary acidic protein (GFAP), an astrocytic marker, was significantly elevated in hypothalamic astrocytes compared to control mice, with astrocytes exhibiting severe hypertrophy and an increased number of branches (Figure 5a). Similarly, the intensity of Iba1 was considerably increased in the hypothalamic microglia of LPS-treated mice, accompanied by pronounced morphological changes (Figure 5b), including enlarged soma size and a retraction of ramified processes, consistent with an activated microglial phenotype. The adverse effects were dramatically rescued in the presence of compound **8** based on the intensities of GFAP and Iba1 in the hypothalamic astrocytes and microglia, respectively (Figure 5a and 5c). Furthermore, compound **8** partially normalized LPS-induced glial activation, with astrocyte and microglia morphology shifting toward a less activated state compared with the LPS-treated group (Figure 5b). Given the critical role of the hypothalamus in HPA axis regulation and the established link between sustained glial activation, pro-inflammatory cytokine release, and disrupted neuroplasticity in depression,^37,38^ the attenuation of reactive gliosis by compound **8** may contribute to improved brain function and amelioration of depressive-like behaviors observed *in vivo*. Together, these findings demonstrate that compound **8** effectively suppresses reactive astrogliosis and microgliosis in the hypothalamus, key hallmarks of neuroinflammation, and support its ability to modulate central neuroimmune activation *in vivo.*

**Figure 5:**
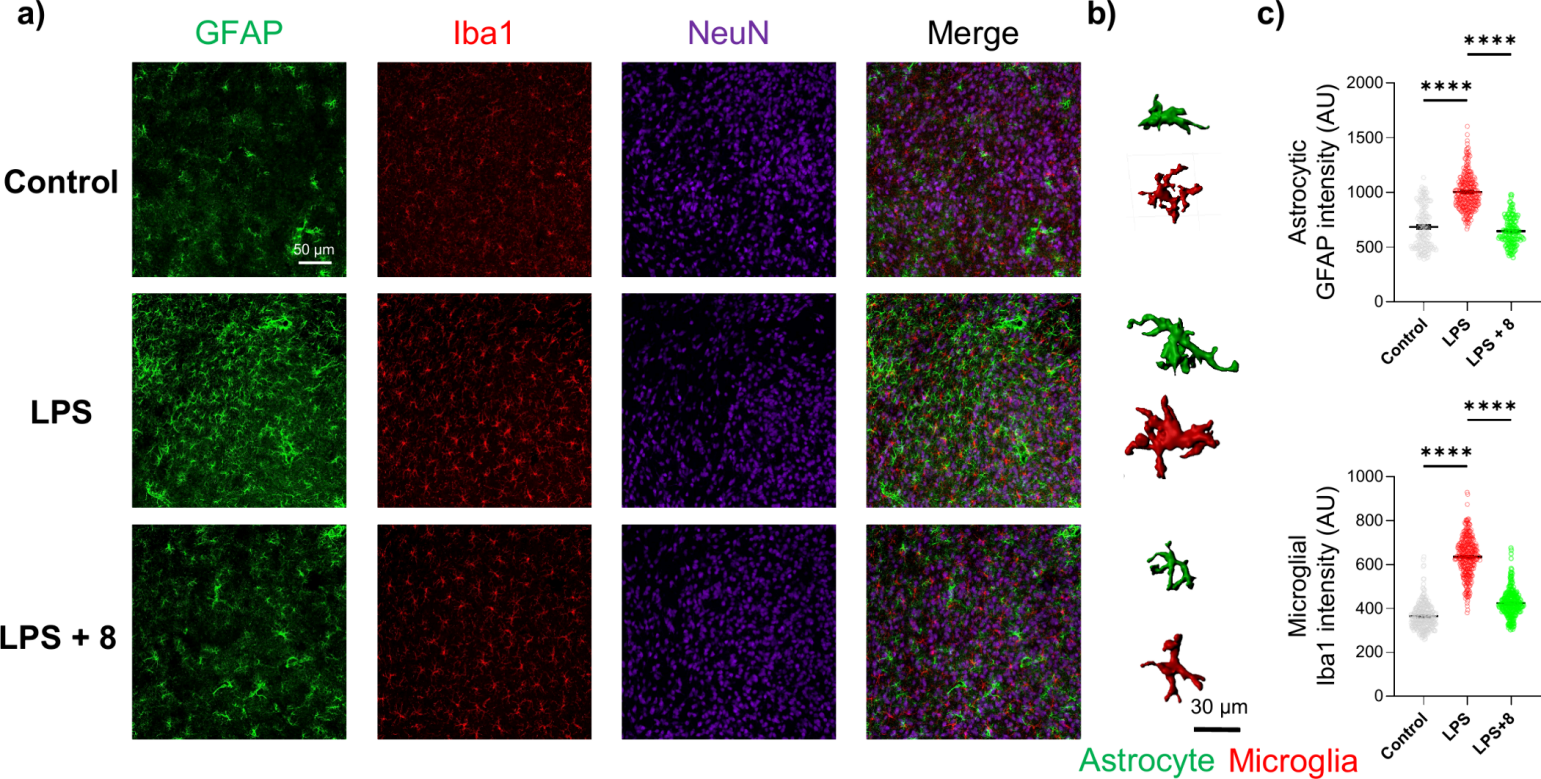
Alleviation of neuroinflammation in hypothalamus. a) Representative confocal images of hypothalamus tissues stained with GFAP, Iba, and NeuN. Mice were administrated by compound **8** (i.p. 50 mg/kg) or LPS (1 mg/kg), then sacrificed 3 days after LPS administration for immunohistochemistry. b) 3-D deconvolution images for morphology of astrocyte (green) and microglia (red). c) Quantification of the integrated density of GFAP-positive (GFAP^+^) astrocytes and Iba1-positive (Iba1^+^) microglia in the hypothalamus, respectively. One-way ANOVA for statistical analysis.

### Pharmacokinetic (PK) Profile of Compound **8**

The PK profile of compound **8** in ICR mice bolsters its therapeutic potential (Table 2). Intravenous (IV) administration (5 mg/kg) showed *C*_max_ of 664.23 ± 57.07 ng/mL (*T*_max_: 0.083 h), *T*_1/2_ of 24.15 ± 4.24 h, and AUC_0‑∞_ of 6989.95 ± 299.77 h·ng/mL, with a moderate clearance (Cl: 11.94 ± 0.51 mL/min/kg) and large volume of distribution at steady state (V_ss_: 23521.53 ± 5081.52 mL/kg), suggesting extensive tissue penetration. Intraperitoneal (IP) dosing (20 mg/kg) increased *C*_max_ of 1574.40 ± 277.32 ng/mL (*T*_max_: 0.5 h) and AUC_0‑∞_ of 25427.08 ± 2684.41 h·ng/mL, with a bioavailability (F) of 122.36% and shorter *T*_1/2_ of 12.83 ± 1.35 h, indicating efficient absorption. Oral (PO) administration (20 mg/kg) yielded *C*_max_ of 926.93 ± 93.28 ng/mL (*T*_max_: 2 h), AUC_0‑∞_ of 30293.26 ± 4842.85 h·ng/mL, and F of 101.96%, with *T*_1/2_ of 24.25 ± 3.21 h. Apparent bioavailability values exceeding 100% have been reported for compounds exhibiting nonlinear pharmacokinetics and may arise from factors such as saturable tissue distribution or metabolism, prolonged absorption from a peritoneal depot. To further assess metabolic stability, compound **8** was evaluated in mouse plasma, where it exhibited good stability with a calculated half-life of 231 min (SI Figure S34). This result is consistent with the prolonged systemic half-life observed *in vivo*. Collectively, the high bioavailability observed via both IP and PO routes, together with sustained exposure and extensive tissue distribution, overcomes key pharmacokinetic limitations of diosgenin and supports the *in vivo* efficacy of compound **8.**

**Table 2:** *In vivo* Pharmacokinetic Parameters of Compound **8** in ICR Mice^a^

plasma	intravenous administration	intraperitoneal administration	oral administration
*T*_max_ (h)	0.083	0.50	2.00
*C*_max_ (ng/mL)	664.23 ± 57.07	1574.40 ± 277.32	926.93 ± 93.28
*T*_1/2_ (h)	24.15 ± 4.24	12.83 ± 1.35	24.25 ± 3.21
AUC_last_ (h·ng/mL)	3687.81 ± 495.42	18,049.52 ± 876.56	15,040.05 ± 1352.83
AUC_0‑∞_ (h·ng/mL)	6989.95 ± 297.77	25,427.08 ± 2684.41	30,293.26 ± 4842.85
Cl (mL/min/kg)	11.94 ± 0.51		
MRT_inf__obs (h)	32.70 ± 5.70	18.37 ± 2.21	34.59 ± 4.11
V_ss_ (mL/kg)	23521.53 ± 5081.52		
F (%)		122.36	101.96

a Mean (± SD) pharmacokinetic parameters after intravenous injection at a dose of 5 mg/kg (n = 3), intraperitoneal injection at a dose of 20 mg/kg (n = 3) and oral administration at a dose of 20 mg/kg (n = 3) of compound **8** to ICR mice. *T*_max_, time to reach *C*_max_; *C*_max_, peak plasma concentration; *T*_1/2,_ terminal half-life; AUC_last_, total area under the plasma concentration–time curve from time zero to last measured time; AUC_0‑∞_, total area under the plasma concentration–time curve from time zero to time infinity; Cl, time-averaged total body clearance; MRT, mean residence time; V_ss_, state volume of distribution; F, oral bioavailability; SD, standard deviations.

### Evaluation of Antidepressant Effect through Animal Behavior Test

Building on the significant antineuroinflammatory effects of compound **8** observed *in vitro* and *ex vivo* analysis, we investigated its potential antidepressant properties *in vivo*, given the well-established link between endotoxin-induced neuroinflammation and acute depressive behaviors in mice. Using 5-week-old ICR mice, we performed three behavioral tests, namely, tail suspension test (TST), forced swimming test (FST), and sucrose preference test (SPT), to evaluate antidepressant activity related to despair and anhedonia (Figure 6a). To ensure sufficient brain exposure and to enable detection of behavioral phenotypic changes driven by antineuroinflammatory effects in the CNS, compound **8** was administered at a dose of 50 mg/kg. In LPS-treated mice, we observed significantly prolonged immobility periods in both TST and FST compared to controls, indicative of depressive-like behavior. Treatment with compound **8** significantly attenuated this adverse effect, reducing immobility times toward control levels (Figure 6b), consistent with previous reports showing that modulation of inflammatory signaling preferentially alleviates despair-related behaviors.^22,23^ Moreover, LPS-induced mice exhibited reduced sucrose preference in the SPT compared to controls. Although this reduction was not statistically significant (P = 0.1552), compound **8** partially alleviated the loss of preference (Figure 6b). This differential behavioral profile suggests that compound **8** may preferentially target specific neurobiological pathways associated with despair-related behaviors, while other symptom domains may involve distinct mechanisms. Collectively, these behavioral findings support the therapeutic potential of compound **8** as an antidepressant candidate in animal models.

**Figure 6:**
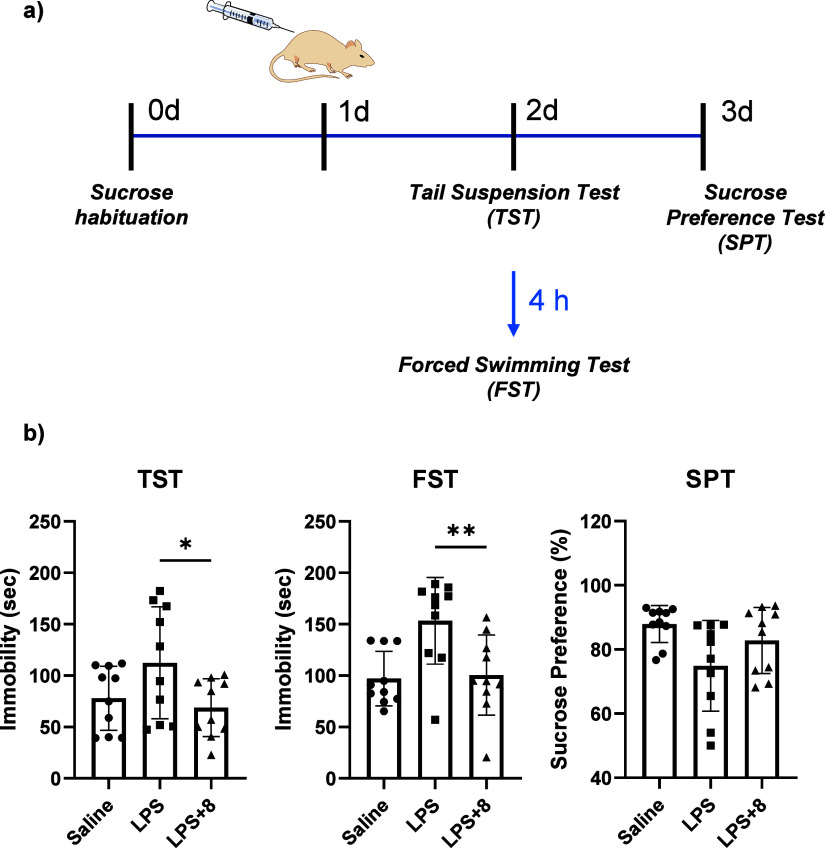
Mouse behavior test for depression. a) Experimental workflow for antidepression effect on mouse model using three different behavior tests; tail suspension test (TST), forced swimming test (FST), and sucrose preference test (SPT). After sucrose habituation on day 1 before compound injection, compound **8** (50 mg/kg) was administrated by i.p, then LPS (1 mg/kg) was treated by i.v after 1 h on day 2. TST and FST were performed on day 3 and SPT was performed during a day and sucrose consumption was observed on day 4. b) Behavior tests for depressive-like behavior. Immobility time was measured in TST and FST. Sucrose preference percentage was measured in SPT. *N* = 10. Mean + SEM. Unpaired *t* test for statistical analysis.

## Conclusions

Depression, a prevalent and growing global mental illness, is inadequately addressed by current antidepressants like TCAs and SSRIs, which are limited by side effects and incomplete efficacy. The etiology of depression remains controversial, but neuroinflammation has emerged as an important factor, positioning anti-inflammatory agents as promising therapeutic candidates. In this study, we report the discovery of compound **8**, an *N*,*N*-dimethylamino diosgenin derivative that effectively overcomes the cytotoxicity and pharmacokinetic shortcomings of the parent natural products dioscin and diosgenin. With a TI value of 19.8 in LPS-stimulated BV-2 microglial cells, compound **8** potently suppresses NO production and pro-inflammatory markers and upregulates the anti-inflammatory gene while exhibiting minimal cytotoxicity. Mechanistic studies revealed direct binding to LY96 (MD-2) as a target, disrupting LPS-TRL4 complex formation and downstream NF-κB activation. This targeted interference translated to robust neuroprotection *in vitro*, attenuation of hypothalamic gliosis *in vivo*, and significant reversal of LPS-induced depressive-like behaviors, particularly despair phenotypes, in mice. These effects were supported by a favorable PK profile, featuring high bioavailability and CNS penetration. Collectively, our findings suggest that our compound **8** holds significant therapeutic potential, reinforcing the alleviation of neuroinflammation as a promising strategy for developing next-generation antidepressants.

## Experimental Section

### Chemistry

All reactions were carried out under dry nitrogen unless otherwise indicated. Commercially available reagents were used without further purification. Solvents and gases were dried according to standard procedures. Organic solvents were evaporated with reduced pressure using a rotary evaporator. Analytical thin-layer chromatography (TLC) was performed using glass/aluminum plates, silica gel 60 coated (0.25 mm, Merck) with fluorescent indicator F254. TLC plates were visualized by UV light (254 nm) and then were visualized with a KMnO_4_, ninhydrin, or p-anisaldehyde staining solution followed by brief heating on a hot plate. Flash column chromatography was performed using silica gel 60 (230–400 mesh, Merck) with the indicated solvents. ^1^H and ^13^C NMR spectra were acquired on a Bruker AVANCE 400 or Agilent DD2 600 MHz spectrometer, housed in the NMR Facility of Korea Institute of Science and Technology. Data are reported as (s = singlet, d = doublet, t = triplet, q = quartet, m = multiplet, br = broad; coupling constant(s) in Hz, integration). The Mass analyses were carried out using a Bruker Daltonik micro TOF-Q II ESI at the Mass Spectrometry Lab of the Department of Chemistry at Sogang University. The purity of final compounds was determined by HPLC, with all compounds achieving a minimum purity of 95%. HPLC was performed on an Agilent 1100 system with an Agilent EC-C18 column (4.6 × 150 mm, 3.5 μm).

#### (4S,5′R,6aR,6bS,8aS,8bR,9S,10R,11aS,12aS,12bS)-5′,6a,8a,9-Tetramethyl-1,3,3′,4,4’,5,5′,6,6a,6b,6’,7,8,8a,8b,9,11a,12,12a,12b-icosahydrospiro­[naphtho­[2’,1’:4,5]­indeno­[2,1-*b*]­furan-10,2’-pyran]-4-yl 4-Methylbenzenesulfonate (**3**)

p-Toluenesulfonyl chloride (1.05 g, 5.55 mmol), anhydrous pyridine (0.95 mL, 11.82 mmol), and a catalytic amount of DMAP (15 mg, 0.121 mmol) were sequentially added to a solution of diosgenin (0.5 g, 1.21 mmol) in anhydrous dichloromethane (DCM, 7 mL). The resulting mixture was stirred at 0 °C for 2 h and then warmed to room temperature over 24 h under argon environment. The reaction mixture was washed with a 5% solution of HCl (5 mL), and with a saturated solution of NaHCO_3_ (5 mL). The organic layer was dried over anhydrous MgSO_4_, filtered and concentrated under reduced pressure. Since the crude exhibited decomposition during flash column chromatography, the residue was used without further purification.

#### (4R,5′R,6aR,6bS,8aS,8bR,9S,10R,11aS,12aS,12bS)-5′,6a,8a,9-Tetramethyl-1,3,3′,4,4’,5,5′,6,6a,6b,6’,7,8,8a,8b,9,11a,12,12a,12b-icosahydrospiro­[naphtho­[2’,1’:4,5]­indeno­[2,1-*b*]­furan-10,2’-pyran]-4-ol (**4**)

Sodium hydroxide (97 mg, 2.42 mmol) was added to a solution of crude residue in DMF (5 mL). The reaction mixture was stirred at 130 °C for 12 h, at which time the reaction mixture was cooled down to room temperature, and diluted with ethyl acetate (20 mL). The mixture was washed with brine (20 mL) and water (20 mL), dried over anhydrous sodium sulfate, and concentrated under reduced pressure. The residue was subjected to flash column chromatograph (EtOAc/hexanes = 1/4) to afford the alcohol **4** (0.31 g, 0.75 mmol, 62%) as a white solid. ^1^H NMR (600 MHz, CDCl_3_) δ (ppm) 5.34 (dt, 1H, *J* = 4.8, 2.0 Hz), 4.44–4.36 (m, 1H), 3.57–3.41 (m, 2H), 3.36 (t, 1H, *J* = 10.9 Hz), 2.34–2.17 (m, 2H), 1.98 (ddt, 2H, *J* = 17.5, 7.6, 3.9 Hz), 1.91–1.05 (m, 23H), 1.02 (s, 3H), 0.96 (d, 3H, *J* = 6.9 Hz), 0.78 (t, 6H, *J* = 3.2 Hz). ^13^C NMR (150 MHz, CDCl_3_) δ (ppm) 140.94, 121.54, 109.42, 80.95, 71.84, 66.97, 62.21, 56.64, 50.18, 42.40, 41.73, 40.39, 39.91, 37.35, 36.77, 32.18, 31.97, 31.74, 31.56, 31.51, 30.42, 28.92, 21.00, 19.55, 17.27, 16.42, 14.66. HPLC purity: 95.9%.

#### (4S,5′R,6aR,6bS,8aS,8bR,9S,10R,11aS,12aS,12bS)-4-Methoxy-5′,6a,8a,9-tetramethyl-1,3,3′,4,4’,5,5′,6,6a,6b,6’,7,8,8a,8b,9,11a,12,12a,12b-icosahydrospiro­[naphtho­[2’,1’:4,5]­indeno­[2,1-*b*]­furan-10,2’-pyran] (**5**)

Sodium hydride (19.29 mg, 0.482 mmol) was added to a solution of diosgenin (100 mg, 0.241 mmol) in anhydrous THF (5 mL) at 0 °C. The reaction mixture was stirred at 0 °C for 30 min, at which time iodomethane (0.030 mL, 0.482 mmol) was added. The reaction mixture was stirred at 60 °C for an additional 18 h under argon atmosphere. The reaction mixture was cooled down to room temperature, quenched with water, partitioned with EtOAc (10 mL × 2), dried over anhydrous sodium sulfate, concentrated, and purified by flash column chromatography (EtOAc/hexanes = 1/16) to afford the methyl ether **5** (97.4 mg, 0.23 mmol, 94%). ^1^H NMR (400 MHz, CDCl_3_) δ (ppm) 5.35 (dt, 1H, *J* = 5.4, 2.0 Hz), 4.40 (ddd, 1H, *J* = 8.7, 7.5, 6.3 Hz), 3.47 (ddd, 1H, *J* = 10.9, 4.5, 2.0 Hz), 3.38 (d, 1H, *J* = 10.9 Hz), 3.35 (s, 3H), 3.05 (tt, 1H, *J* = 11.3, 4.5 Hz), 2.39 (ddd, 1H, *J* = 13.2, 4.7, 2.3 Hz), 2.15 (ddd, 1H, *J* = 15.4, 10.7, 2.7 Hz). 2.06–1.05 (m, 22H), 1.01 (s, 3H), 0.97 (d, 3H, *J* = 7.0 Hz), 0.78 (t, 6H, *J* = 3.2 Hz). ^13^C NMR (100 MHz, CDCl_3_) δ (ppm) 141.07, 121.44, 109.42, 80.97, 80.43, 66.99, 62.26, 56.69, 55.75, 50.28, 41.75, 40.42, 39.95, 38.81, 37.30, 37.18, 32.24, 32.00, 31.59, 31.54, 30.45, 28.95, 28.16, 21.01, 19.54, 17.28, 16.43, 14.67. HRMS (ESI, positive) *m*/*z* for C_28_H_44_O_3_ [M + H]^+^: calc.429.3363, found 429.3366. HPLC purity: 97.3%.

#### (4R,5′R,6aR,6bS,8aS,8bR,9S,10R,11aS,12aS,12bS)-4-Azido-5′,6a,8a,9-tetramethyl-1,3,3′,4,4’,5,5′,6,6a,6b,6’,7,8,8a,8b,9,11a,12,12a,12b-icosahydrospiro­[naphtho­[2’,1’:4,5]­indeno­[2,1-*b*]­furan-10,2’-pyran] (**6**)

Sodium azide (0.878 g, 13.51 mmol) was added to a solution of crude residue **3** (diosgenin 0.83 g, 2.02 mmol) in anhydrous DMF (20 mL). The reaction mixture was stirred at 80 °C for 22 h under argon atmosphere. The reaction mixture was cooled down to room temperature, diluted with EtOAc (30 mL), washed with water (20 mL) and brine (20 mL), dried over anhydrous MgSO_4_, concentrated, and purified by flash column chromatography (EtOAc/hexanes = 1/50) to afford the azide **6** (0.61 g, 1.39 mmol, 69%). ^1^H NMR (600 MHz, CDCl_3_) δ (ppm) 5.39 (d, 1H, *J* = 5.0 Hz), 4.41 (q, 1H, *J* = 7.5 Hz), 3.87 (p, 1H, *J* = 3.1 Hz), 3.47 (ddd, 1H, *J* = 11.1, 4.4, 2.1 Hz), 3.37 (t, 1H, *J* = 11.0 Hz), 2.51 (dt, 1H, *J* = 14.9, 2.9 Hz), 2.19 (dt, 1H, *J* = 15.0, 2.6 Hz), 2.05–1.05 (m, 25H), 1.02 (s, 3H), 0.97 (d, 3H, *J* = 7.0 Hz), 0.78 (t, 6H, *J* = 3.2 Hz). ^13^C NMR (150 MHz, CDCl_3_) δ (ppm) 138.26, 123.03, 109.39, 80.95, 66.97, 62.21, 58.30, 56.60, 49.97, 41.75, 40.37, 39.87, 37.35, 36.19, 33.73, 32.11, 31.95, 31.53, 31.45, 30.45, 28.96, 26.23, 20.65, 19.16, 17.28, 16.42, 14.66. HPLC purity: 95.7%.

#### (4R,5′R,6aR,6bS,8aS,8bR,9S,10R,11aS,12aS,12bS)-5′,6a,8a,9-Tetramethyl-1,3,3′,4,4’,5,5′,6,6a,6b,6’,7,8,8a,8b,9,11a,12,12a,12b-icosahydrospiro­[naphtho­[2’,1’:4,5]­indeno­[2,1-*b*]­furan-10,2’-pyran]-4-amine (**7**)

Triphenylphosphine (0.986 g, 3.76 mmol) was added to a solution of the azide **6** (0.826 g, 1.879 mmol) in THF/H_2_O (11 mL, 10/1). The reaction mixture was stirred at 60 °C for 6 h, and then was cooled down to room temperature. The mixture was partitioned with EtOAc (10 mL × 3), washed with brine (10 mL), dried over anhydrous sodium sulfate, and concentrated under reduced pressure. The residue was subjected to flash column chromatography (DCM/MeOH = 50/1) to afford the amine **7** (0.662 g, 1.60 mmol, 85%). ^1^H NMR (600 MHz, CDCl_3_) δ (ppm) 5.35 (s, 1H), 4.39 (q, 1H, *J* = 7.5 Hz), 3.45 (d, 1H, *J* = 10.9 Hz), 3.36 (td, 1H, *J* = 11.0, 3.2 Hz), 3.17 (s, 1H), 2.56 (d, 1H, *J* = 14.0 Hz), 2.05–1.03 (m, 34H), 1.00 (d, 3H, *J* = 3.2 Hz), 0.97–0.93 (m, 3H), 0.77 (d, 6H, *J* = 4.1 Hz). ^13^C NMR (150 MHz, CDCl_3_) δ (ppm) 138.45, 123.61, 109.31, 80.89, 66.89, 62.21, 56.51, 50.20, 47.19, 41.69, 40.31, 39.83, 39.37, 37.61, 33.03, 32.21, 31.92, 31.49, 30.38, 29.79, 28.90, 28.84, 20.65, 18.95, 17.23, 16.35, 14.63. HRMS (ESI, positive) *m*/*z* for C_27_H_43_NO_2_ [M + H]^+^: calc. 414.3367, found 414.3367.

#### (4R,5′R,6aR,6bS,8aS,8bR,9S,10R,11aS,12aS,12bS)-N,N,5′,6a,8a,9-Hexamethyl-1,3,3′,4,4’,5,5′,6,6a,6b,6’,7,8,8a,8b,9,11a,12,12a,12b-icosahydrospiro­[naphtho­[2’,1’:4,5]­indeno­[2,1-*b*]­furan-10,2’-pyran]-4-amine (**8**)

To a solution of amine **7** (11 mg, 0.027 mmol) in MeOH (3 mL) were added 37% aqueous formaldehyde (0.24 mL, 3.19 mmol) and formic acid (7.7 μL, 0.205 mmol), sequentially.^36^ The reaction mixture was stirred for 30 min, at which time sodium cyanoborohydride (10 mg, 0.16 mmol) was added in three portions over 3 h and was stirred for an additional 4 h. The reaction mixture was quenched with a saturated solution of NaHCO_3_ (3 mL), partitioned with EtOAc (5 mL × 3). The combined organic layer was washed with brine (5 mL), dried over anhydrous sodium sulfate, concentrated under reduced pressure, and purified by flash column chromatography (DCM/MeOH = 15/1 with 3% triethylamine) to afford the dimethylamino derivative **8** (10.3 mg, 0.023 mmol, 88%). ^1^H NMR (400 MHz, CDCl_3_) δ (ppm) 5.32 (apparent d, 1H, *J* = 4.9 Hz), 4.36 (td, 1H, *J* = 8.0, 6.3 Hz), 3.43 (ddd, 1H, *J* = 10.9, 4.4, 2.0 Hz), 3.33 (t, 1H, *J* = 10.9 Hz), 2.44–2.39 (m, 1H), 2.36 (s, 6H), 2.32 (s, 1H), 2.03–1.07 (m, 26H), 1.01 (s, 3H), 0.93 (d, 3H, *J* = 6.9 Hz), 0.75 (t, 6H, *J* = 3.1 Hz). ^13^C NMR (100 MHz, CDCl_3_) δ (ppm) 138.93, 122.41, 109.28, 80.91, 66.87, 63.02, 62.11, 56.40, 49.00, 43.23, 41.67, 40.32, 39.76, 37.23, 34.90, 33.37, 31.86, 31.83, 31.46, 31.42, 30.36, 28.89, 24.53, 20.61, 19.86, 17.21, 16.34, 14.59. HRMS (ESI, positive) *m*/*z* for C_29_H_47_NO_2_ [M + H]^+^: calc. 442.3680, found 442.3682. HPLC purity: 100%.

#### (4R,5′R,6aR,6bS,8aS,8bR,9S,10R,11aS,12aS,12bS)-N,N,N,5′,6a,8a,9-Heptamethyl-1,3,3′,4,4’,5,5′,6,6a,6b,6’,7,8,8a,8b,9,11a,12,12a,12b-icosahydrospiro­[naphtho­[2’,1’:4,5]­indeno­[2,1-*b*]­furan-10,2’-pyran]-4-aminium (**9**)

Iodomethane (0.05 mL, 0.73 mmol) and potassium carbonate (50 mg, 0.36 mmol) were added to a solution of amine **7** (30 mg, 0.073 mmol) in anhydrous DCM/acetonitrile (1 mL, v/v = 1/1). The reaction mixture was refluxed at 60 °C for 12 h under argon atmosphere. The mixture was filtered on Celite, and the filtrate was concentrated under reduced pressure. The residue was subjected to flash column chromatography (DCM/MeOH = 9/1) to afford the permethylated amine compound **9** (32 mg, 0.071 mmol, 97%). ^1^H NMR (400 MHz, CD_3_OD) δ (ppm) 5.71 (dt, 1H, *J* = 4.5, 2.1 Hz), 4.51–4.41 (m, 1H), 3.79 (tdd, 1H, *J* = 8.3, 5.7, 2.0 Hz), 3.50 (ddd, 1H, *J* = 10.9, 4.4, 2.0 Hz), 2.90–2.79 (m, 1H), 2.66 (d, 1H, *J* = 16.6 Hz), 2.21–1.20 (m, 25H), 1.11 (s, 3H), 1.03 (d, 3H, *J* = 7.0 Hz), 0.89 (s, 3H), 0.85 (d, 3H, *J* = 6.4 Hz). ^13^C (100 MHz, CD_3_OD) δ (ppm) 139.45, 126.29, 110.52, 82.18, 72.93, 67.83, 63.68, 57.82, 52.35, 52.32, 52.27, 47.83, 42.86, 41.58, 40.82, 37.91, 32.74, 32.69, 32.59, 32.42, 31.41, 30.65, 29.87, 22.13, 22.09, 17.51, 16.91, 14.91. HRMS (ESI, positive) *m*/*z* for C_30_H_50_NO_2_ [M]^+^: calc. 456.3836, found 456.3838. HPLC purity: 95.8%.

#### *tert*-Butyl-((4R,5′R,6aR,6bS,8aS,8bR,9S,10R,11aS,12aS,12bS)-5′,6a,8a,9-Tetramethyl-1,3,3′,4,4’,5,5′,6,6a,6b,6’,7,8,8a,8b,9,11a,12,12a,12b-icosahydrospiro­[naphtho­[2’,1’:4,5]­indeno­[2,1-*b*]­furan-10,2’-pyran]-4-yl)­carbamate (**10**)

Triethylamine (0.09 mL, 0.67 mmol) and di*tert*-butyl dicarbonate (0.15 mg, 0.67 mmol) were added to a solution of amine **7** (261 mg, 0.61 mmol) in anhydrous DCM (5 mL). The reaction mixture was stirred at room temperature for 12 h under argon atmosphere. The mixture was diluted with DCM (5 mL), washed with water (5 mL), dried over anhydrous sodium sulfate, concentrated under reduced pressure, and purified by flash column chromatography (EtOAc/hexanes = 1/16) to afford the compound **10** (313 mg, 0.61 mmol, quant.). ^1^H NMR (400 MHz, CDCl_3_) δ (ppm) 5.34 (d, 1H, *J* = 5.1 Hz), 4.60 (d, 1H, *J* = 7.9 Hz), 4.44–4.34 (m, 1H), 3.87–3.80 (m, 1H), 3.45 (ddd, 1H, *J* = 11.0, 4.5, 2.0 Hz), 3.35 (t, 1H, *J* = 10.9 Hz), 2.54 (d, 1H, *J* = 14.7 Hz), 2.04–1.46 (m, 18H), 1.42 (s, 9H), 1.35–1.03 (m, 6H), 1.00 (s, 3H), 0.95 (d, 3H, *J* = 6.9 Hz), 0.77 (t, 6H, *J* = 3.2 Hz). ^13^C NMR (100 MHz, CDCl_3_) δ (ppm) 155.18, 139.05, 123.12, 109.37, 80.89, 79.06, 66.94, 62.21, 56.64, 50.36, 46.72, 41.71, 40.34, 39.88, 37.53, 37.43, 34.00, 32.16, 31.92, 31.49, 31.44, 30.40, 28.91, 28.57, 26.49, 20.65, 18.99, 17.25, 16.37, 14.63.

#### (4R,5′R,6aR,6bS,8aS,8bR,9S,10R,11aS,12aS,12bS)-N,5′,6a,8a,9-Pentamethyl-1,3,3′,4,4’,5,5′,6,6a,6b,6’,7,8,8a,8b,9,11a,12,12a,12b-icosahydrospiro­[naphtho­[2’,1’:4,5]­indeno­[2,1-*b*]­furan-10,2’-pyran]-4-amine (**11**)

Lithium aluminum hydride (LAH, 42 mg, 1.11 mmol) was added to a solution of compound **10** (285 mg, 0.56 mmol) in anhydrous THF (7 mL) at 0 °C. The reaction mixture was refluxed for 4 h.^36^ The reaction mixture was quenched by following Fieser method. The organic layer was dried over anhydrous sodium sulfate, concentrated under reduced pressure, and purified by flash column chromatography (DCM/MeOH = 9/1) to afford the monomethylamine **11** (200 mg, 0.47 mmol, 84%). ^1^H NMR (400 MHz, CDCl_3_) δ (ppm) 5.34 (dd, 1H, *J* = 4.4, 2.6 Hz), 4.39 (td, 1H, *J* = 7.9, 6.4 Hz), 3.46 (ddd, 1H, *J* = 10.9, 4.4, 2.0 Hz), 3.36 (t, 1H, *J* = 10.9 Hz), 2.78 (t, 1H, *J* = 3.3 Hz), 2.47 (dq, 1H, *J* = 14.4, 3.0 Hz), 2.36 (s, 3H), 2.19–1.06 (m, 29H), 1.02 (s, 3H), 0.96 (d, 3H, *J* = 7.0 Hz), 0.77 (t, 6H, *J* = 3.2 Hz). ^13^C NMR (100 MHz, CDCl_3_) δ (ppm) 139.30, 122.75, 109.38, 80.95, 66.96, 62.22, 56.63, 54.89, 50.23, 41.74, 40.37, 39.90, 37.55, 36.70, 33.49, 33.22, 32.15, 31.95, 31.53, 30.44, 29.83, 28.95, 25.19, 20.69, 19.26, 17.27, 16.40, 14.65. HRMS (ESI, positive) *m*/*z* for C_28_H_46_NO_2_ [M + H]^+^: calc. 428.3523, found 428.3525. HPLC purity: 100%.

#### (4R,5′R,6aR,6bS,8aS,8bR,9S,10R,11aS,12aS,12bS)-4-Bromo-5′,6a,8a,9-tetramethyl-1,3,3′,4,4’,5,5′,6,6a,6b,6’,7,8,8a,8b,9,11a,12,12a,12b-icosahydrospiro­[naphtho­[2’,1’:4,5]­indeno­[2,1-*b*]­furan-10,2’-pyran] (**12**)

Carbon tetrabromide (1.20 g, 3.62 mmol) and triphenylphosphine (0.95 g, 3.62 mmol) were added to a solution of diosgenin **2** (1 g, 2.41 mmol) in anhydrous DCM (20 mL) at 0 °C under argon environment. The reaction mixture was warmed to room temperature and stirred for an additional 4 h. The mixture was washed with brine (10 mL) and water (10 mL), dried over anhydrous MgSO_4_, and concentrated under reduced pressure. The residue was subjected to flash column chromatography (EtOAc/hexanes = 1/8) to afford the bromo derivative **12** (1.03 g, 90%). ^1^H NMR (600 MHz, CDCl_3_) δ (ppm) 5.36 (dt, 1H, *J* = 4.7, 2.0 Hz), 4.40 (q, 1H, *J* = 7.5 Hz), 3.91 (tt, 1H, *J* = 12.3, 4.4 Hz), 3.47 (ddd, 1H, *J* = 10.9, 4.4, 2.3 Hz), 3.37 (t, 1H, *J* = 11.0 Hz), 2.74 (tq, 1H, *J* = 12.8, 2.7 Hz), 2.59 (ddd, 1H, *J* = 13.6, 4.7, 2.3 Hz), 2.21–1.07 (m, 25H), 1.05 (s, 3H), 0.97 (d, 3H, *J* = 7.0 Hz), 0.79 (d, 6H, *J* = 6.3 Hz). ^13^C NMR (150 MHz, CDCl_3_) δ (ppm) 141.72, 122.19, 109.44, 80.91, 67.00, 62.21, 56.58, 52.56, 50.22, 44.38, 41.75, 40.43, 40.39, 39.83, 36.67, 34.45, 32.08, 31.96, 31.52, 31.40, 30.44, 28.94, 20.83, 19.41, 17.28, 16.42, 14.67. HPLC purity: 98.4%.

#### S-((4S,5′R,6aR,6bS,8aS,8bR,9S,10R,11aS,12aS,12bS)-5′,6a,8a,9-Tetramethyl-1,3,3′,4,4’,5,5′,6,6a,6b,6’,7,8,8a,8b,9,11a,12,12a,12b-icosahydrospiro­[naphtho­[2’,1’:4,5]­indeno­[2,1-*b*]­furan-10,2’-pyran]-4-yl) Ethanethioate (**13**)

Potassium thioacetate (0.22 g, 1.89 mmol) and a catalytic amount of 18-crown-6 (0.02 g, 0.06 mmol) were added to a solution of compound **12** (0.3 g, 0.63 mmol) in anhydrous DMF (6 mL). The reaction mixture was stirred at 100 °C for 24 h under argon atmosphere. The reaction mixture was cooled down to room temperature, diluted with EtOAc (12 mL), washed with brine (10 mL) and water (10 mL), dried over anhydrous sodium sulfate, concentrated under reduced pressure, and purified by flash column chromatography (EtOAc/hexanes = 1/30) to afford the compound **13** (200 mg, 67%). ^1^H NMR (600 MHz, CDCl_3_) δ (ppm) 5.29 (d, 1H, *J* = 5.1 Hz), 4.39 (q, 1H, *J* = 7.4 Hz), 3.97 (d, 1H, *J* = 4.3 Hz), 3.49–3.41 (m, 1H), 3.35 (t, 1H, *J* = 10.9 Hz), 2.74 (dq, 1H, *J* = 14.7, 2.5 Hz), 2.26 (s, 3H), 2.09–1.02 (m, 26H), 1.00 (s, 3H), 0.95 (d, 3H, *J* = 6.9 Hz), 0.77 (d, 6H, *J* = 5.4 Hz). ^13^C NMR (150 MHz, CDCl_3_) δ (ppm) 195.92, 139.25, 122.37, 109.35, 80.89, 66.92, 62.17, 56.59, 50.17, 43.24, 41.69, 40.31, 39.85, 37.74, 37.40, 35.60, 31.99, 31.90, 31.47, 31.33, 31.07, 30.39, 28.90, 27.62, 20.62, 19.28, 17.25, 16.38, 14.64.

#### (4S,5′R,6aR,6bS,8aS,8bR,9S,10R,11aS,12aS,12bS)-5′,6a,8a,9-Tetramethyl-1,3,3′,4,4’,5,5′,6,6a,6b,6’,7,8,8a,8b,9,11a,12,12a,12b-icosahydrospiro­[naphtho­[2’,1’:4,5]­indeno­[2,1-*b*]­furan-10,2’-pyran]-4-thiol (**14**)

1 M solution of sodium methoxide in MeOH (1.48 mL) was added to a solution of compound **13** (0.35 g, 0.74 mmol) in MeOH (7 mL). The reaction mixture was stirred at room temperature for 12 h. The mixture was quenched with 0.1 M solution of HCl (10 mL), partitioned with DCM (10 mL × 2), washed with brine (10 mL), dried over anhydrous MgSO_4_, concentrated under reduced pressure. The residue was subjected to flash column chromatography (EtOAc/hexanes = 1/20) to afford the thiol derivative **14** (0.26 g, 80%). ^1^H NMR (600 MHz, CDCl_3_) δ (ppm) 5.37 (dt, 1H, *J* = 5.8, 1.8 Hz), 4.42 (ddd, 1H, *J* = 8.6, 7.5, 6.3 Hz), 3.47 (ddd, 1H, *J* = 10.9, 4.4, 2.1 Hz), 3.44–3.33 (m, 2H), 2.81 (ddt, 1H, *J* = 12.4, 5.0, 2.5 Hz), 2.09–1.04 (m, 37H), 1.02–0.95 (m, 6H), 0.79 (t, 6H, *J* = 3.2 Hz). ^13^C NMR (150 MHz, CDCl_3_) δ (ppm) 138.17, 123.82, 109.43, 80.98, 67.00, 62.23, 56.65, 50.32, 41.77, 40.79, 40.40, 39.92, 37.97, 37.52, 33.17, 32.17, 31.97, 31.54, 31.43, 30.46, 30.30, 28.96, 20.70, 19.29, 17.29, 16.43, 14.68. HRMS (ESI, positive) *m*/*z* for C_27_H_42_NaO_2_S [M + Na]^+^: calc. 453.2798, found 453.2802. HPLC purity: 95.6%.

#### (2R,3R,4S,5R,6R)-2-(Acetoxymethyl)-6-(((4S,5′R,6aR,6bS,8aS,8bR,9S,10R,11aS,12aS,12bS)-5′,6a,8a,9-tetramethyl-1,3,3′,4,4’,5,5′,6,6a,6b,6’,7,8,8a,8b,9,11a,12,12a,12b-icosahydrospiro­[naphtho­[2’,1’:4,5]­indeno­[2,1-*b*]­furan-10,2’-pyran]-4-yl)­oxy)­tetrahydro-2H-pyran-3,4,5-triyl Triacetate (**15**)

Acetobromo-α-d-glucose (0.79 g, 1.93 mmol) was added to a solution of diosgenin (0.2 g, 0.48 mmol) and Ag_2_CO_3_ (1.064 g, 3.86 mmol) in anhydrous DCM (10 mL). The reaction mixture was stirred at room temperature for 48 h under argon atmosphere. The insoluble Ag_2_CO_3_ was filtered on Celite, and the filtrate was concentrated under reduced pressure. The residue was subjected to flash column chromatography (EtOAc/hexanes = 1/4) to afford the compound **15** (0.22 g, 60%). ^1^H NMR (600 MHz, CDCl_3_) δ (ppm) 5.34 (d, *J* = 5.1 Hz, 1H), 5.18 (t, *J* = 9.5 Hz, 1H), 5.05 (t, *J* = 9.7 Hz, 1H), 4.93 (dd, *J* = 9.6, 8.0 Hz, 1H), 4.58 (d, *J* = 8.0 Hz, 1H), 4.39 (m, *J* = 7.4 Hz, 1H), 4.24 (dd, *J* = 12.2, 4.8 Hz, 1H), 4.09 (dd, *J* = 12.2, 2.4 Hz, 1H), 3.66 (ddd, *J* = 10.2, 4.9, 2.5 Hz, 1H), 3.47 (td, *J* = 11.5, 4.5 Hz, 2H), 3.35 (t, *J* = 10.9 Hz, 1H), 2.33–2.11 (m, 2H), 2.05 (s, 3H), 2.03 (s, 3H), 2.00 (s, 3H), 1.98 (s, 3H), 1.92–1.00 (m, 17H), 0.99 (s, 3H), 0.95 (d, *J* = 6.9 Hz, 3H), 0.77 (d, *J* = 5.1 Hz, 6H). ^13^C NMR (150 MHz, CDCl_3_) δ (ppm) 170.67, 170.33, 169.40, 169.29, 140.37, 121.88, 109.29, 99.63, 80.79, 79.97, 72.91, 71.69, 71.50, 68.55, 66.85, 62.11, 56.49, 50.07, 41.61, 40.27, 39.74, 38.89, 37.15, 36.85, 32.08, 31.84, 31.41, 30.29, 29.70, 29.43, 28.80, 20.83, 20.75, 20.71, 20.63, 20.60, 19.37, 17.13, 16.27, 14.52.

#### (2R,3S,4S,5R,6R)-2-(Hydroxymethyl)-6-(((4S,5′R,6aR,6bS,8aS,8bR,9S,10R,11aS,12aS,12bS)-5′,6a,8a,9-tetramethyl-1,3,3′,4,4’,5,5′,6,6a,6b,6’,7,8,8a,8b,9,11a,12,12a,12b-icosahydrospiro­[naphtho­[2’,1’:4,5]­indeno­[2,1-*b*]­furan-10,2’-pyran]-4-yl)­oxy)­tetrahydro-2H-pyran-3,4,5-triol (**16**)

0.5 M solution fo sodium methoxide in MeOH (5.80 mL) was added to a solution of compound **15** (0.22 g, 0.29 mmol) in THF (7 mL). The reaction mixture was stirred at room temperature for 12 h under argon atmosphere. The mixture was quenched with 0.1 M solution of HCl (10 mL), partitioned with DCM (10 mL x 2), washed with brine (10 mL), dried over anhydrous MgSO_4_, concentrated under reduced pressure. The residue was subjected to flash column chromatography (DCM/MeOH = 9/1) to afford the compound **16** (0.11 g, 66%). ^1^H NMR (600 MHz, CDCl_3_) δ (ppm) 5.33 (d, *J* = 5.0 Hz, 1H), 4.87 (m, 3H), 4.42 (t, *J* = 5.8 Hz, 1H), 4.28 (m, 1H), 4.22 (d, *J* = 7.7 Hz, 1H), 3.69–3.60 (m, 1H), 3.51–3.36 (m, 4H), 3.20 (t, *J* = 11.0 Hz, 1H), 3.16–2.96 (m, 3H), 2.89 (td, *J* = 8.4, 4.0 Hz, 1H), 2.42–2.33 (m, 1H), 2.12 (t, *J* = 13.8 Hz, 1H), 2.03–1.02 (m, 24H), 0.97 (s, 3H), 0.91 (d, *J* = 6.8 Hz, 3H), 0.74 (d, *J* = 3.1 Hz, 6H). ^13^C NMR (150 MHz, CDCl_3_) δ (ppm) 140.98, 121.50, 108.89, 101.23, 80.67, 77.31, 77.24, 73.95, 70.60, 66.40, 63.27, 62.28, 61.59, 56.23, 50.03, 41.58, 38.78, 37.29, 36.87, 32.03, 31.95, 31.48, 31.41, 30.29, 29.73, 28.96, 20.87, 19.61, 17.56, 16.48, 15.14. HRMS (ESI, positive) *m*/*z* for C_33_H_52_NaO_8_ [M + Na]^+^: calc. 599.3554, found 599.3557. HPLC purity: 100%.

### Bioassay Reagents

Dioscin and diosgenin were purchased from Merck (St. Louis, MO, USA) and were used without further purification. The synthesized derivatives of diosgenin were prepared according to the Supporting Information. BV-2 cells, mouse microglia, were obtained from Dr. K. D. Park at KIST, and HT22 cells, mouse hippocampal neuronal cell line, were obtained from Dr. H. Ryu at KIST. THP-1 dual cells were purchased from InvivoGen (Hong Kong). Dulbecco’ Modified Eagle’s Medium (DMEM) and fetal bovine serum (FBS) were obtained from Hyclone (Logan, UT, USA). Penicillin-streptomycin solution was purchased from Corning (Corning, NY, USA) and MycoGuard was obtained from Biomax (Gyeonggi-do, Republic of Korea). LY96 protein was purchased from Sigma-Aldrich (St. Louis, MO, USA). CellTiter-Glo (CTG) reagent was obtained from Promega (Madison, WI, USA). NucleoSpin RNA Plus was purchased from Macherey-Nagel (Düren, Germany). IQ SYBR Green Supermix was obtained from Bio-Rad (Hercules, CA, USA), and individual primers were purchased from Bioneer (Daejeon, Republic of Korea). RIPA lysis buffer from Biosesang (Yongin-si, Republic of Korea) contained protease inhibitors and phosphatase inhibitors (Thermo Fisher). A 5% Bovine Serum Albumin (BSA, Biosesang) was prepared in Tris-buffered saline with Tween 20 (TBST, Biosesang) solution. ECL prime solution was obtained from Amersham (England). Cell Tracker Green CMFDA Solution (Thermo fisher) was diluted with prewarmed Opti-MEM medium (Gibco). All other chemicals were purchased from Merck (St. Louis, MO, USA) and Thermo Fisher Scientific (Waltham, MA, USA).

#### Cell Culture

BV-2 cells (mouse microglia) and HT22 cells (mouse hippocampal neuronal cell line) were cultured in DMEM supplemented with 10% (v/v) heat-inactivated FBS (Hyclone), 1% (v/v) antibiotic-penicillin solution (Corning), and 0.1% (v/v) Myco-Guard mycoplasma elimination reagent (Biomax). Cells were incubated at 37 °C in a 5% CO_2_ incubator under a humidified atmosphere. They were cultured in a 100 mm cell culture dish.

#### Griess Assay

Nitric oxide (NO) production was assessed by measuring the nitrite concentration in the supernatant of cultured BV-2 cells. Cells were seeded at a density of 3 × 10^5^ cells/mL in 384-well plates and incubated overnight. The cells were then stimulated with 100 ng/mL of LPS in the presence or absence of test compounds for 24 h, followed by brief centrifugation. The supernatant was mixed with an equal volume of Griess reagent (1% sulfanilamide, 0.1% naphthyl ethylenediamine dihydrochloride, and 2.5% H_3_PO_4_) and incubated at room temperature for 15 min. Nitrite concentrations were determined by measuring the absorbance of the supernatant at 560 nm. A sodium nitrite standard curve was used for quantification. Cell viability was determined by measuring luminescence. After adding 20 μL of CTG reagent, the cells were incubated at room temperature for 10 min, protected from direct light. The luminescence signals were measured using a Tecan microplate reader (BioTek). Each value was normalized to cells treated with DMSO.

#### RT-PCR Analysis

Cellular mRNA was prepared using NucleoSpin RNA Plus (Macherey-Nagel) according to the manufacturer’s instructions. A total of 1000 ng of RNA from each sample was subjected to reverse transcription at 25 °C for 5 min, 42 °C for 30 min, and then 85 °C for 5 min. The resulting cDNA was mixed with IQ SYBR Green Supermix (Bio-Rad) and individual primers (Bioneer) for RT-PCR analysis using a real-time thermal cycler (Bio-Rad, CFX Connect). GAPDH was used as a housekeeping gene to normalize mRNA levels. The primer sequences are provided in the Supporting Information.

#### Enzyme-Linked Immunosorbent Assay (ELISA)

Cell culture supernatants were collected and analyzed for cytokine levels. The concentration of tumor necrosis factor-α (TNFα) in the supernatants was quantified using ELISA kits (R&D systems) according to the manufacturer’s instructions. For cell viability assessment, the remaining cells were supplemented with Opti-MEM medium (Thermo Fisher) and 10% (v/v) CellTiter 96 AQueous One Solution (MTS, Promega). The cells were then incubated for 30 min at 37 °C in a 5% CO_2_ in a humidified atmosphere. Absorbance was measured at 490 nm wavelength.

#### Western Blot

Cells were harvested in RIPA lysis buffer (Biosesang) containing protease inhibitors and phosphatase inhibitors (Thermo Fisher). After cell lysis, each sample was centrifuged at 14,000 rpm for 20 min. The supernatant was collected, and protein concentration was normalized using the BCA assay (Thermo Fisher). The prepared samples were analyzed by SDS-PAGE and subsequently subjected to Western blotting. The proteins were transferred to a PVDF membrane and blocked with 5% bovine serum albumin (BSA; Biosesang) in a solution of Tris-buffered saline with Tween 20 (TBST; Biosesang) for 1 h. The membrane was incubated with the primary antibody in TBST at 4 °C overnight, followed by washing with TBST. The primary antibodies used included: anti-Vinculin (1:3000, 13901S; CST), anti-GAPDH (1:3000, 5174S; CST) anti-pp65 (1:1000, 3033S; CST), anti-p65 (1:1000, 8242S; CST), anti-iNOS (1:1000, 13120S; CST). Then, the membrane was incubated with a secondary antibody (1:3000, 7074S, CST) at room temperature for 1 h, followed by washing with TBST. Finally, the membrane was developed using an ECL prime solution (Amersham), and chemiluminescence signals were analyzed with a ChemiDoc imaging system (Bio-Rad).

#### Centrifugal Ultrafiltration Assay

The recombinant protein fragment of human LY96 was concentrated to 10 μM in PBS buffer (pH 7.4). Compound **8** was diluted to the same concentration in PBS buffer. The compound solution (20 μL) was loaded onto centrifugal devices (NANOSEP 3K OMEGA) with either 20 μL of Tris buffer or 20 μL of LY96 protein, then incubated at room temperature for 10 min. The flow-through from a 1 min spin was measured using a Nanodrop spectrophotometer (Eppendorf BioSpectrometer basic). Unbound compound **8** was monitored by its UV absorbance at 230 nm.

#### Immunofluorescence

Cells were seeded in a confocal dish and maintained for 1 d. The cells were then stimulated with 200 ng/mL of LPS (Sigma–Aldrich) in the absence or presence of test compounds for 24 h. After aspirating the media and washing with PBS, 200 μL of a 100% ice-cold MeOH was added, and the cells were incubated at −20 °C for 5 min. The solution was then aspirated, and the sample was washed three times with PBS. For permeabilization to enable antibody binding, 300 μL of 0.1% Triton-X-100 in PBS solution was added and incubated at room temperature for 15 min. The solution was then removed, and the sample was washed three times with PBS. Samples were blocked with 2% BSA in PBS solution at room temperature for 1 h. After removing the blocking solution, the primary antibody solution of 1% BSA in PBS was added and incubated overnight at 4 °C. The primary antibody used was goat anti-Iba1 (1:300; abcam, ab5076). After incubation, the primary antibody solution was removed, and the sample was washed three times with PBS. The secondary antibody solution of 1% BSA in PBS was then added and incubated at room temperature for 1 h. After incubation, the antibody solution was aspirated, and the sample was washed three times with PBS. For nuclear staining, Hoechst 33342 was diluted in PBS, added to each well, and incubated at room temperature for 5 min. The Hoechst 33342 was then removed, and the chamber was filled with PBS for subsequent imaging using a microscope.

#### Coculture Analysis with Flow Cytometry Analysis

BV-2 and HT22 cells were seeded at a density of 5 × 10^5^ cells/mL in 12-well plates and 3 × 10^5^ cells/mL in 6-well plates, respectively, and incubated overnight. The BV-2 cells were then stimulated with 200 ng/mL of LPS in the presence or absence of test compounds for 4 h. Directly before coculture, HT22 cells were incubated with 5 μM of CellTracker Green CMFDA Solution (Thermo Fisher) diluted in prewarmed Opti-MEM medium (Gibco) for 45 min at room temperature. After incubation, HT22 cells were harvested and seeded directly onto BV-2 cells at a density of 4 × 10^6^ cells/mL for 20 h. The cells were then harvested and suspended in 1 mL of PBS. The suspension was centrifuged at 200 g for 5 min at room temperature, and the PBS was aspirated. The cell pellet was resuspended in 600 μL of PBS. Flow cytometry analysis was performed using a CytoFLEX flow cytometer (BECKMAN COULTER) with a 488 nm laser line for excitation. Green fluorescence was measured, and at least 20,000 events were collected.

#### Computational Study

To identify potential targets of compound **8**, we conducted reverse docking, a computational method that predicts binding energy by docking a compound to multiple protein structures.^33^ We performed reverse docking using AD3 program, which incorporates protein structures from three databases: RCSB PDB, AlphFold DB, and Uniprot. The program includes a feature to identify binding sites. Known binding sites were determined by PLIP and further evaluated with three binding site prediction tools: P2Rank, Fpocket and DeeSurf. Protein candidates were ranked based on their Z-Score Energy and AK-Score2 Energy values, representing the interaction strength between the ligand and protein targets. From these, we selected a candidate protein associated with the inflammation signaling pathway. For the molecular docking study, we employed Discovery Studio 2024 (BIOVIA (Dassault Systèmes)). The complex of human Toll-like receptor 4 (TLR4) and MD-2 (PDB: 3FXI) was retrieved from the RCSB PDB database (http://rcsb.org/pdb).^34^ Only the TLR4 structure was extracted from the complex for docking. Prior to docking, all water molecules were removed from the protein to ensure accurate ligand-protein interaction analysis. The binding site for the ligand was defined based on the lipopolysaccharide (LPS) binding site of 3FXI. Protein preparation followed standard protocols in Discovery Studio, including the Clean Protein protocol and Prepare Protein protocol under the CHARMm force field.^32^ Compound **8** was similarly prepared under the same force field, undergoing standard preparation protocol and energy minimization at pH 7.4. The CDOCKER algorithm^35^ in Discovery Studio was employed for docking, and the interacting residues were subsequently analyzed.

#### Animals

All animal experimental procedures were conducted in accordance with the guidelines approved by the Institutional Animal Care and Use Committee (IACUC) of the Korea Institute of Science and Technology (KIST, KIST-5088–2022–03–045). Three to 4 week-old ICR mice were obtained from DBL and had free access to food and water. The mice underwent an adaptation period of 1 week and were handled daily for at least 3 days before the initiation of the behavior test. The day before each test, 5-week-old mice were injected intraperitoneally (i.p) with either saline or compound **8** (50 mg/kg). After 1 h, the mice received an intravenous (i.v) injection of either saline or LPS (1 mg/kg). Following the injections, the mice were placed in individual cages measuring 10 cm in width, 27 cm in height, and 13 cm in depth.

#### Immunohistochemistry (IHC) and Confocal Imaging

For all histological analyses, mice were deeply anesthetized and transcardially perfused with 20 mL of saline, followed by 20 mL of 4% paraformaldehyde (PFA) in 0.2 M phosphate buffer. The brains were removed, post fixed in 4% PFA, and cryoprotected in 30% sucrose at 4 °C for a day. The brains were then sectioned into 30 μm thick coronal slices using a cryostat microtome (Thermo scientific). For immunohistochemistry, sections were first incubated for 1.5 h in a blocking solution (0.3% Triton-X, 2% goat serum, and 2% donkey serum in 0.1 M PBS) and then immunostained overnight at 4 °C with a mixture of primary antibodies in a blocking solution. Primary antibodies used are as follow: chicken anti-GFAP (1:500; ab5541, Millipore), goat anti-Iba1 (1:300; abcam, ab5076), and guinea pig anti-NeuN (1:1000; Synaptic system, 266 044). After extensive washing, sections were incubated with the corresponding fluorescent secondary antibodies (1:500 for all secondary antibodies; Jackson, 703–545–155; Life technologies, A21432; Jackson, 706–605–148) for 1 h at room temperature, followed by three times washing with PBS. Fluorescent images were obtained using an A1 Nikon confocal microscope, and Z-stack images in 3 μm steps were processed for further analysis using Imaris 9 software (Bitplane). For measurement of GFAP or Iba1 intensities, surfaces were created for each GFAP+ cell using GFAP+ images or each Iba1+ cell using Iba1+ images in Imaris 9 software. The mean intensity values of GFAP or Iba1 were then collected for each region of interest (ROI). The representative 3D images were rendered using Surface function in Imaris 9 software.

#### Tail Suspension Test (TST)

The dimensions of the box were 44 cm in height, 14 cm in width, and 14 cm in depth. To prevent animals from observing or interacting with each other, each mouse was suspended within its own three-walled rectangular compartment. A suspension ring, used to hold the tail of each mouse, was positioned at the top of the box. Seventeen-centimeter tape fragments were cut, with a mark 2 cm from one end. This 2 cm portion was used to attach the tape to the tail, while the remaining 15 cm was used for suspending the mouse. The tape adhered to both the mouse’s tail and the suspension ring. For animal behavior observations, all procedures were conducted before 3 p.m. Video recordings lasted a total of 8 min, but only the 2- to 8 min period was used for immobility analysis. In the TST, immobility was defined as the mouse remaining suspended without struggling. Immobility time was measured using the Noldus analysis program.

#### Forced Swimming Test (FST)

The dimensions of the tank were 20 cm in height and 10 cm in diameter, and it was filled with water to a depth of 15 cm. The water in the tank was changed after testing each mouse. The water temperature was maintained between 21 and 23 °C. For animal behavior observations, all procedures were conducted before 3 p.m. Video recordings lasted a total of 6 min, but only the 2- to 6 min period was used for immobility analysis. In the FST, immobility was defined as the mouse remaining afloat in the water without struggling, making only movements necessary to keep its head above water. Immobility time was measured using the Noldus analysis program.

#### Sucrose Preference Test (SPT)

The day before the test, a 15 mL tube with a ball nozzle was prepared for each mouse and filled with 14 mL of water and 1% sucrose. The baseline water and sucrose intake of each mouse was measured. After completing the TST and FST, mice were provided with 14 mL of water and 1% sucrose in each tube for 24 h. The absolute intake of sucrose and water was measured separately. Sucrose preference was calculated using the following formula: preference = (sucrose intake/total intake) × 100%. Anhedonia was defined as a reduction in the sucrose preference ratio relative to the control group in the preference test.

## Supplementary Material







## Notes

Molecular formula strings (CSV)
^1^H and ^13^C NMR spectra and Supplementary figures (PDF)
CDOCKER_3FXI_ligand complex (PDB)

## Abbreviations

AD: Alzheimer’s disease
BSA: bovine serum albumin
CNS: central nervous system
CTG: CellTiter-Glo
DG: diosgenin
ELISA: enzyme-linked immunosorbent assay
FST: forced swimming test
GFAP: glial fibrillary acidic protein
HPA: hypothalamus-pituitary-adrenal
IHC: immunohistochemistry
IL-1β: interleukin-1 beta
IL-6: interleukin 6
Iba1: ionized calcium-binding adaptor molecule 1
JNK: c-Jun N-terminal kinase
LPS: lipopolysaccharide
MAPK: mitogen-activated protein kinase
NF-κB: nuclear factor kappa B
NO: nitric oxide
NOS2: nitric oxide synthase 2
PD: Parkinson’s disease
PFA: paraformaldehyde
SEAP: secreted embryonic alkaline phosphatase
SPT: sucrose preference test
SSRIs: selective serotonin reuptake inhibitors
TCAs: tricyclic antidepressants
TI: therapeutic index
TLR: toll-like receptor
TNF-α: tumor necrosis factor alpha
TST: tail suspension test
i.p: intraperitoneal
i.v: intravenous
