# Supplementary material for: Discovery of a Neuroprotective Diosgenin Derivative as a Novel Antidepressant Candidate Targeting LPS-TLR4 Signaling
Source: J Med Chem. 2026 Feb 2;69(3):3062–76. doi: 10.1021/acs.jmedchem.5c02981 (PMC12910651; doi:10.1021/acs.jmedchem.5c02981)
Supplement: Supplementary file 2 [file jm5c02981_si_002.pdf]

SUPPORTING INFORMATION

Discovery of a Neuroprotective Diosgenin Derivative as a Novel Antidepressant Candidate Targeting LPS-TLR4 Signaling

Younghun Yoo,[a,b,†] Soo Yeon Baek,[a,†] Hyelim Lee,[a] Jeehee Lee,[a,g] Hyowon Lee,[d] Haeun Lee,[a,b] Hyeonji Ma,[a,b] Yujin Kim,[a,h] Hoon-Seong Choi,[e] Jeong Tae Lee,[f] Jae Yeol Lee,[h] Min-Ho Nam,[b,d] Sanghee Lee,[a,c,*] and Byungsun Jeon[a,b,*]

[a]Medicinal Materials Research Center, Biomedical Research Division, Korea Institute of Science and Technology, Seoul 02792, Republic of Korea

[b]Division of Bio-Medical Science and Technology, KIST School, University of Science and Technology, Seoul 02792, Republic of Korea

[c]KHU-KIST Department of Converging Science and Technology, Kyunghee University, Seoul 02447, Republic of Korea

[d]Center for Brain Disorders, Brain Science Institute, Korea Institute of Science and Technology, Seoul 02792, Republic of Korea

[e]Research Animal Resources Center, Research Resources Division, Korea Institute of Science and Technology, Seoul 02792, Republic of Korea

[f]Department of Chemistry and Institute of Applied Chemistry, Hallym University, Chuncheon 24252, Republic of Korea

[g]Department of HY-KIST Bio-convergence, Hanyang University, Seoul 04763, Republic of Korea

[h]Research Institute for Basic Sciences and Department of Chemistry, College of Sciences, Kyung Hee University, Seoul 02447, Republic of Korea

[†] These authors contributed equally.

Corresponding Authors

bsjeon@kist.re.kr; slee@kist.re.kr

I. NMR SPECTRA ………………….....................................……………………………………..S3

II. MASS SPECTRA..………………………………....S17

III. HPLC SPECTRA……………………………………………………………………………......S19

IV. *IN VITRO* ACTIVITY ASSAYS OF DIOSGENIN DERIVATIVES..S22

V. CELL VIABILITY TEST USING COMPOUND **8**..S24

VI. PCR PRIMER SEQUENCES..S25

VII. IN VIVO PHARMACOKINETIC STUDIES...S26

VIII. PLASMA STABILITY ANALYSIS..S27

IX. COMPUTATIONAL STUDIES...S27

X. SOLUBILITY ANALYSIS..S29

XI. REFERENCES..S31

I. NMR Spectra

Figure S1. ^{1}H- and ^{13}C-NMR of compound **4**.

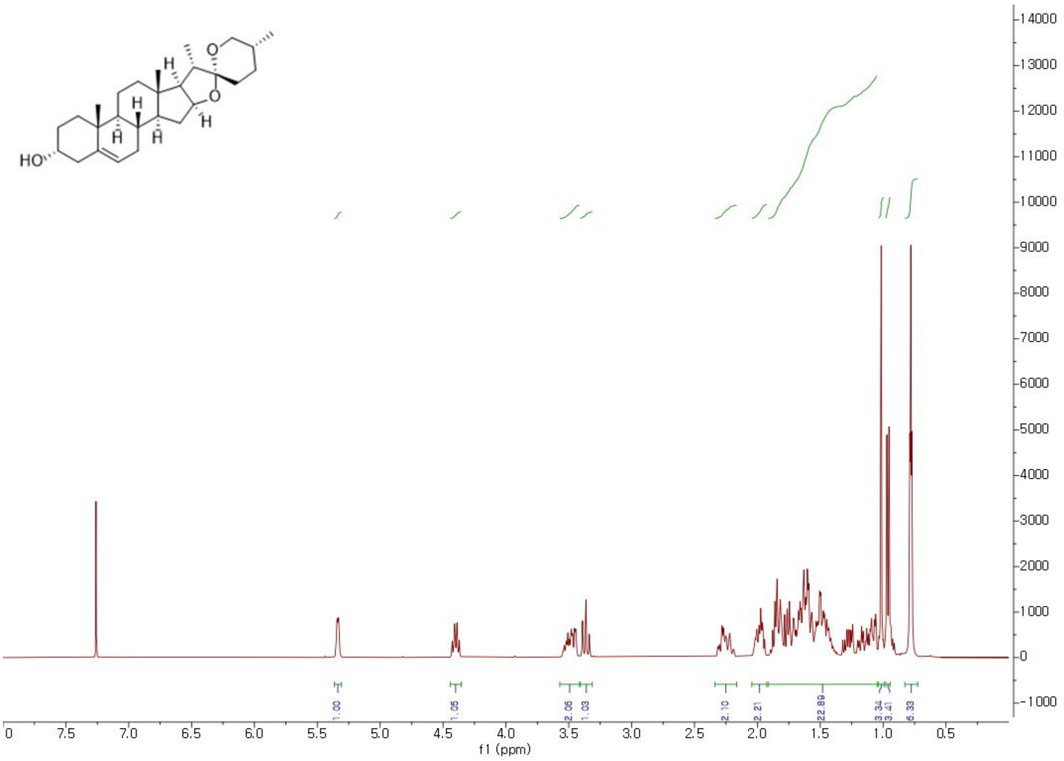

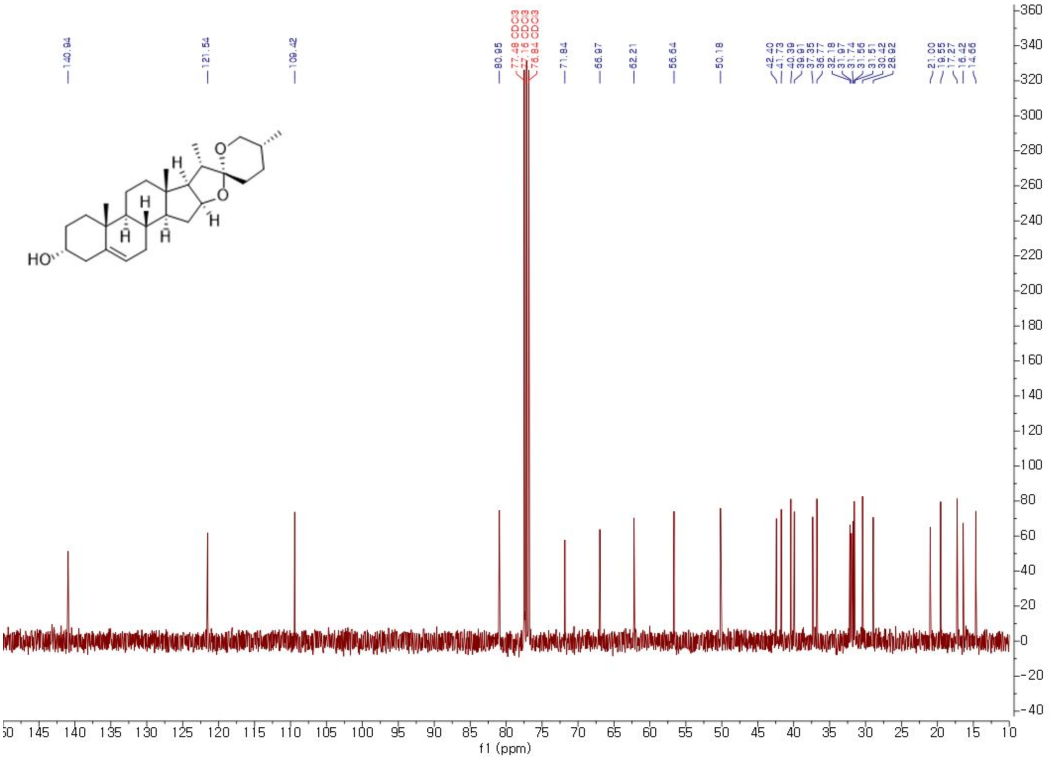

Figure S2. ^{1}H- and ^{13}C-NMR of compound **5**.

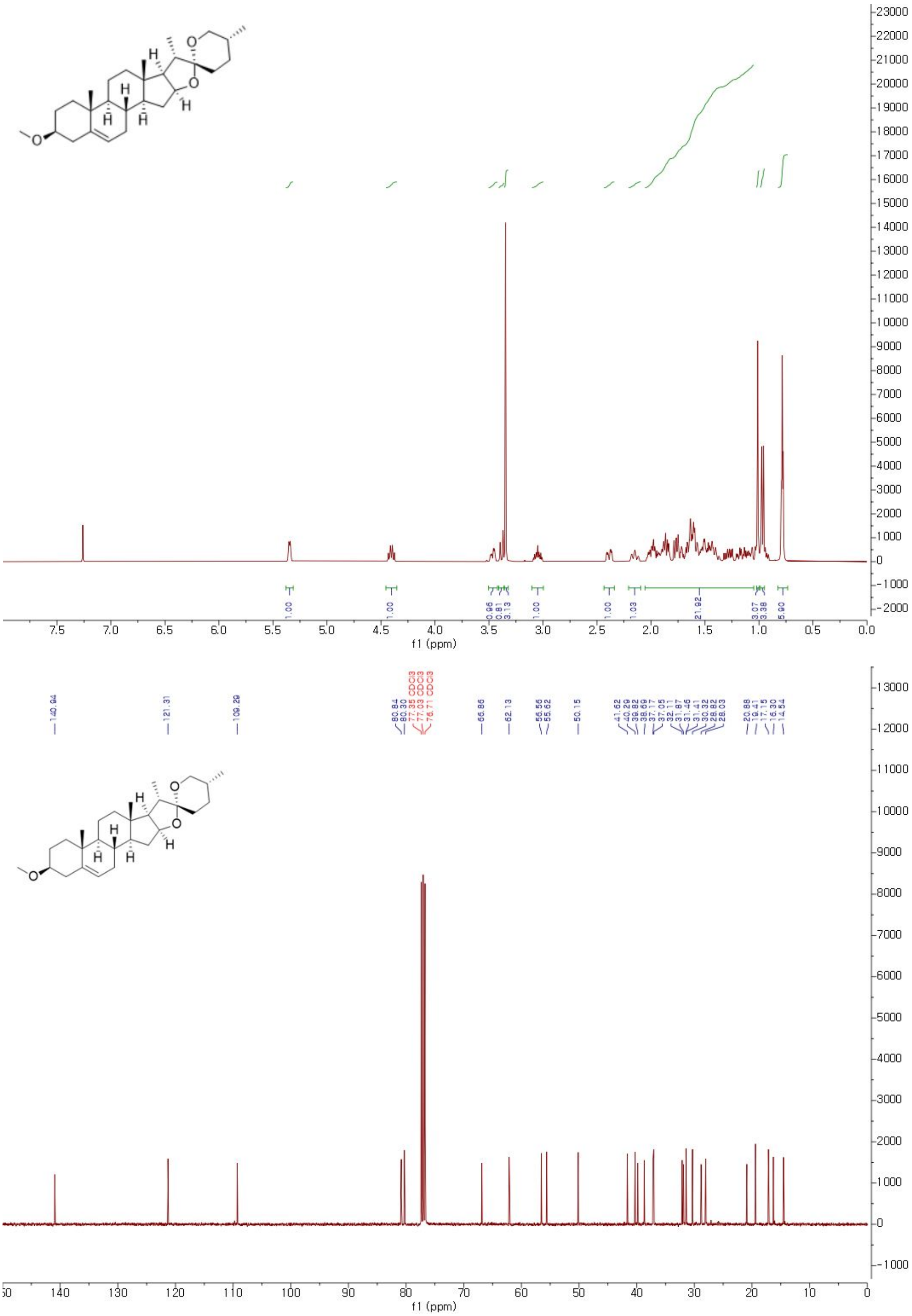

Figure S3. ^{1}H- and ^{13}C-NMR of compound **6**.

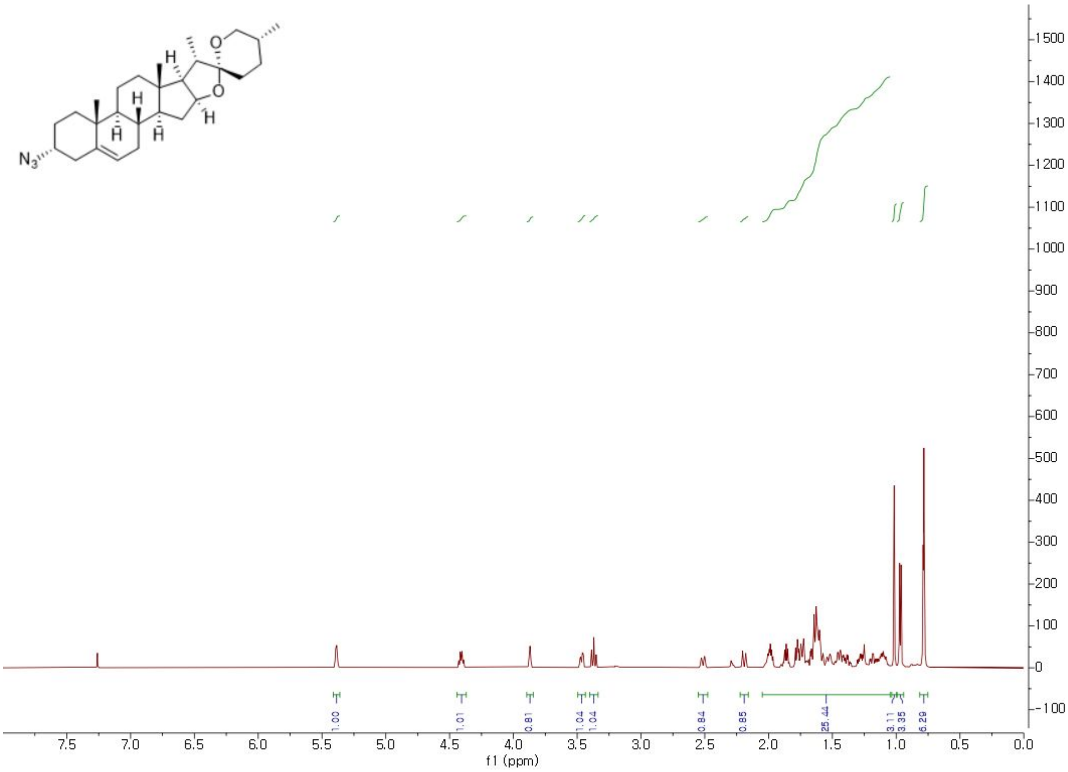

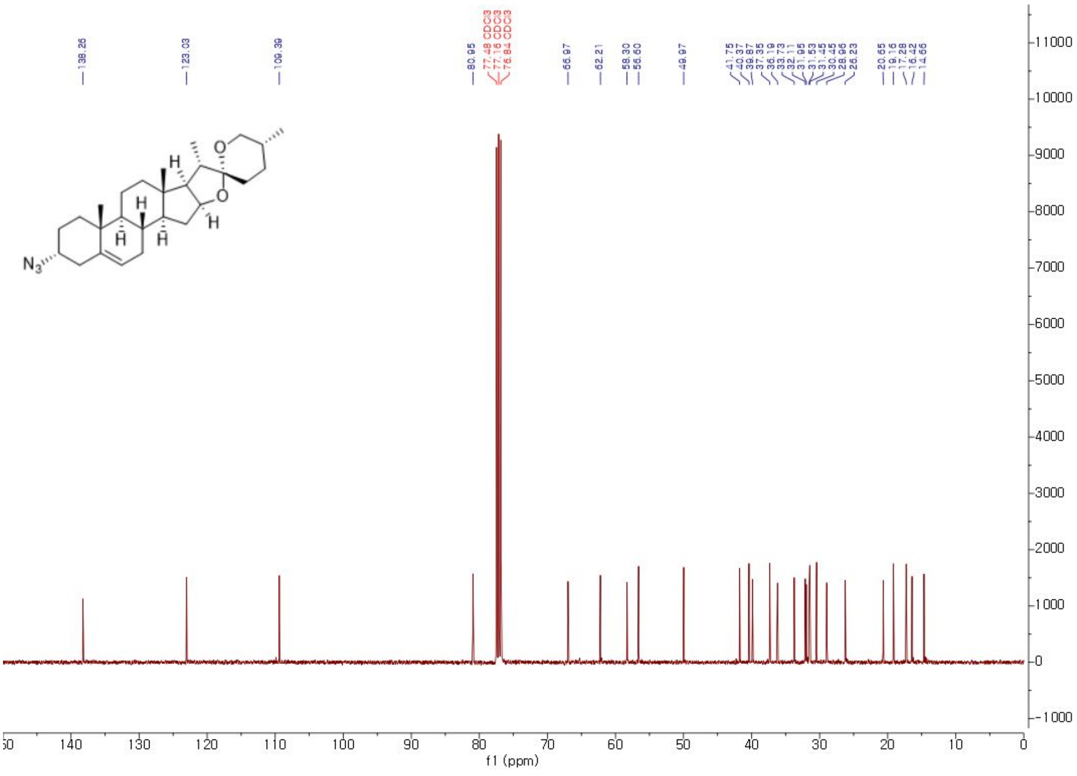

Figure S4. ^{1}H- and ^{13}C-NMR of compound **7**.

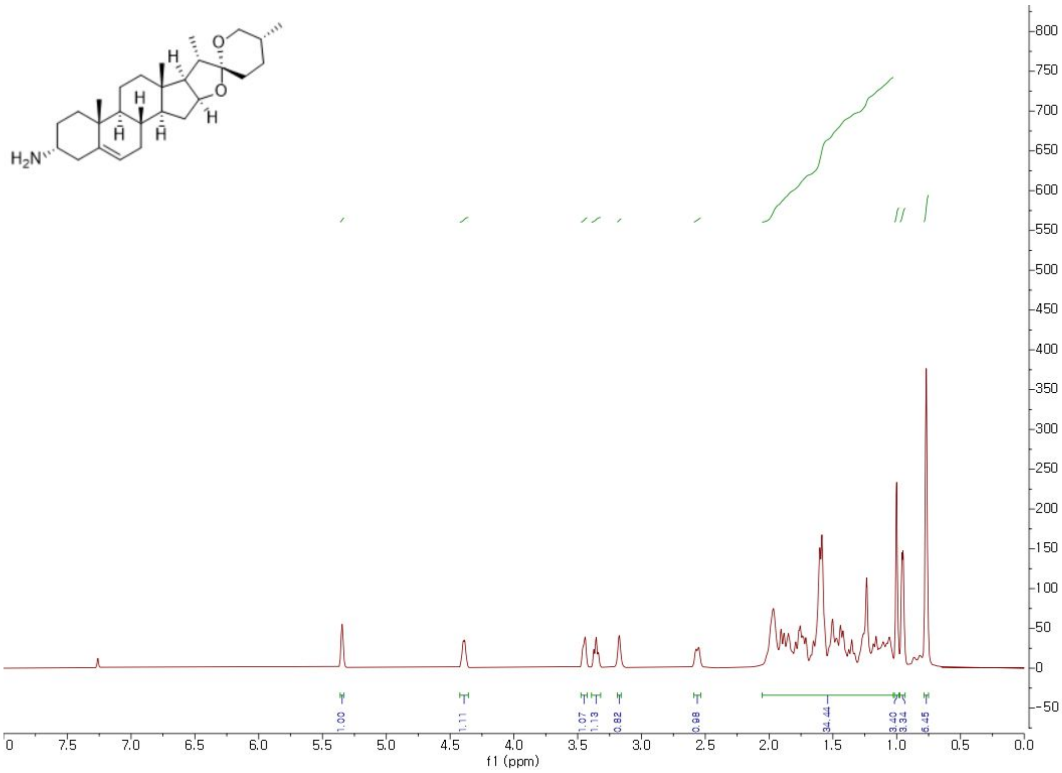

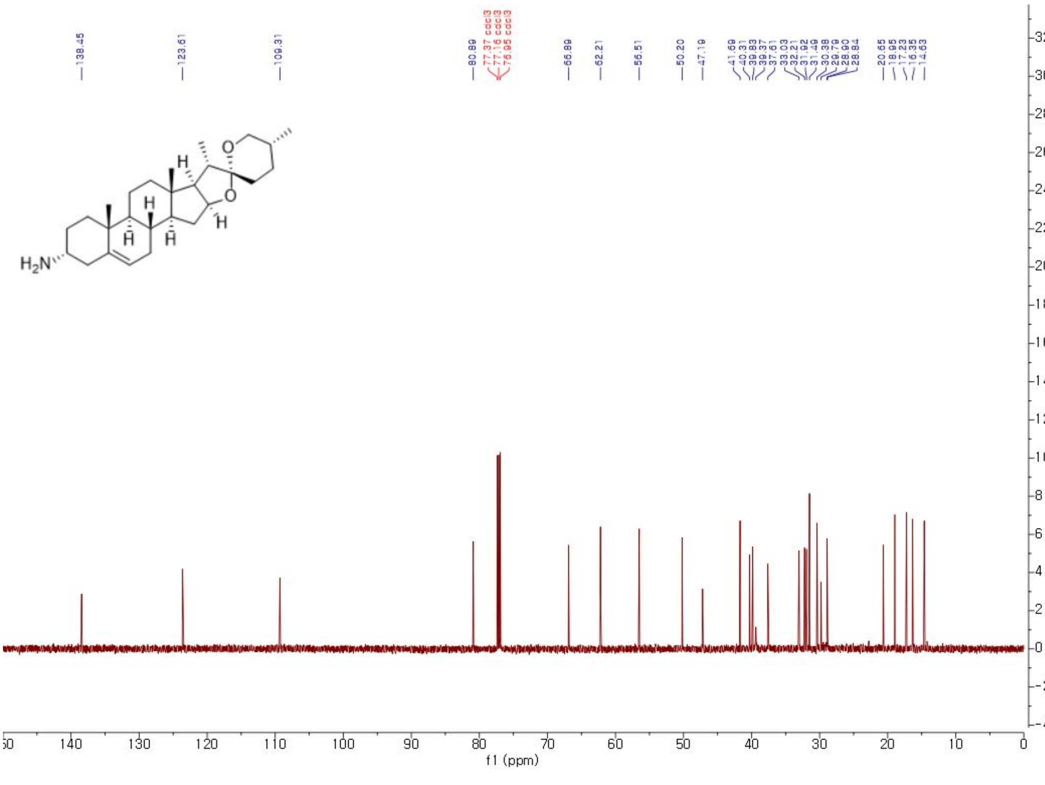

Figure S5. ^{1}H-, ^{13}C-NMR, and HSQC of compound **8**.

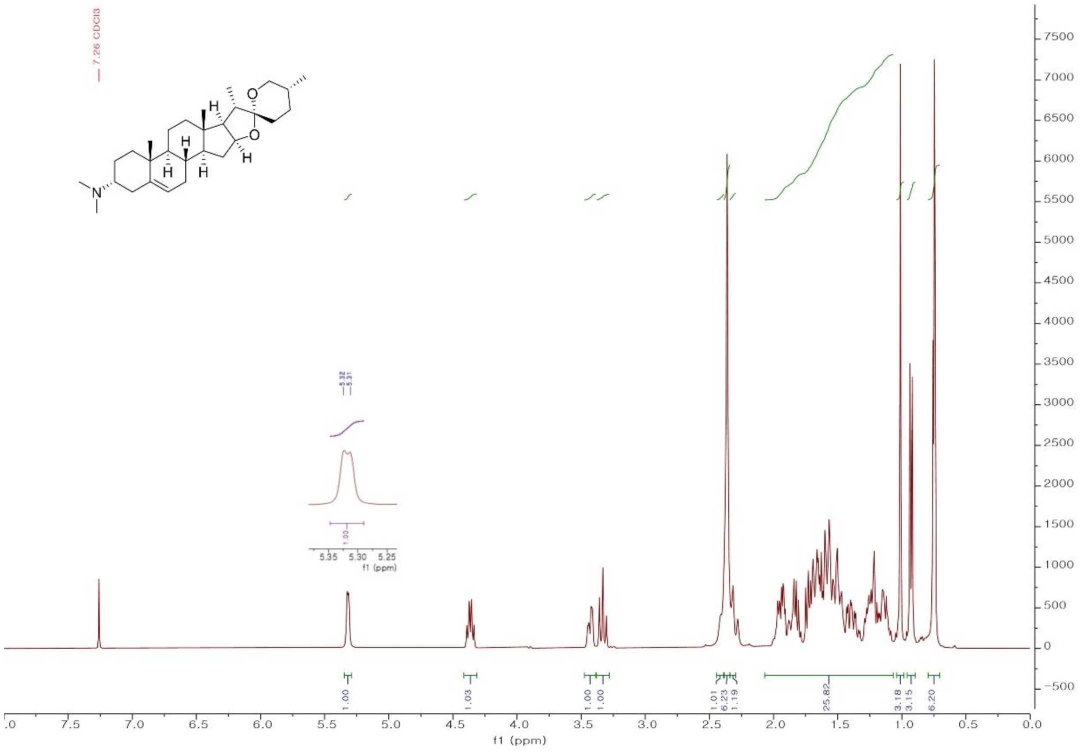

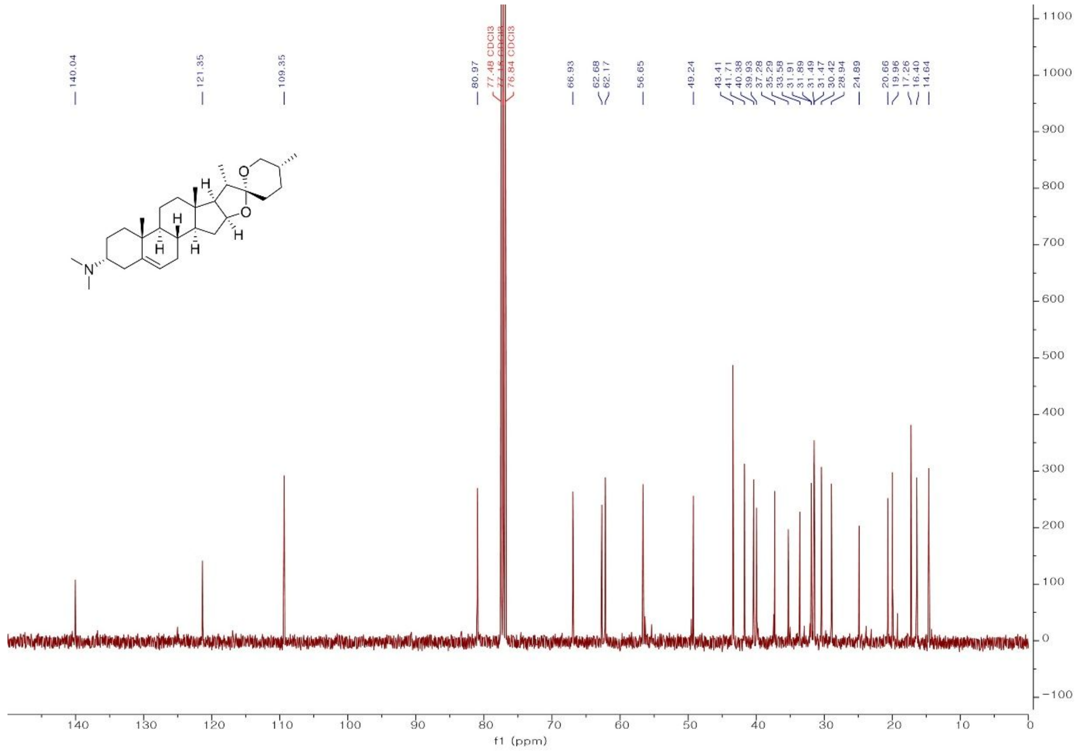

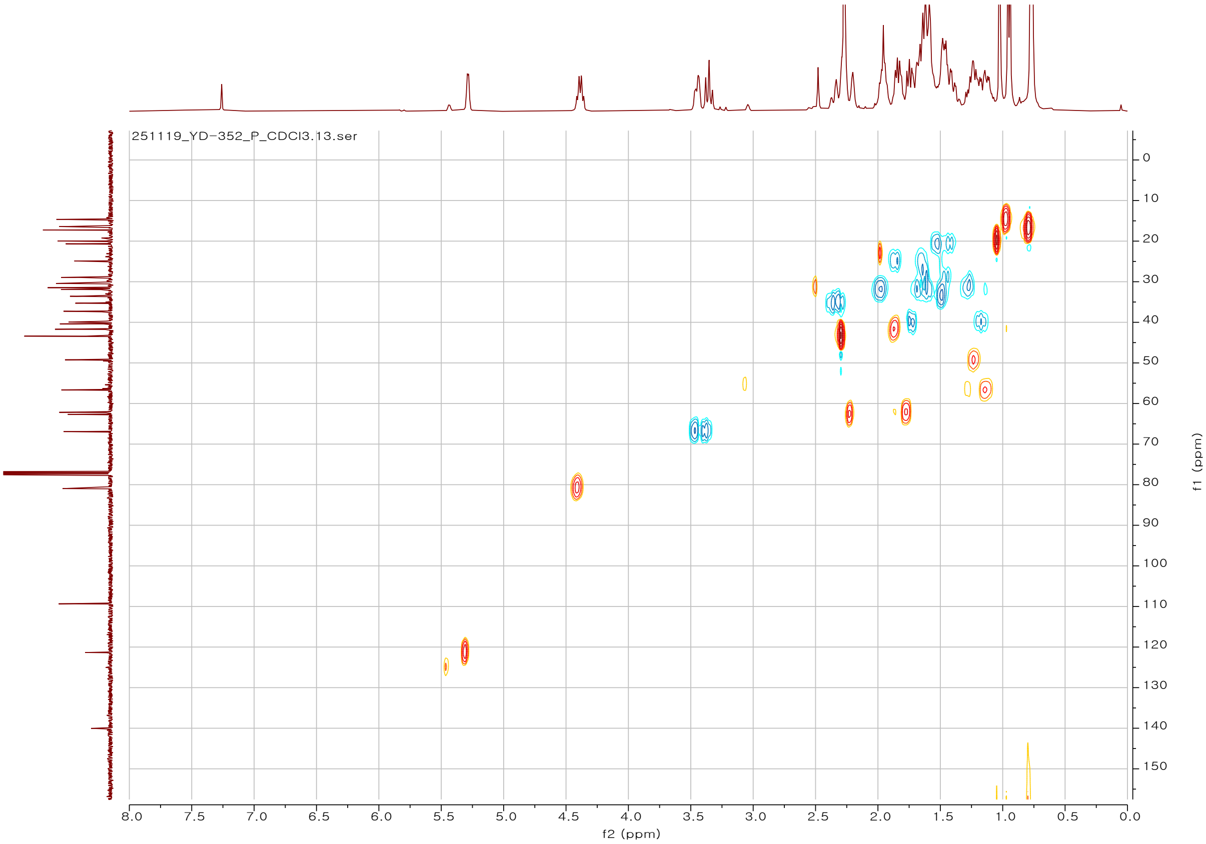

Figure S6. ^{1}H- and ^{13}C-NMR of compound **9**.

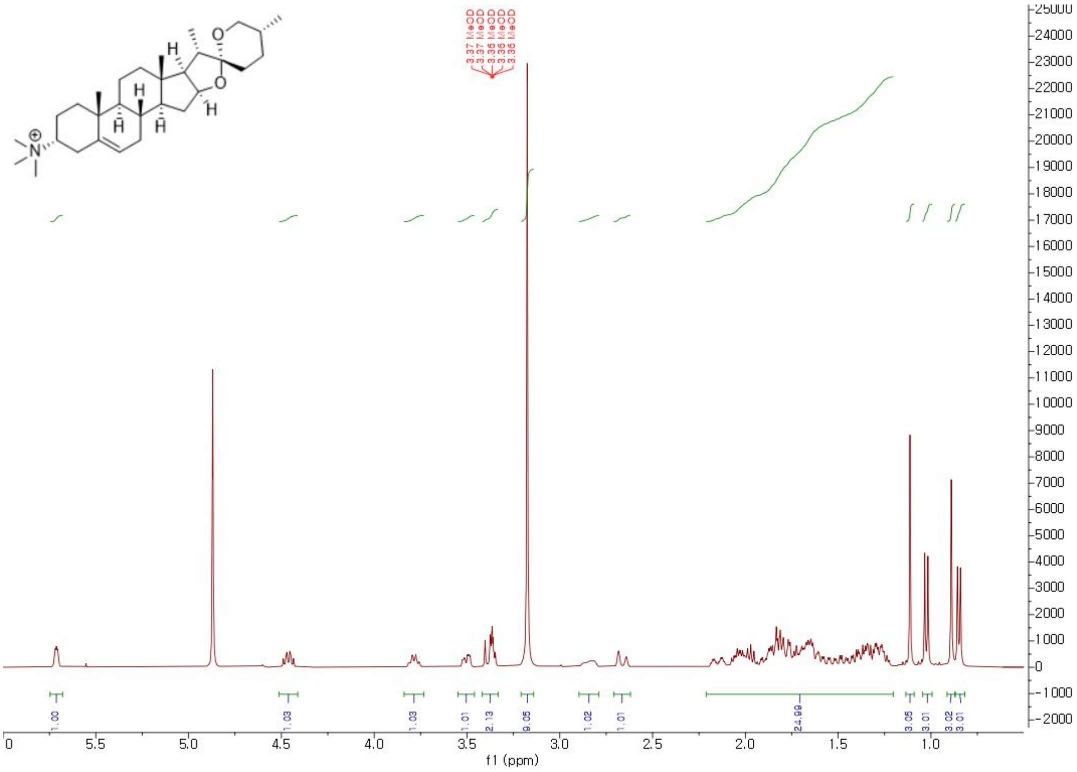

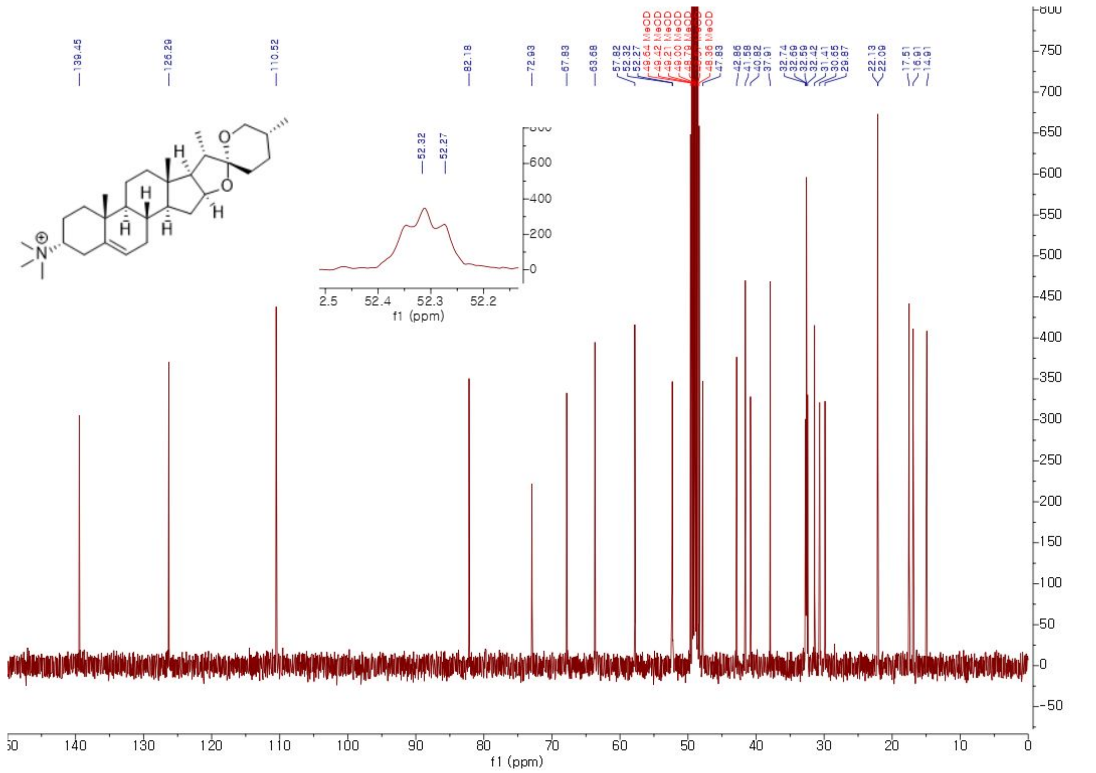

Figure S7. ^{1}H- and ^{13}C-NMR of compound **10**.

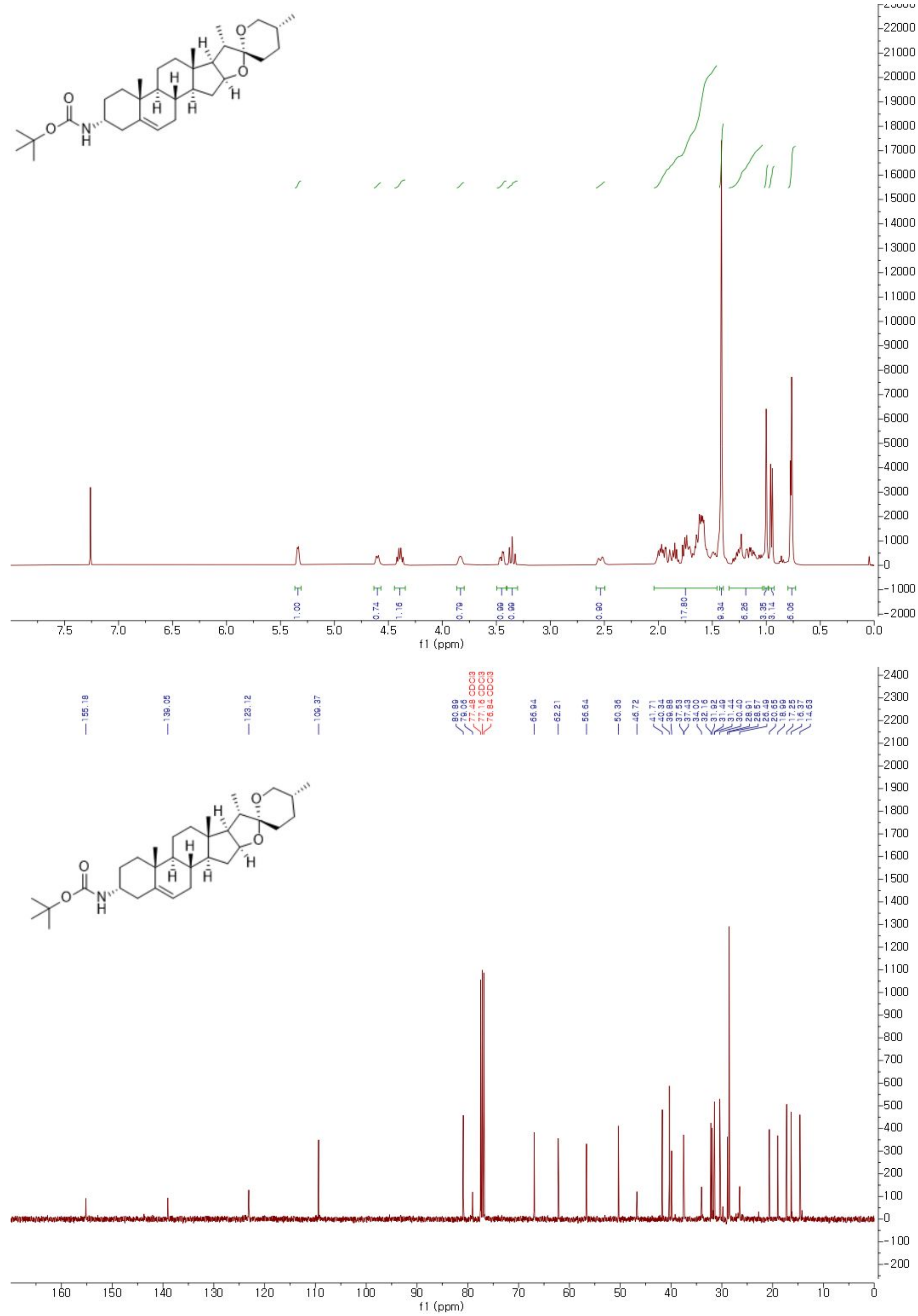

Figure S8. ^{1}H- and ^{13}C-NMR of compound **11**.

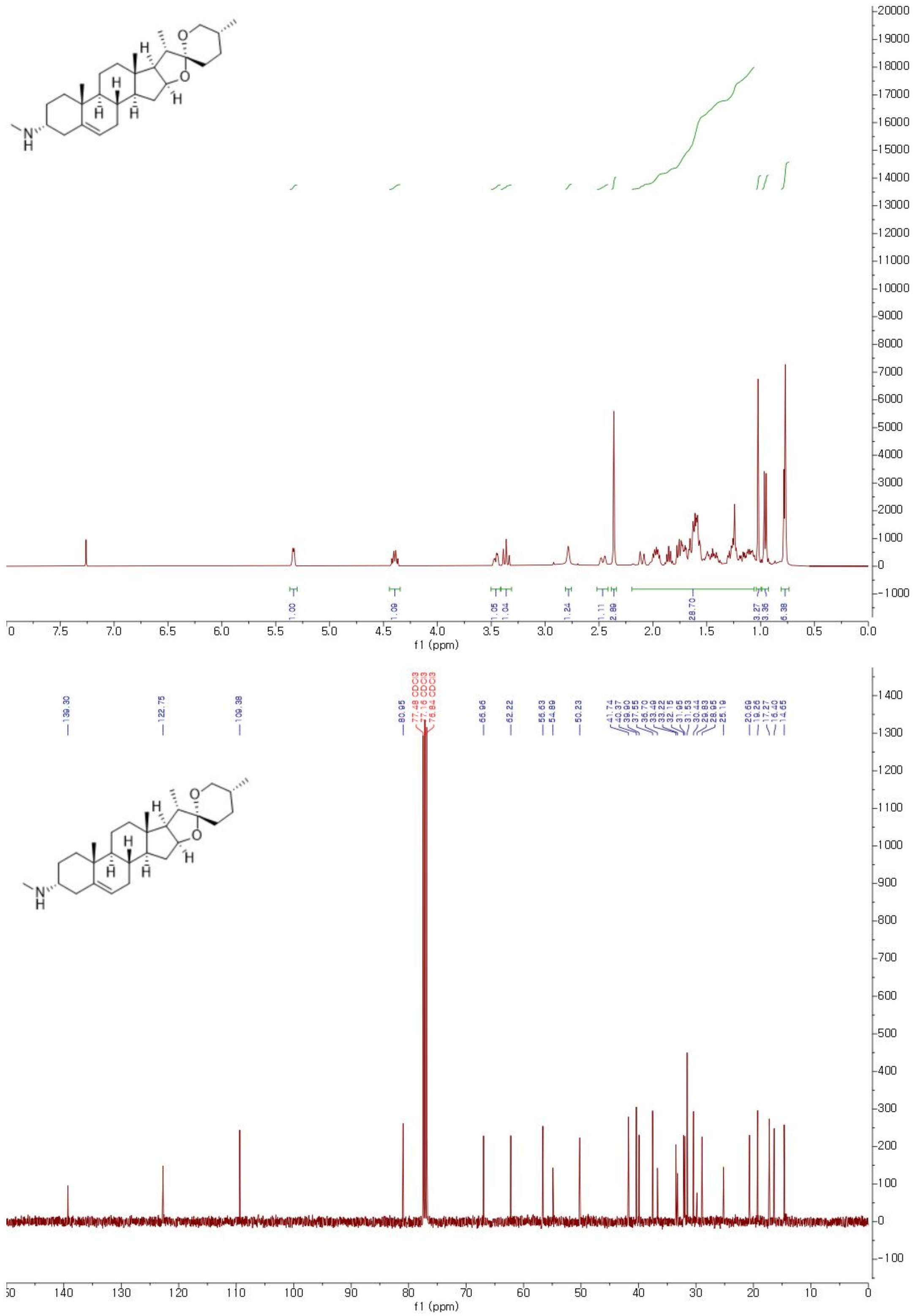

Figure S9. ^{1}H- and ^{13}C-NMR of compound **12**.

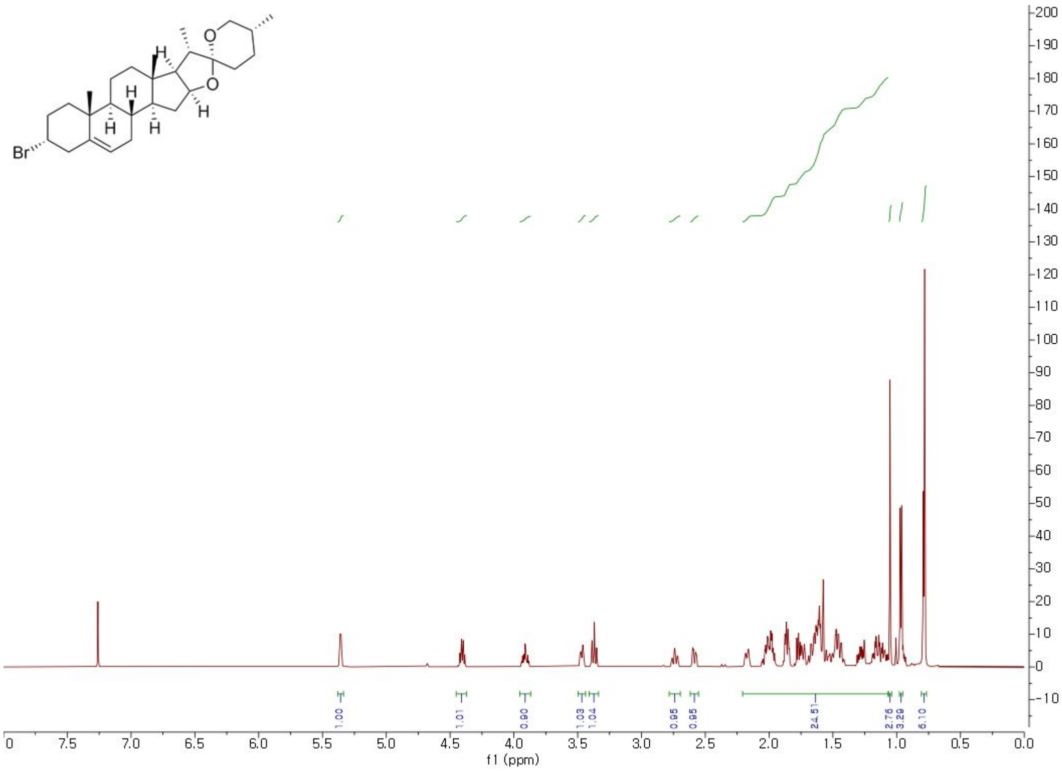

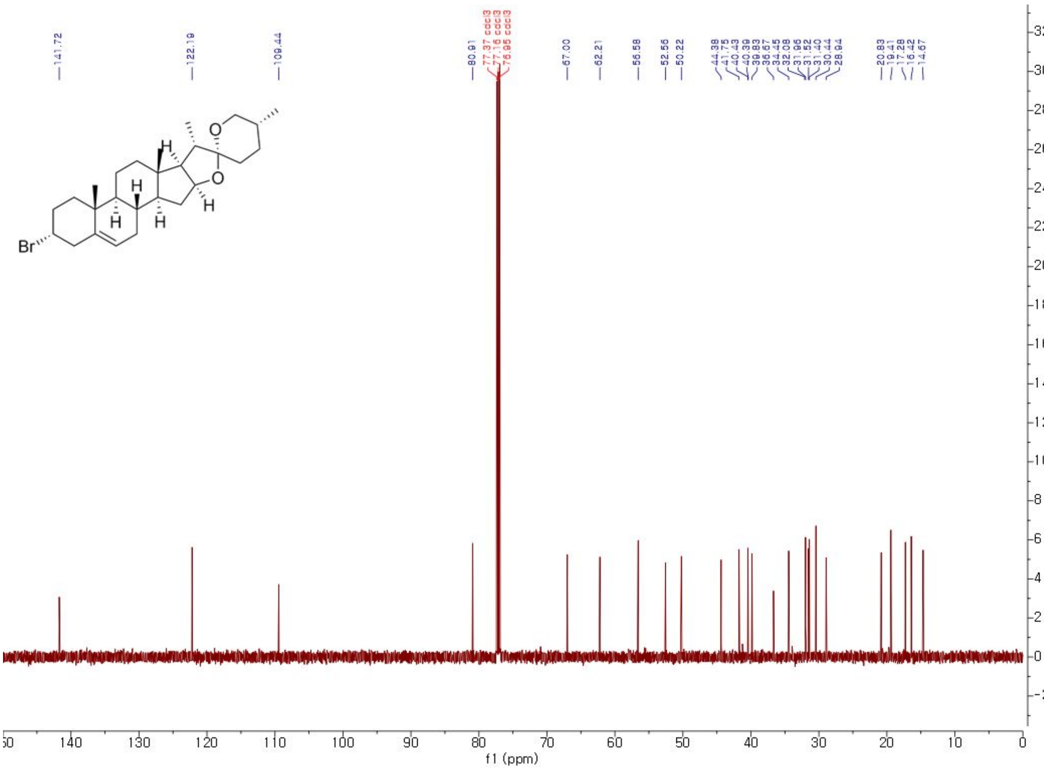

Figure S10. ^{1}H- and ^{13}C-NMR of compound **13**.

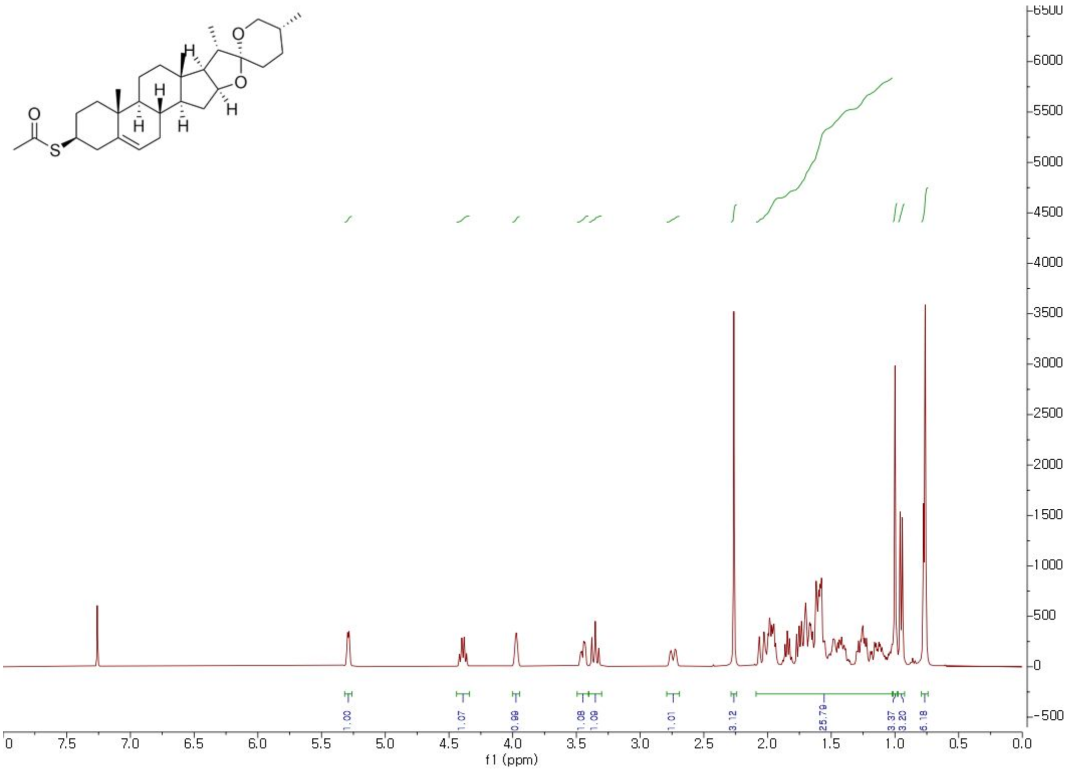

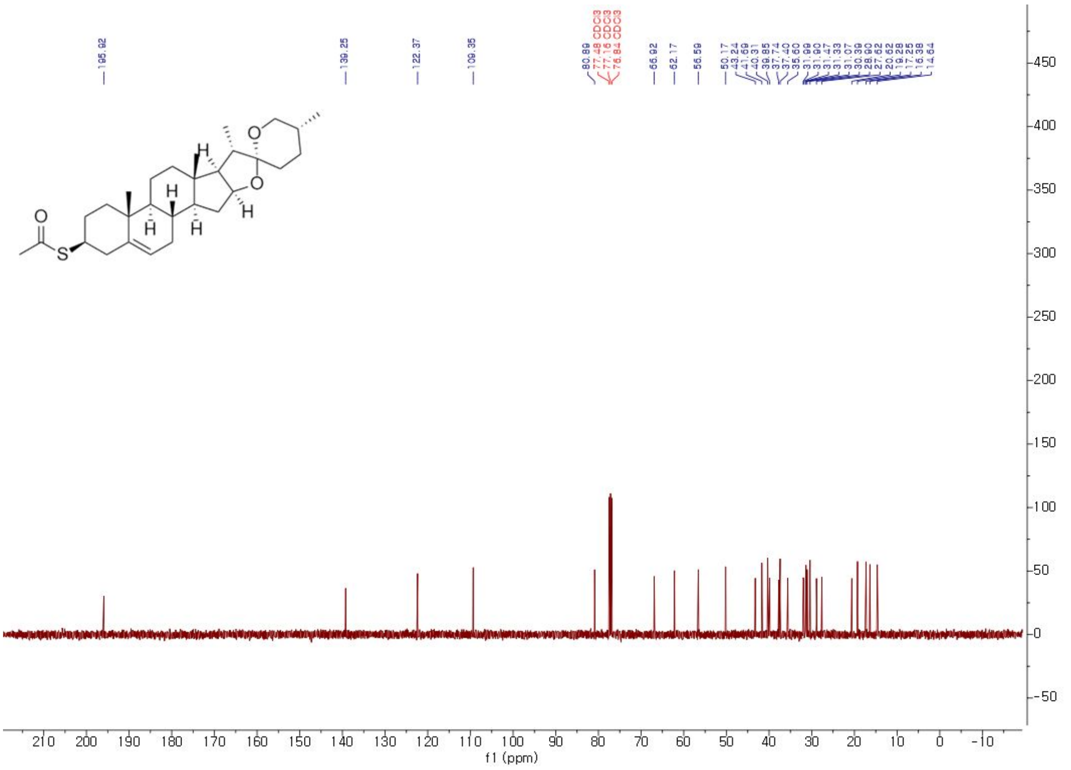

Figure S11. ^{1}H- and ^{13}C-NMR of compound **14**.

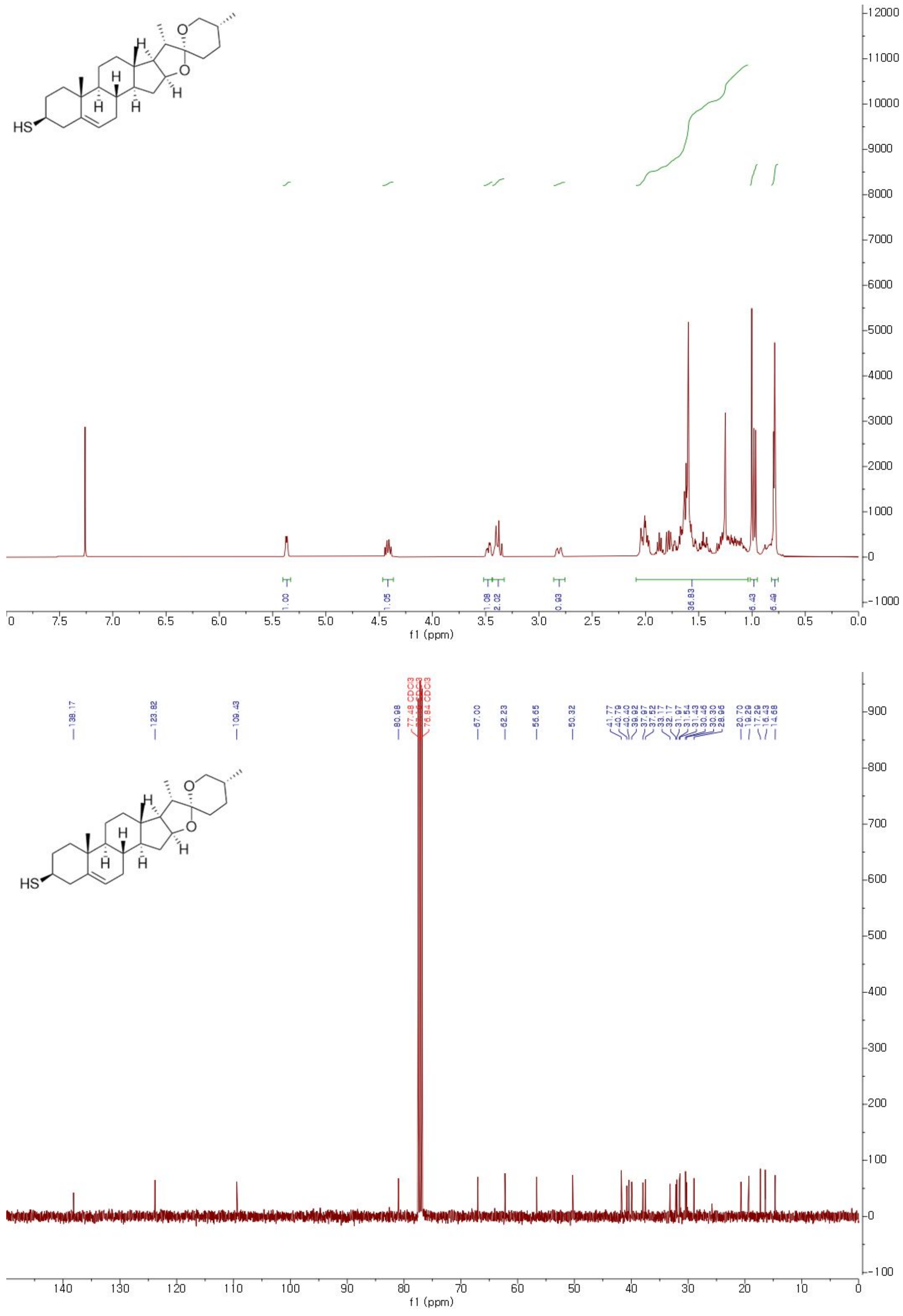

Figure S12. ^{1}H- and ^{13}C-NMR of compound **15**.

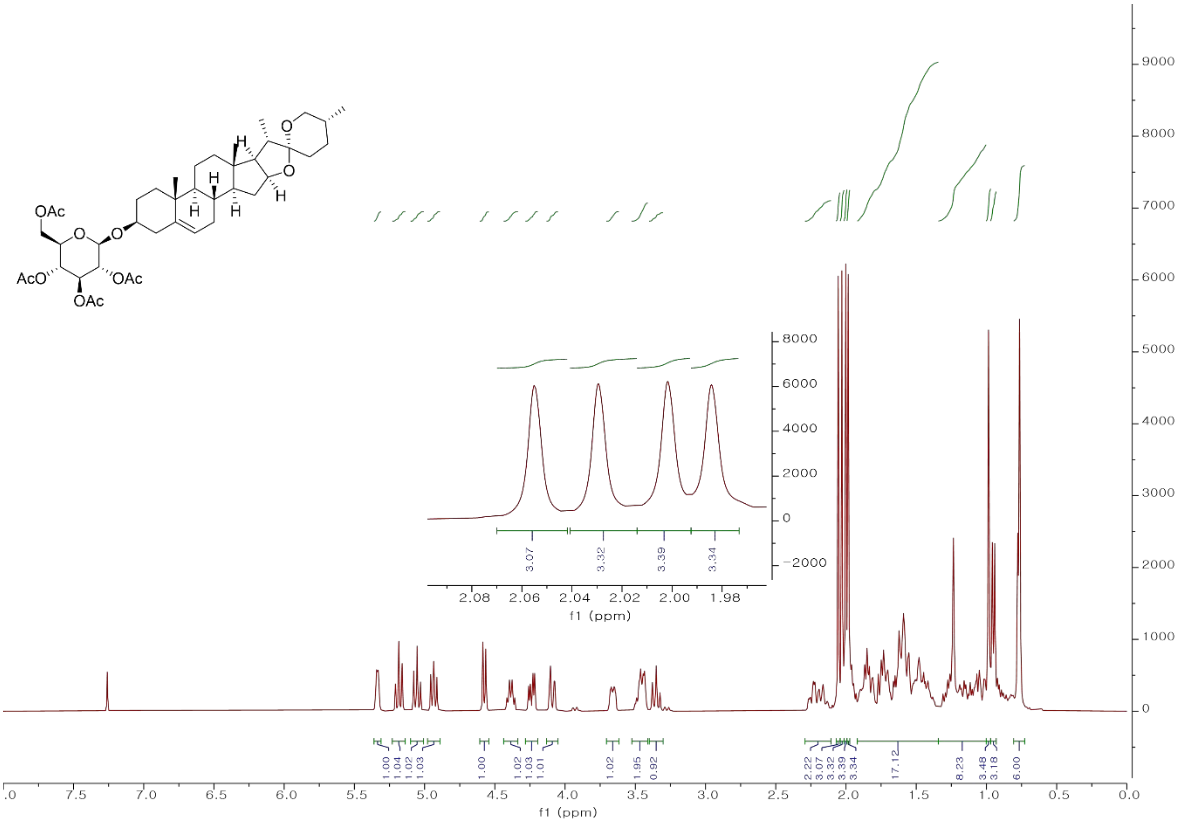

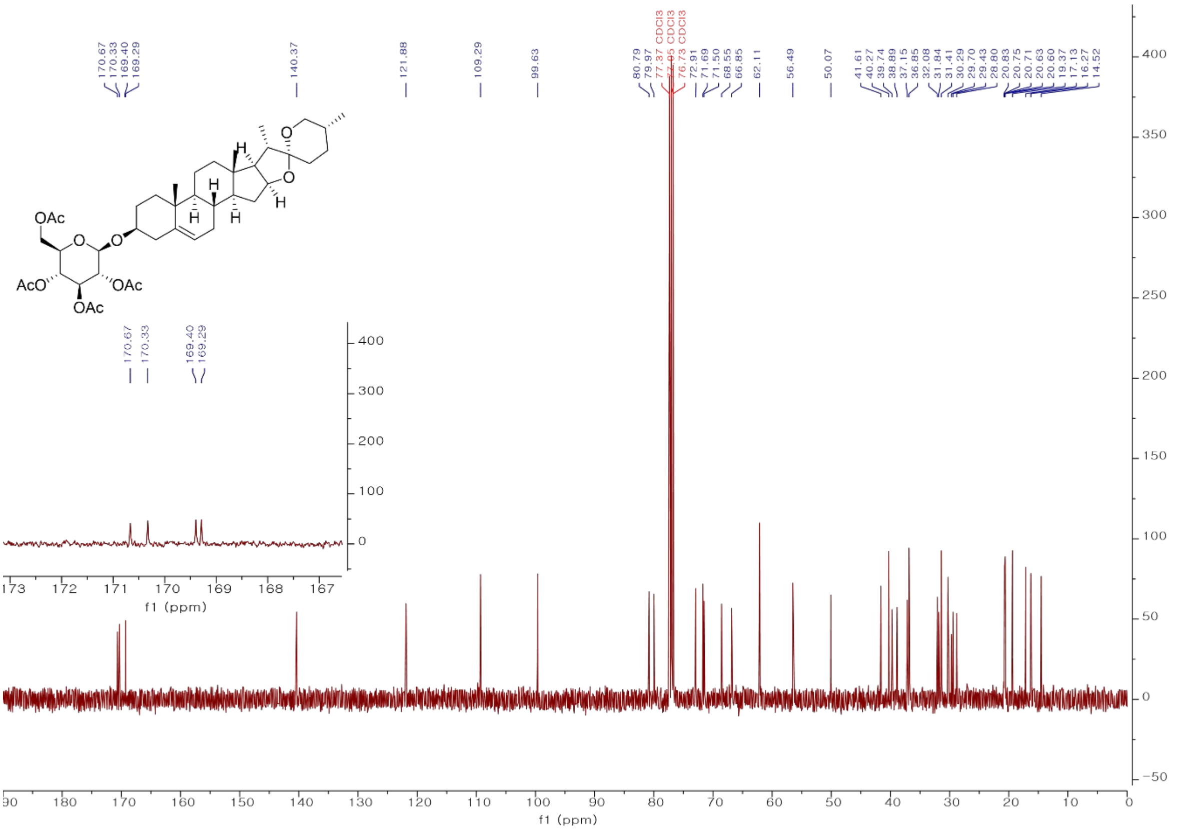

Figure S13. ^{1}H- and ^{13}C-NMR of compound **16**.

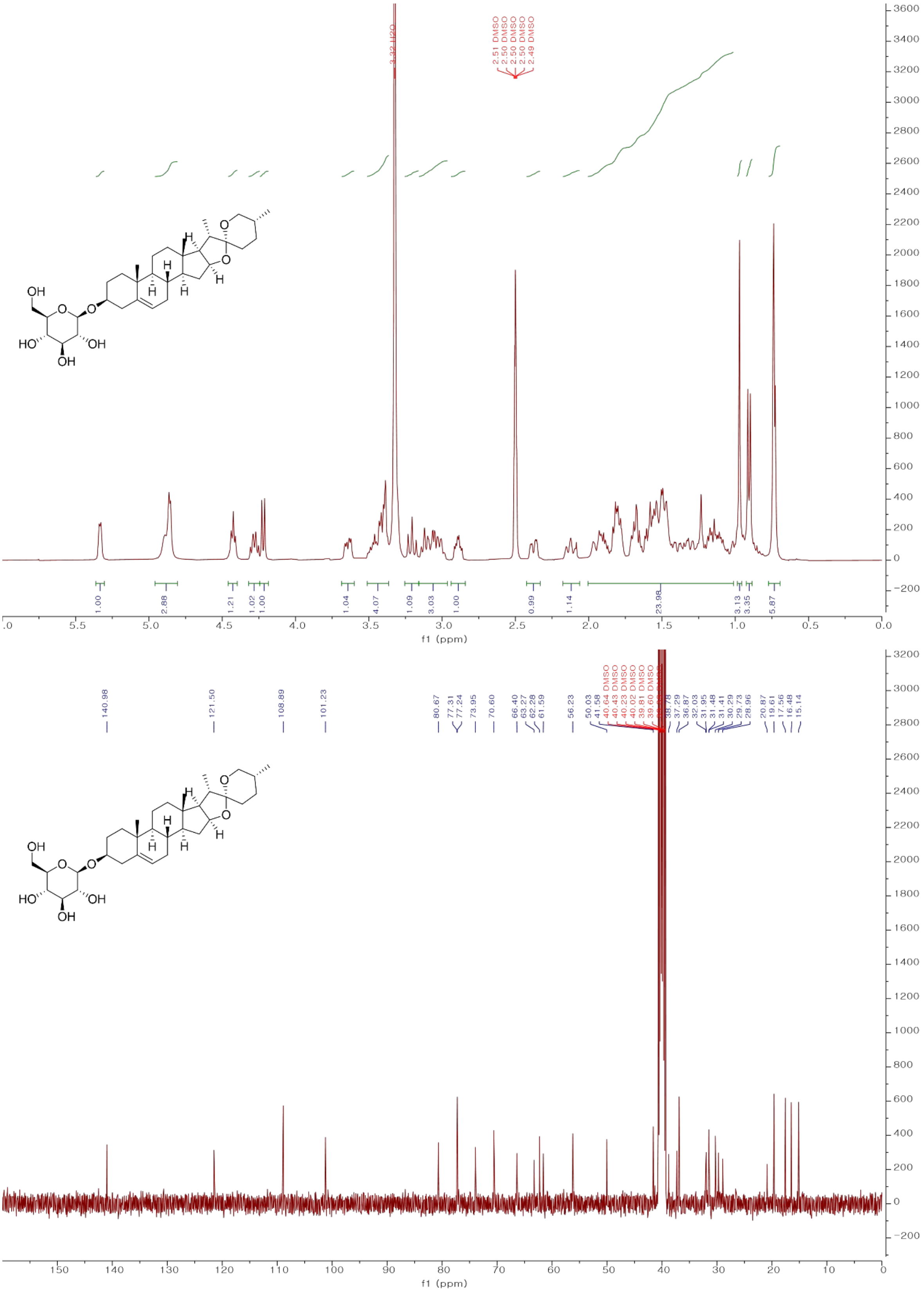

II. Mass Spectra

Figure S14. Compound **5**.

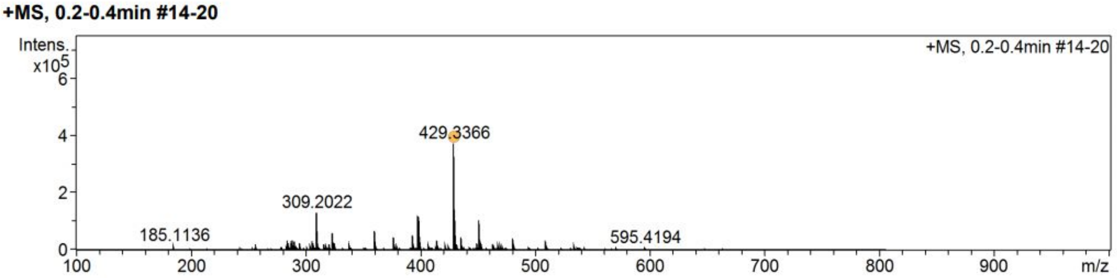

Meas. m/z	#	Ion Formula	m/z	err [ppm]	mSigma	# Sigma	Score	rdb	e¯ Conf	N-Rule
429.3366	1	C28H45O3	429.3363	0.6	1.4	1	100.00	6.5	even	ok
	2	C24H41N6O	429.3336	-6.9	13.7	2	12.57	7.5	even	ok
	1	C26H46NaO3	429.3339	-6.2	12.3	1	100.00	3.5	even	ok

Figure S15. Compound **7**.

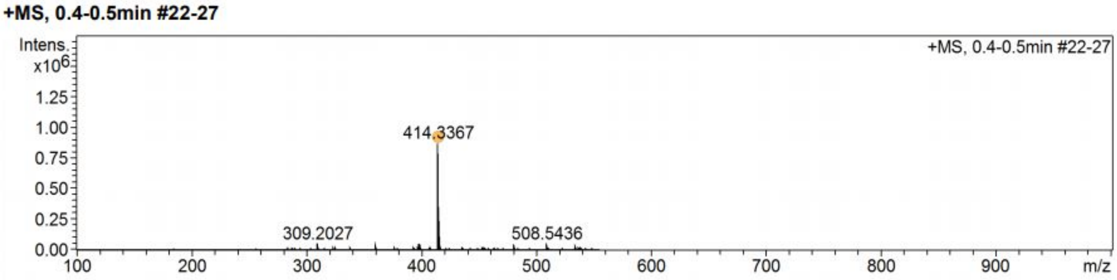

Meas. m/z	#	Ion Formula	m/z	err [ppm]	mSigma	# Sigma	Score	rdb	e¯ Conf	N-Rule
414.3367	1	C27H44NO2	414.3367	-0.1	0.8	1	100.00	6.5	even	ok
	2	C23H40N7	414.3340	-6.6	13.5	2	10.40	7.5	even	ok
	1	C25H45NNaO2	414.3343	6.0	10.6	1	100.00	3.5	even	ok
	2	C15H41N11NaO	414.3388	5.0	55.5	2	33.58	0.5	even	ok

Figure S16. Compound **8**.

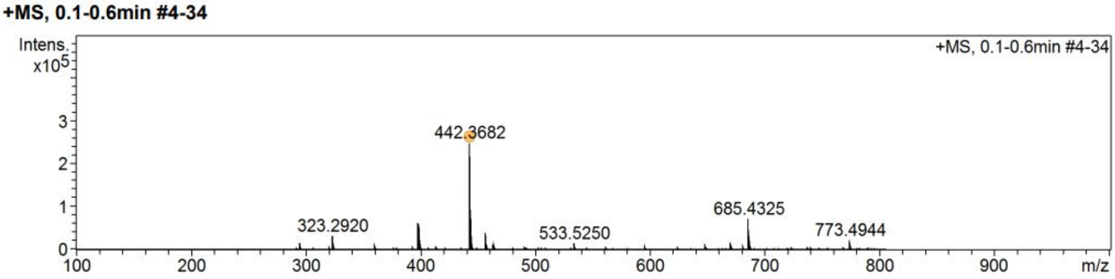

Meas. m/z	#	Ion Formula	m/z	err [ppm]	mSigma	# Sigma	Score	rdb	e¯ Conf	N-Rule
442.3682	1	C29H48NO2	442.3680	0.6	1.9	1	100.00	6.5	even	ok
	2	C25H44N7	442.3653	-6.7	14.3	2	12.43	7.5	even	ok
	1	C27H49NNaO2	442.3656	6.1	12.9	1	100.00	3.5	even	ok

Figure S17. Compound **9**.

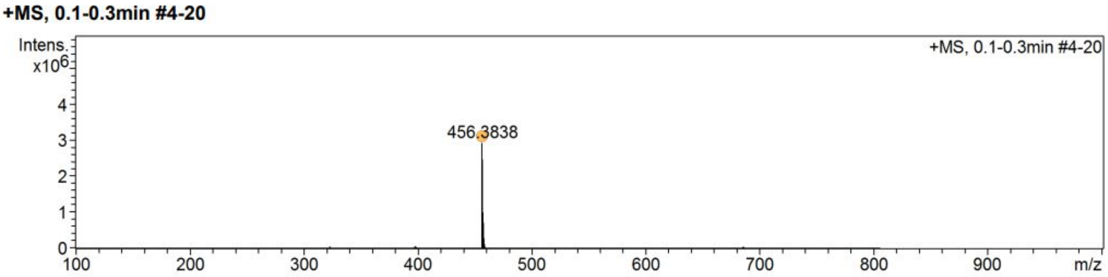

Meas. m/z	#	Ion Formula	m/z	err [ppm]	mSigma	# Sigma	Score	rdb	e⁻ Conf	N-Rule
456.3838	1	C26H46N7	456.3809	-6.3	9.2	1	20.70	7.5	even	ok
	2	C30H50NO2	456.3836	0.4	21.0	2	100.00	6.5	even	ok
	1	C28H51NNaO2	456.3812	-5.7	9.8	1	100.00	3.5	even	ok

Figure S18. Compound **11**.

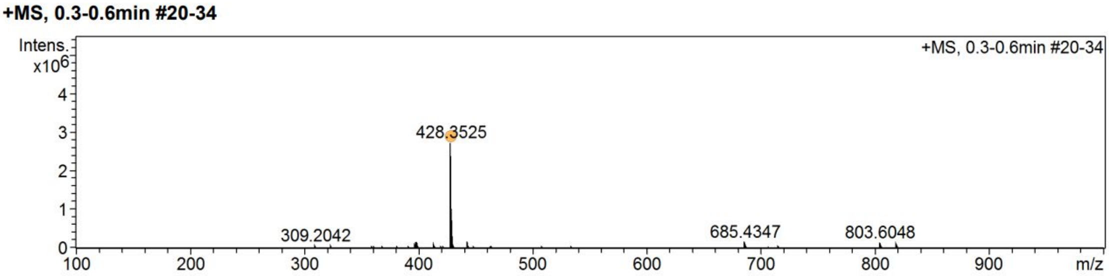

Meas. m/z	#	Ion Formula	m/z	err [ppm]	mSigma	# Sigma	Score	rdb	e⁻ Conf	N-Rule
428.3525	1	C24H42N7	428.3496	6.7	4.0	1	17.57	7.5	even	ok
	2	C28H46NO2	428.3523	0.5	8.6	2	100.00	6.5	even	ok
	1	C26H47NNaO2	428.3499	-6.1	2.7	1	100.00	3.5	even	ok
	2	C16H43N11NaO	428.3544	4.5	46.4	2	48.53	0.5	even	ok

Figure S19. Compound **14**.

+MS, 0.7-1.0min #40-57

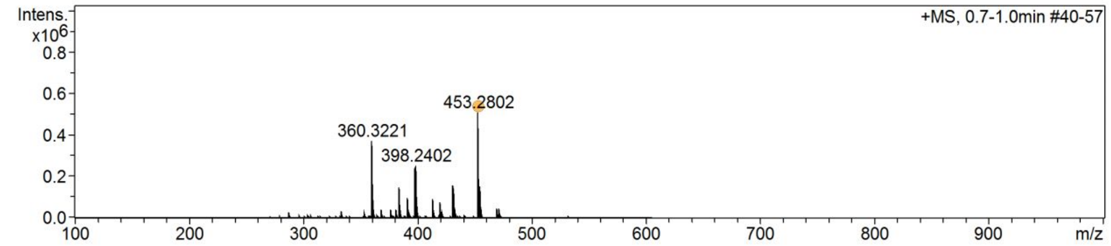

Meas. m/z	#	Ion Formula	m/z	err [ppm]	mSigma	# Sigma	Score	rdb	e^- Conf	N-Rule
453.2802	1	C25H37N6S	453.2795	-1.6	1.5	1	100.00	10.5	even	ok
	2	C29H41O2S	453.2822	4.3	11.3	2	38.33	9.5	even	ok
	3	C24H41N2O4S	453.2782	-4.5	13.1	3	34.30	5.5	even	ok
	4	C22H41N6S2	453.2829	5.8	20.9	4	22.40	5.5	even	ok
	5	C21H45N2O4S2	453.2815	-2.9	28.7	5	51.73	0.5	even	ok
	1	C27H42NaO2S	453.2798	1.0	2.0	1	100.00	6.5	even	ok
	2	C23H38N6NaS	453.2771	-6.9	12.7	2	11.79	7.5	even	ok
	3	C24H46NaO2S2	453.2831	-6.5	22.3	3	14.71	1.5	even	ok

Figure S20. Compound **16**.

+MS, 0.5-1.0min #29-56

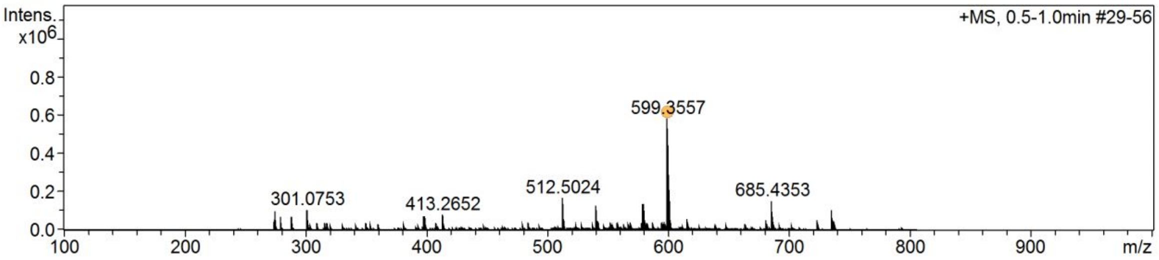

Meas. m/z	#	Ion Formula	m/z	err [ppm]	mSigma	# Sigma	Score	rdb	e^- Conf	N-Rule
599.3557	1	C31H47N6O6	599.3552	-0.8	6.8	1	100.00	11.5	even	ok
	2	C35H51O8	599.3578	3.6	12.0	2	31.34	10.5	even	ok
	3	C30H51N2O10	599.3538	-3.1	12.9	3	39.80	6.5	even	ok
	4	C28H39N16	599.3538	-3.1	13.1	4	39.35	17.5	even	ok
	5	C32H43N10O2	599.3565	-1.4	14.1	5	73.90	16.5	even	ok
	6	C27H43N12O4	599.3525	5.3	16.7	6	11.45	12.5	even	ok
	7	C36H47N4O4	599.3592	5.9	23.7	7	7.01	15.5	even	ok
	8	C42H47O3	599.3520	6.2	49.7	8	2.91	19.5	even	ok
	1	C33H52NaO8	599.3554	0.4	4.7	1	100.00	7.5	even	ok
	2	C30H44N10NaO2	599.3541	2.6	10.9	2	42.40	13.5	even	ok
	3	C34H48N4NaO4	599.3568	1.9	13.7	3	53.94	12.5	even	ok
	4	C29H48N6NaO6	599.3528	-4.9	14.6	4	13.30	8.5	even	ok
	5	C35H44N8Na	599.3581	-4.1	25.0	5	16.19	17.5	even	ok

III. HPLC Spectra

Figure S21. Compound **4**.

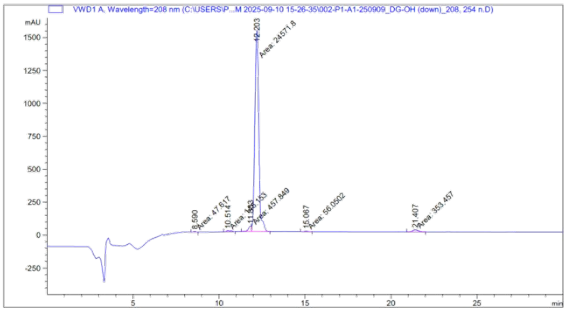

Signal 1: VWD1 A, Wavelength=208 nm

Peak #	RetTime [min]	Type	Width [min]	Area [mAU*s]	Height [mAU]	Area %
1	8.590	MM	0.1702	47.61701	4.66170	0.1858
2	10.514	MM	0.3443	145.15298	7.02705	0.5663
3	11.833	MM	0.1963	457.84949	38.87427	1.7862
4	12.203	MM	0.2685	2.45718e4	1525.27979	95.8640
5	15.067	MM	0.2598	56.05022	3.59528	0.2187
6	21.407	MM	0.3986	353.45657	14.77781	1.3790
Totals :				2.56320e4	1594.21590	

Figure S22. Compound **5**.

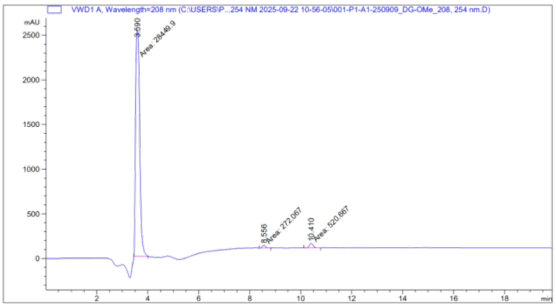

Signal 1: VWD1 A, Wavelength=208 nm

Peak #	RetTime [min]	Type	Width [min]	Area [mAU*s]	Height [mAU]	Area %
1	3.590	MM	0.1870	2.84499e4	2535.00366	97.2891
2	8.556	MM	0.1461	272.06659	31.04017	0.9304
3	10.410	MM	0.1774	520.66650	48.91699	1.7805
Totals :				2.92426e4	2614.96082	

*** End of Report ***

Figure S23. Compound **6**.

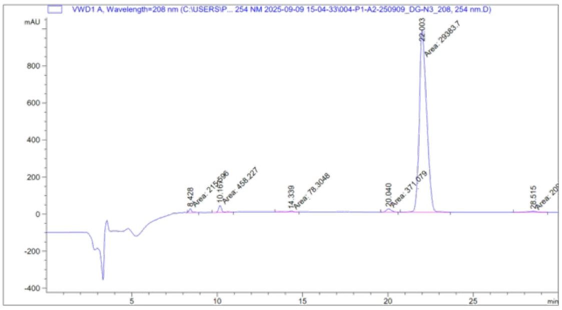

Signal 1: VWD1 A, Wavelength=208 nm

Peak #	RetTime [min]	Type	Width [min]	Area [mAU*s]	Height [mAU]	Area %
1	8.428	MM	0.1758	215.59634	20.44213	0.7019
2	10.167	MM	0.2054	458.22720	37.18325	1.4918
3	14.339	MM	0.2939	78.30484	4.44084	0.2549
4	20.040	MM	0.3528	371.07883	17.52883	1.2081
5	22.003	MM	0.4985	2.93837e4	982.31238	95.6612
6	28.515	MM	0.6713	209.50171	5.20126	0.6821
Totals :				3.07164e4	1067.10868	

*** End of Report ***

Figure S24. Compound **7**.

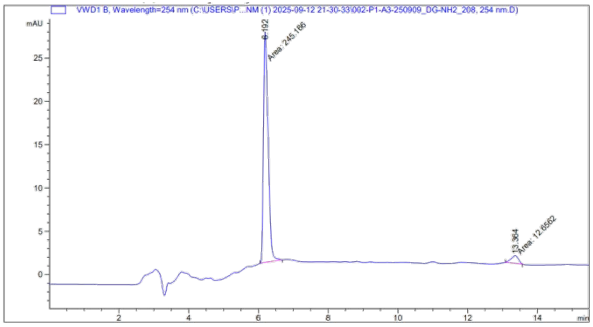

Signal 1: VWD1 B, Wavelength=254 nm

Peak #	RetTime [min]	Type	Width [min]	Area [mAU*s]	Height [mAU]	Area %
1	6.192	MM	0.1535	245.16565	26.62459	95.0911
2	13.364	MM	0.2403	12.65619	8.77946e-1	4.9089
Totals :				257.82184	27.50254	

*** End of Report ***

Figure S25. Compound **8**.

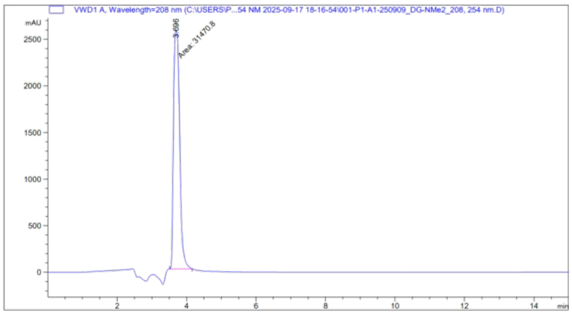

Signal 1: VWD1 A, Wavelength=208 nm

Peak #	RetTime [min]	Type	Width [min]	Area [mAU*s]	Height [mAU]	Area %
1	3.696	MM	0.2053	3.14708e4	2554.49683	100.0000

Totals : 3.14708e4 2554.49683

*** End of Report ***

Figure S26. Compound **9**.

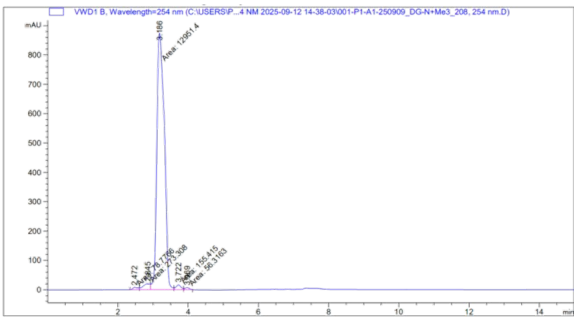

Signal 1: VWD1 B, Wavelength=254 nm

Peak #	RetTime [min]	Type	Width [min]	Area [mAU*s]	Height [mAU]	Area %
1	2.472	MM	0.1776	78.77663	7.39385	0.5829
2	2.845	MM	0.2299	273.30826	19.81254	2.0222
3	3.186	MM	0.2471	1.29514e4	873.58356	95.8283
4	3.722	MM	0.1592	155.41524	16.26874	1.1499
5	3.969	MM	0.1344	56.31627	6.98440	0.4167

Totals : 1.35152e4 924.04308

*** End of Report ***

Figure S27. Compound **11**.

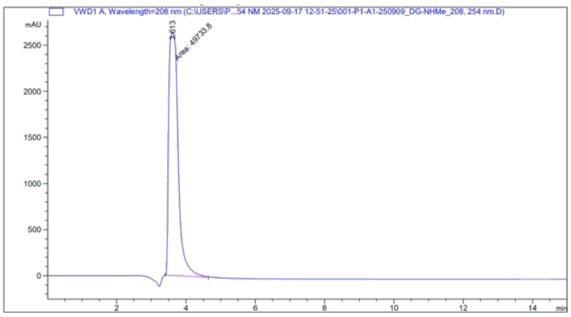

Signal 1: VWD1 A, Wavelength=208 nm

Peak #	RetTime [min]	Type	Width [min]	Area [mAU*s]	Height [mAU]	Area %
1	3.613	MM	0.3152	4.97338e4	2630.12256	100.0000

Totals : 4.97338e4 2630.12256

*** End of Report ***

Figure S28. Compound **12**.

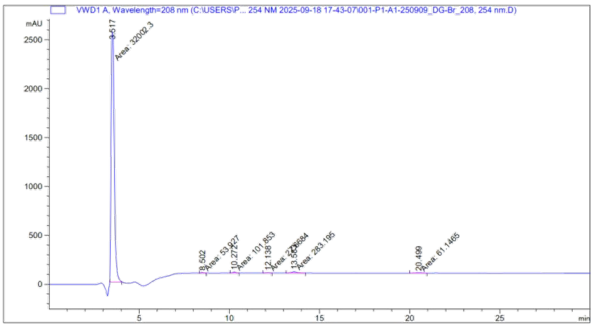

Signal 1: VWD1 A, Wavelength=208 nm

Peak #	RetTime [min]	Type	Width [min]	Area [mAU*s]	Height [mAU]	Area %
1	3.517	MM	0.2087	3.20023e4	2556.03296	98.3775
2	8.502	MM	0.1249	53.92701	7.19488	0.1658
3	10.272	MM	0.1522	101.85347	11.15365	0.3131
4	12.138	MM	0.1892	27.66843	2.43697	0.0851
5	13.583	MM	0.3653	283.19547	12.91985	0.8706
6	20.499	MM	0.3681	61.14648	2.76873	0.1880

Totals : 3.25301e4 2592.50703

*** End of Report ***

Figure S29. Compound **14**.

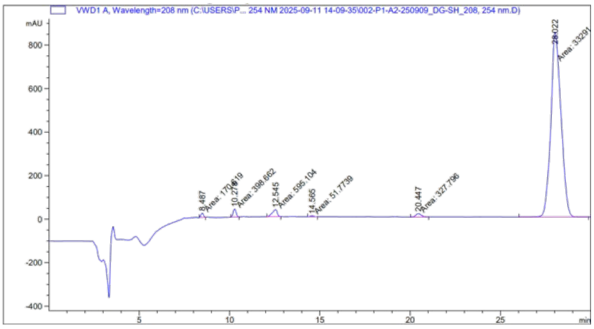

Signal 1: VWD1 A, Wavelength=208 nm

Peak #	RetTime [min]	Type	Width [min]	Area [mAU*s]	Height [mAU]	Area %
1	8.487	MM	0.1490	170.61934	19.08279	0.4898
2	10.278	MM	0.1827	398.66205	36.35976	1.1444
3	12.545	MM	0.3126	595.10413	31.72373	1.7083
4	14.565	MM	0.2400	51.77390	3.59553	0.1486
5	20.447	MM	0.3624	327.79620	15.07616	0.9410
6	28.022	MM	0.6538	3.32911e4	848.60669	95.5678
Totals :				3.48351e4	954.44467	

*** End of Report ***

Figure S30. Compound **16**.

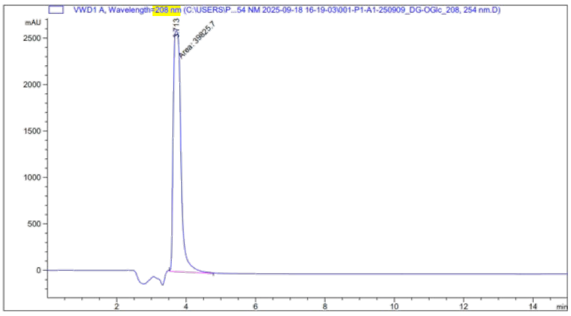

Signal 1: VWD1 A, Wavelength=208 nm

Peak #	RetTime [min]	Type	Width [min]	Area [mAU*s]	Height [mAU]	Area %
1	3.713	MM	0.2563	3.98257e4	2590.24316	100.0000
Totals :				3.98257e4	2590.24316	

*** End of Report ***

IV. *In Vitro* Activity Assay of Diosgenin Derivatives

Figure S31. Detection of NO releasing (left) and cell viability (right).

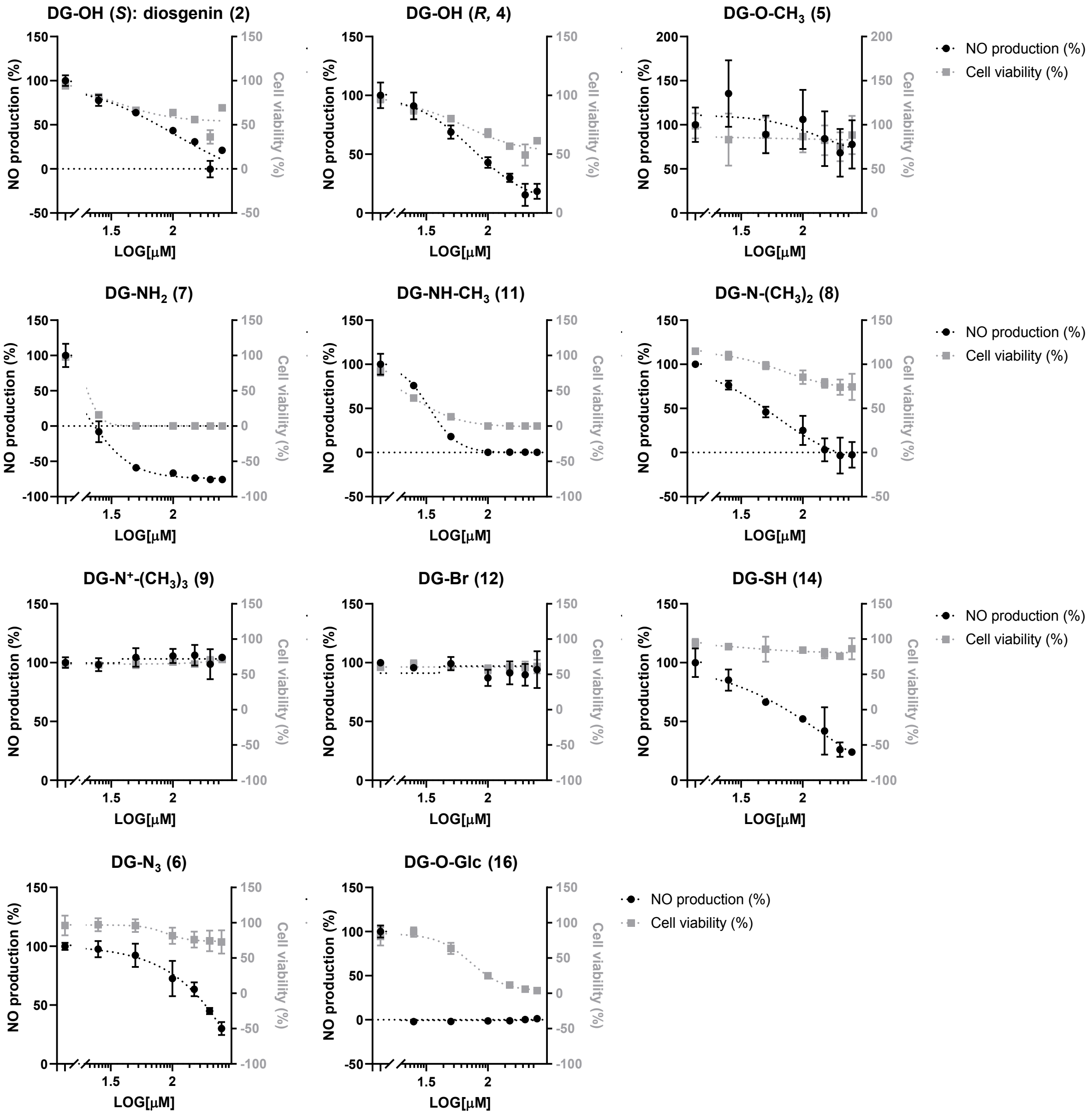
DG-OH (S): diosgenin (2)
DG-OH (R, 4)
DG-O-CH3 (5)
DG-NH2 (7)
DG-NH-CH3 (11)
DG-N-(CH3)2 (8)
DG-N+-(CH3)3 (9)
DG-Br (12)
DG-SH (14)
DG-N3 (6)
DG-O-Glc (16)
NO production (%)
Cell viability (%)
LOG[μM]

V. Cell Viability Tests

Figure S32. Cell viability. BV-2 cells were pre-treated with indicated concentration of compound **8** for 30 min and then treated with LPS (100 ng/mL) for 24 h. Cell viability was assessed with MTS assay and supernatants were used for ELISA analysis shown in figure **2b**.

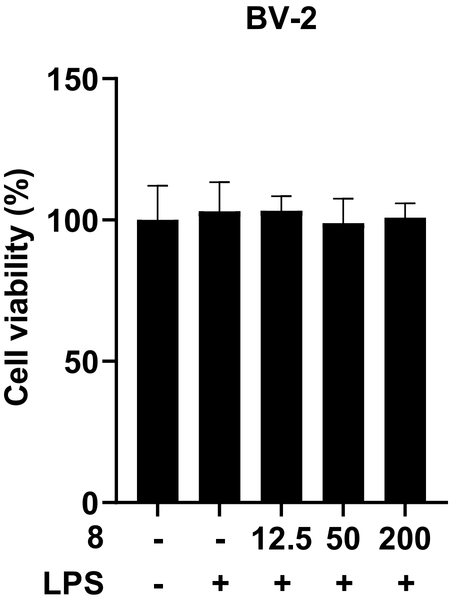

Figure S33. Cell viability of compounds in mouse cell lines. Cells were pre-treated with the indicated concentration of compounds and then treated with LPS (200 ng/mL) for 24 h. Data was normalized by LPS control.

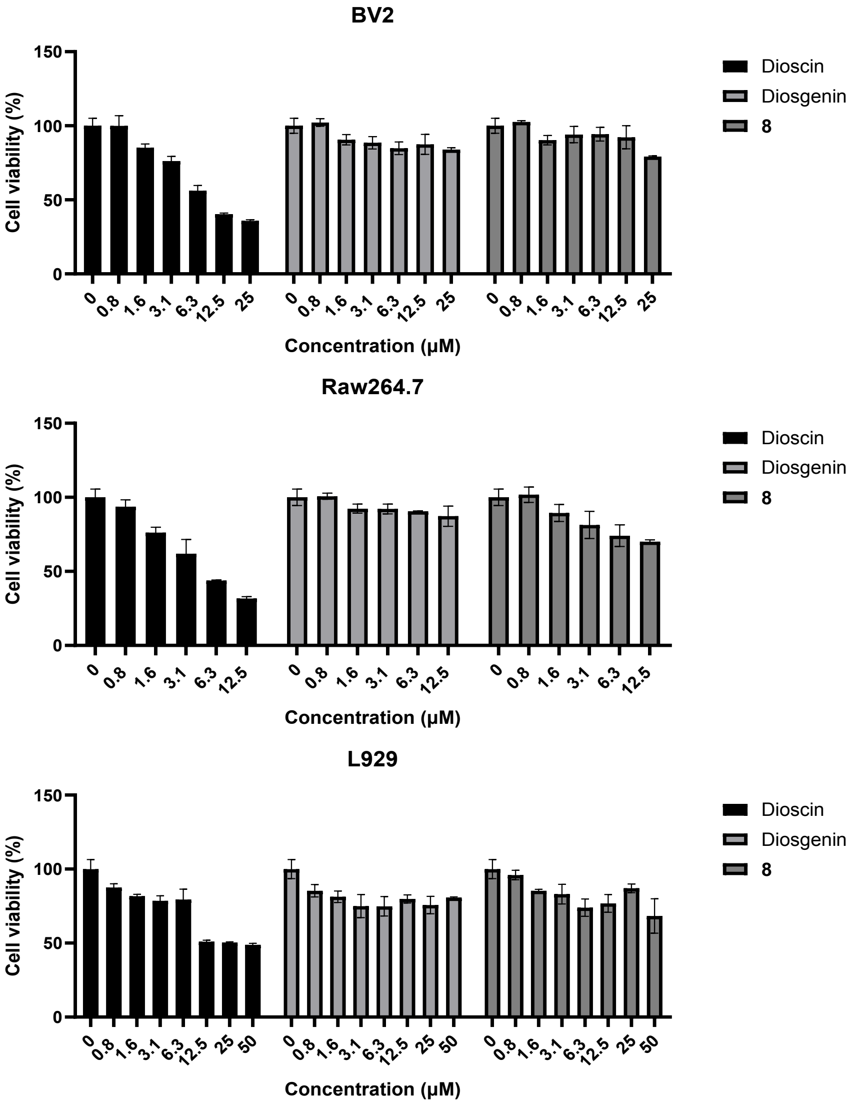

VI. PCR Primer Sequences

Mouse iNOS	forward	5’-GAGACAGGGAAGTCTGAAGCAC-3’
	reverse	5’-CCAGCAGTAGTTGCTCCTCTTC-3’
Mouse HO-1	forward	5’-CACTCTGGAGATGACACCTGAG-3’
	reverse	5’-GTGTTCCTCTGTCAGCATCACC-3’
Mouse IL-1b	forward	5’-TGGACCTTCCAGGATGAGGACA-3’
	reverse	5’-GTTCATCTCGGAGCCTGTAGTG-3’
Mouse 1L-6	forward	5’-TACCACTTCACAAGTCGGAGGC-3’
	reverse	5’-CTGCAAGTGCATCATCGTTGTTC-3’

Mouse GAPDH	forward	5’-CATCACTGCCACCCAGAAGACTG-3’
	reverse	5’-ATGCCAGTGAGCTTCCCGTTCAG-3’

VII. *In Vivo* Pharmacokinetic Studies

	subject	T_{max} (h)	C_{max} (ng/mL)	$T_{1/2}$ (h)	AUC_{last} (h·ng/mL)	$AUC_{0-\infty}$ (h·ng/mL)	Cl (mL/min/kg)	MRT_{inf} (h)	V_{ss} (mL/kg)	F (%)
IV	1	0.083	626.74	29.04	3121.59	6672.03	12.49	39.20	29377.91	
	2	0.083	729.91	21.49	3900.26	7262.33	11.47	30.37	20907.92	
	3	0.083	636.04	21.91	4041.58	7035.47	11.84	28.53	20278.76	
	Mean	0.083	664.23	24.15	3687.81	6989.95	11.94	32.70	23521.53	
	SD	0	57.07	4.24	495.42	297.77	0.51	5.70	5081.52	
IP	1	0.50	1367.84	11.27	17076.89	22355.26		15.82		115.77
	2	0.50	1889.59	13.74	18778.43	27322.15		19.62		127.30
	3	0.50	1465.76	13.48	18293.24	26603.82		19.67		124.01
	Mean	0.50	1574.40	12.83	18049.52	25427.08		18.37		122.36
	SD	0	277.32	1.35	846.56	2684.41		2.21		5.94
PO	1	2.00	1007.89	26.85	16125.75	34275.62		37.95		109.32
	2	2.00	947.97	25.24	15469.88	31701.91		35.81		104.87
	3	2.00	824.93	20.66	13524.53	24902.26		30.01		91.68
	Mean	2.00	926.93	24.25	15040.05	30293.26		34.59		101.96
	SD	0	93.28	3.21	1352.83	4842.85		4.11		9.17

Descriptions of the pharmacokinetic parameters. T_{max}, time to reach Cmax; C_{max}, peak plasma concentration; $T_{1/2,}$ terminal half-life; AUC_{last}, total area under the plasma concentration-time curve from time zero to last measured time; $AUC_{0-\infty}$, total area under the plasma concentration-time curve from time zero to time infinity; Cl, time-averaged total body clearance; MRT, mean residence time; V_{ss}, state volume of distribution; F, oral bioavailability. [a]SD: Standard deviations.

All animal experiments were evaluated and approved by the Medicilon. ICR mice (7~8 weeks of age) weighing 30 ± 5 g were used for the pharmacokinetic and tissue distribution studies. The mice were kept at a room temperature controlled at 23 ± 3 ℃ with relative humidity controlled at about 55 ± 10%, fed with standard solid composite feedstuff, and received tap water. Compound **8** at a dose of 5 mg/kg or 20 mg/kg was administered intravenously or intraperitonealy or orally, respectively, to the ICR mice. Blood samples were collected via carotid artery at 0 (to serve as a control), 0.08 (IV only), 0.25, 0.5, 1, 2, 4, 6, 8, and 24 h after administration of the compound. After centrifugation at 6800 g for 6 min, plasma samples were stored at -80 ℃ until analysis. Pharmacokinetic parameters were determined by a non-compartmental analysis using WinNonlin® 7.0 (Pharsight Corporation, Mountain View, CA) program. The total area under the plasma concentration-time curve from time zero to last measured time (AUC_{last}) was calculated by the trapezoidal rule-extrapolation method. Standard methods were used to calculate the following pharmacokinetic parameters[1,2]; the time-averaged total body clearance (CL), total area under the first moment of plasma concentration and time curve from time zero to time infinity ($AUC_{0-\infty}$), terminal half-life, mean residence time (MRT), apparent volume of distribution at steady state (V_{ss}). Concentrations of the compound in the above samples were analyzed using LC-MS/MS. To a 20 μL aliquot of plasma sample, an 80 μL aliquot of acetonitrile containing 2 μM of internal standard (Chloropropramide) was added. After vortex mixing for 1 min and centrifugation at 14,000 rpm for 7 min, an 8 μL of supernatant was injected into LC-MS/MS system. The LC-MS/MS system consisted of an Agilent 1290 infinity series HPLC system (Agilent, Santa Clara, CA) and API5500® triple-quadrupole mass spectrometer (Applied Biosystems-SCIEX, Concord, Canada). The HPLC mobile phases consisted of 0.1% formic acid in 100% deionized water (A) and 0.1% formic acid in 100% acetonitrile (B). Chromatographic separation was achieved on a reversed-phase ACQUITY UPLC BEH Phenyl C18 column (50 x 2.1 mm, 1.7 μm) using gradient elution at a flow rate of 0.6

mL/min. The lower limit of quantitation of the compound in rat plasma was 2 ng/mL. The values of coefficients of correlation (R) were more than 0.9991.

VIII. Plasma Stability Analysis

Figure S34. Assessment of plasma stability of compound **8**.

Compound type	Time (min)	**compound 8**
		Remaining (%)
Test article	0	100.0
	5	106.9
	30	105.6
	60	74.8
	K	0.0030
	Half-life(T1/2)	231.0
Evaluation		Low
		High metabolism_t1/2 < 30 min<br>Moderate metabolism_t1/2 30 min < t1/2 < 120 min<br>Low metabolism_t1/2 > 120 min
**Final result**		231.0
		Low

Table. Plasma stability (species: mouse) of compound 8

Test compounds (40 μM) were incubated in plasma collected from 7-week-old male ICR mice at 37 °C. At 0, 5, 30, and 60 min, reactions were quenched with acetonitrile:methanol (1:1, v/v), and the remaining parent compounds were analyzed by LC−MS/MS. Analysis was performed using an Agilent 1260 Infinity II LC system coupled to an Agilent InfinityLab LC/MSD (Agilent Technologies), equipped with an Eclipse Plus C18 column (5 μm, 4.6 × 250 mm). The elimination half-life ($t_{1/2}$) was calculated using a first-order kinetic model.

IX. Computational Studies

Figure S35. Predicted binding mode of diosgenin (**2**) within the LY96 binding pocket. Left: 3D docking pose of diosgenin positioned in the hydrophobic cavity of LY96, highlighting interactions with key residues including Ile124, Phe151, and Cys 133. Right:2D interaction diagram illustrating van der Waals and π-alkyl contacts between diosgenin and surrounding residues, with interaction distances (Å) indicated. CDOKER energy and CDOCKER interaction energy values are 54.1078 and -32.6516 kcal/mol, respectively, compared with 60.6756 and -34.1627 kcal/mol for compound **8**.

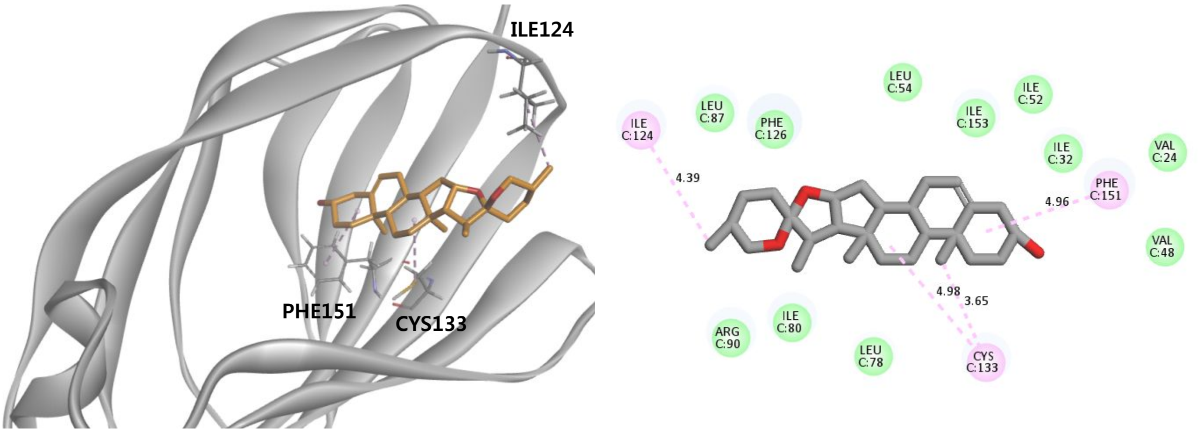

Molecular dynamics (MD) simulations were conducted to investigate the binding stability and interaction profiles of protein complex with compound **8**. The protein-ligand complex, derived from docking result, was prepared using the Discovery Studio 2024. The complex was parameterized with the CHARMm force field and placed in an orthorhombic simulation box, ensuring a minimum distance of 7 Å between the solute and box edges. The system was solvated with explicit periodic boundary, and appropriate counterions were added to neutralize charges. Energy minimization was performed using Conjugate Gradient algorithm and Adopted Basis NR algorithm, followed by heating protocol to reach 300 K. Equilibration was then carried out at a constant temperature (300 K) for 1 ns, and the final configurations were exported in Discovery Studio formats.

Production MD simulations were run for 1 ns under periodic boundary conditions using Discovery Studio 2024. Data were recorded every 2 ps, with long-range electrostatics handled by the Particle-Mesh Ewald (PME) method.[3] The systems were maintained in an NPT ensemble at a constant temperature (300 K) and pressure (1 atm) throughout the simulations.

Trajectory analyses were performed to evaluate the root mean square deviation (RMSD),[4] root mean square fluctuation (RMSF),[5] and key interaction profiles between 3FXI and the ligand (compound **8**). These simulations provided detailed insights into the comparative binding behaviors and interaction dynamics of a ligand with protein.

Figure S36. Molecular dynamics (MD) simulation analysis of the compound **8**-LY96 complex. a) Left: Root-mean-square deviation (RMSD) of LY96, showing convergence to a stable conformation. Right: total energy versus time during MD simulation, indicating energetic stabilization of the system. b) Left: RMSD profile of compound **8**-LY96 complex, showing convergence to a stable conformation. Right: total energy versus time during MD simulation, indicating energetic stabilization of the complex. c) Root-mean-square fluctuation (RMSF) analysis of individual LY96 residues comparing the compound 8-LY 96 complex (blue) and the apo LY96 state (orange), demonstrating reduced flexibility of residues surrounding the binding pocket upon ligand binding.

a)

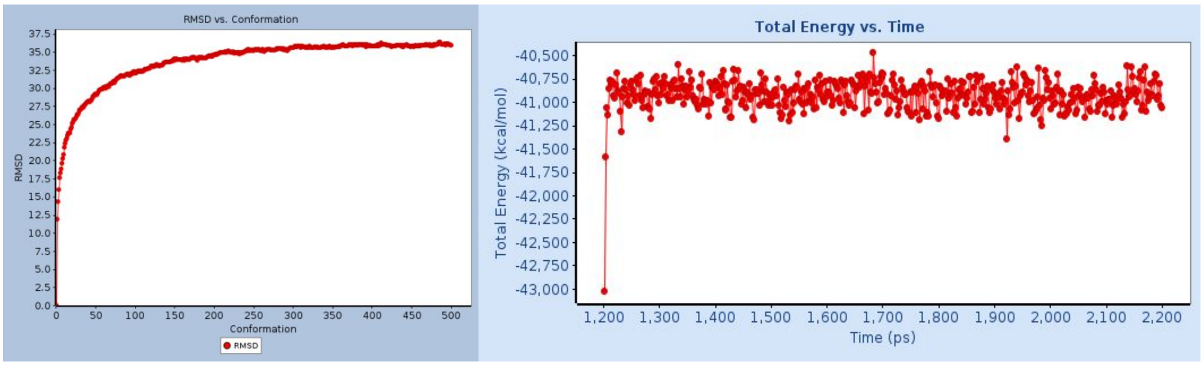

b)

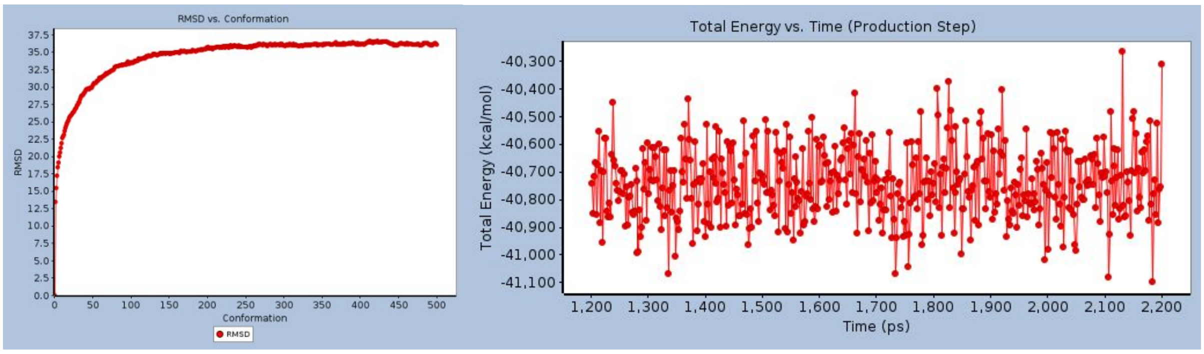

c)

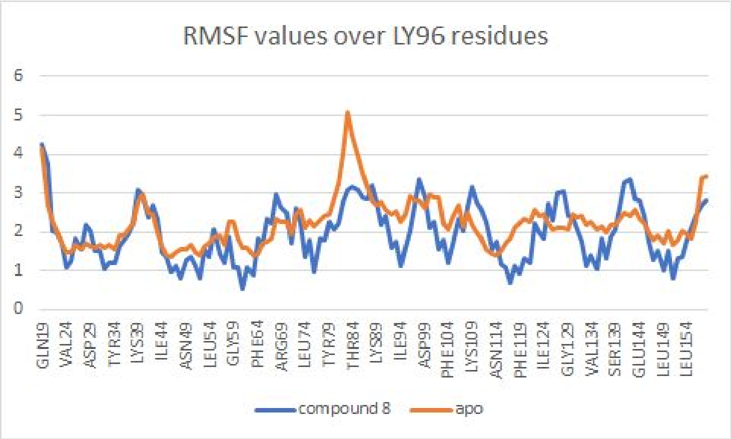

X. SOLUBILITY ANALYSIS

A relative solubility comparison between diosgenin and dimethylamine derivative using analyte-to-internal-standard area ratios under identical experimental conditions (pH 7.4 phosphate buffer, 1% DMSO). Test compounds were first dissolved in DMSO to prepare concentrated stock solutions. These stock solutions were subsequently diluted with phosphate buffer (100 mM, pH 7.4) to obtain the desired final concentrations, with the final DMSO content fixed at 1% (v/v), and an internal standard (IS) added to a to a final concentration of 1.0 µM. The resulting suspensions were incubated with shaking at room temperature for 2 h, followed by centrifugation at 10,000 rpm for 20 min to remove undissolved material. After centrifugation, the supernatant was carefully collected, and the samples were diluted with acetonitrile (ACN) at a ratio of ACN/sample = 3:1 (v/v), followed by filtration through a 20 µm syringe filter. Each sample was prepared in triplicate and analyzed by LC–MS for quantitative determination.

Figure S37. Comparative solubility assessment of diosgenin (**2**) and compound **8**. a) Representative LC-MS area ratios (analyte/internal standard) obtained from three independent experiments at pH 7.4 (100 mM phosphate buffer, 1% DMSO). b) Linear regression of area ratio versus nominal concentration. Left: diosgenin, Right: compound **8**.

a)

1st trial

DG (1st)		IS				
Conc. (µM)	Area	Conc. (µM)	Area		Area ratio	Area ratio / µM
0.1	1,601,667.6370	1.0	14,624,484.1754		0.1095	1.10
0.2	1,911,578.5923	1.0	14,777,860.4922		0.1294	0.65
0.4	1,709,382.5377	1.0	15,079,570.2923		0.1134	0.28
0.6	1,909,035.1735	1.0	14,437,249.4779		0.1322	0.22
0.8	1,894,229.5220	1.0	14,742,926.8023		0.1285	0.16
1.0	1,969,443.6562	1.0	14,977,910.7283		0.1315	0.13
R^2	0.4913			R^2	0.4129	
slope	288591.283			slope	0.018338635	
intercept	1683450.69			interce	0.114597538	
DG 8 (1st)		IS				
Conc. (µM)	Area	Conc. (µM)	Area		Area ratio	Area ratio / µM
1.0	10,701,075.3965	1.0	18,902,647.3587		0.5661	0.57
2.0	20,343,527.1808	1.0	14,480,034.1953		1.4049	0.70
4.0	36,391,771.2673	1.0	13,963,882.4912		2.6061	0.65
6.0	72,575,381.5765	1.0	14,798,420.4692		4.9043	0.82
8.0	59,748,552.3085	1.0	14,191,826.1029		4.2101	0.53
10.0	73,099,904.2265	1.0	14,426,863.0837		5.0669	0.51
R^2	0.8449			R^2	0.8636	
slope	7095077.314			slope	0.503955698	
intercept	8818802.539			interce	0.522637301	

2nd trial

DG (2nd)		IS				
Conc. (µM)	Area	Conc. (µM)	Area		Area ratio	Area ratio / µM
0.1	571,341.2532	1.0	7,058,336.5762		0.0809	0.81
0.2	1,651,504.8167	1.0	14,601,841.7435		0.1131	0.57
0.4	1,675,875.0868	1.0	14,998,898.4510		0.1117	0.28
0.6	1,668,513.8501	1.0	14,728,789.4088		0.1133	0.19
0.8	1,653,101.1381	1.0	14,453,912.0953		0.1144	0.14
1.0	1,678,857.2048	1.0	14,621,738.3133		0.1148	0.11
R^2	0.3519			R^2	0.4039	
slope	759969.8379			slope	0.024267113	
intercept	1090547.809			interce	0.09550425	
DG 8 (2nd)		IS				
Conc. (µM)	Area	Conc. (µM)	Area		Area ratio	Area ratio / µM
1.0	9,347,854.2139	1.0	17,125,501.1869		0.5458	0.55
2.0	19,469,064.0489	1.0	14,163,572.2850		1.3746	0.69
4.0	37,740,079.3150	1.0	14,113,826.6366		2.6740	0.67
6.0	49,347,816.6613	1.0	14,922,840.0466		3.3069	0.55
8.0	61,429,454.2417	1.0	14,476,731.2339		4.2433	0.53
10.0	72,122,126.5284	1.0	14,760,103.7329		4.8863	0.49
R^2	0.9857			R^2	0.9768	
slope	6889842.047			slope	0.470937208	
intercept	5978548.591			interce	0.405305684	

3rd trial

DG (3rd)		IS				
Conc. (µM)	Area	Conc. (µM)	Area		Area ratio	Area ratio / µM
0.1	1,481,885.8340	1.0	14,377,684.1880		0.1031	1.03
0.2	1,568,016.4193	1.0	14,106,198.9791		0.1112	0.56
0.4	1,579,137.1588	1.0	14,574,802.2691		0.1083	0.27
0.6	1,611,820.1442	1.0	13,553,737.5935		0.1189	0.20
0.8	1,593,607.2479	1.0	13,738,715.5113		0.1160	0.14
1.0	1,659,087.7415	1.0	13,632,400.6812		0.1217	0.12
R^2	0.7660			R^2	0.7902	
slope	147138.3702			slope	0.017772868	
intercept	1506237.6			interce	0.104015672	
DG 8 (3rd)		IS				
Conc. (µM)	Area	Conc. (µM)	Area		Area ratio	Area ratio / µM
1.0	8,970,173.0712	1.0	18,366,872.4099		0.4884	0.49
2.0	18,661,130.9143	1.0	13,754,055.6665		1.3568	0.68
4.0	35,624,616.8552	1.0	13,541,093.8106		2.6309	0.66
6.0	45,218,649.6340	1.0	14,256,365.9674		3.1718	0.53
8.0	55,967,883.1935	1.0	13,828,087.7401		4.0474	0.51
10.0	62,143,795.2272	1.0	13,475,595.1011		4.6116	0.46
R^2	0.9696			R^2	0.9671	
slope	5894600.526			slope	0.443825973	
intercept	7308938.766			interce	0.424702828	

b)

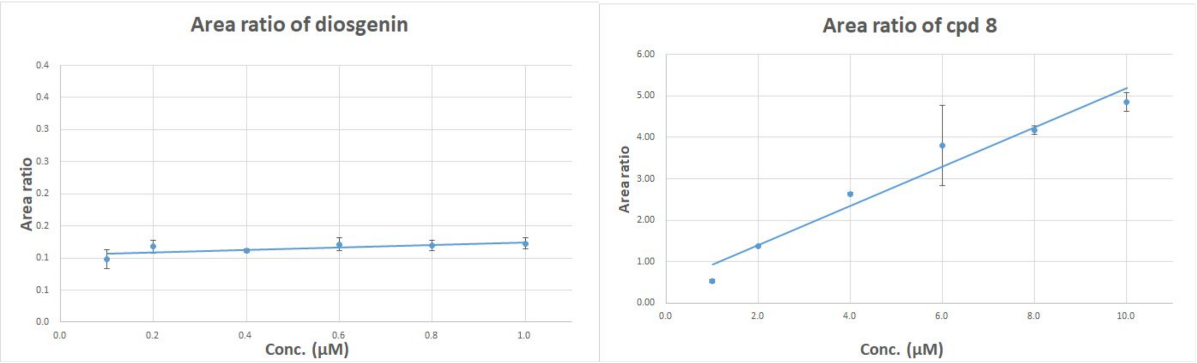

Diosgenin was evaluated over the concentration range of 0.1–1.0 µM, as it exhibited early saturation behavior at low micromolar concentrations, whereas the derivative was analyzed over 1.0–10.0 µM to ensure measurements within the linear, unsaturated range. Accordingly, slope-based analysis of the area ratio versus nominal concentration was employed for quantitative comparison.

Across three independent replicates, diosgenin showed shallow slopes ranging from 0.018 to 0.024, accompanied by relatively low R^2 values (0.40–0.79), consistent with plateau-like behavior indicative of limited apparent solubility. In contrast, the dimethylamine-containing derivative exhibited markedly steeper slopes (0.44–0.50) with high linearity (R^2 = 0.86–0.98), reflecting a substantially higher apparent solubility.

XI. REFERENCES

1. Gibaldi, M.; Perrier, D. *Pharmacokinetics*. 2nd ed., Marcel-Dekker, New York, **1982**.

2. Kerns, E. H.; Di, L. *Drug-like Properties: Concepts, Structure Design and Methods: From ADME to Toxicity Optimization*, 1st ed., Academic Press, UK, **2008**.

3. J. Huang, S. Rauscher, G. Nawrocki, T. Ran, M. Feig, B. L. de Groot, H. Grubmüller, A. D. MacKerell Jr, *Nat. Methods* **2017**, *14*, 71–73.

4. Kroemer, R. T.; Vulpetti, A.; McDonald, J. J.; Rohrer, D. C.; Trosset, J. Y.; Giordanetto, F.; Cotesta, S.; McMartin, C.; Kihlen, M.; Stouten, P. F. Assessment of docking poses: interactions-based accuracy classification (IBAC) versus crystal structure deviations. *J. Chem. Inf. Comput. Sci.* **2004**, *44* (3), 871-881.

5. Dong, Y. W.; Liao, M. L.; Meng, X. L.; Somero, G. N. Structural flexibility and protein adaptation to temperature: Molecular dynamics analysis of malate dehydrogenases of marine molluscs. *Proc. Natl. Acad. Sci. USA* **2018**, *115* (6), 1274-1279.
